# Pathobiological signatures of dysbiotic lung injury in pediatric patients undergoing stem cell transplantation

**DOI:** 10.1038/s41591-024-02999-4

**Published:** 2024-05-23

**Authors:** Matt S. Zinter, Christopher C. Dvorak, Madeline Y. Mayday, Gustavo Reyes, Miriam R. Simon, Emma M. Pearce, Hanna Kim, Peter J. Shaw, Courtney M. Rowan, Jeffrey J. Auletta, Paul L. Martin, Kamar Godder, Christine N. Duncan, Nahal R. Lalefar, Erin M. Kreml, Janet R. Hume, Hisham Abdel-Azim, Caitlin Hurley, Geoffrey D. E. Cuvelier, Amy K. Keating, Muna Qayed, James S. Killinger, Julie C. Fitzgerald, Rabi Hanna, Kris M. Mahadeo, Troy C. Quigg, Prakash Satwani, Paul Castillo, Shira J. Gertz, Theodore B. Moore, Benjamin Hanisch, Aly Abdel-Mageed, Rachel Phelan, Dereck B. Davis, Michelle P. Hudspeth, Greg A. Yanik, Michael A. Pulsipher, Imran Sulaiman, Leopoldo N. Segal, Birgitta A. Versluys, Caroline A. Lindemans, Jaap J. Boelens, Joseph L. DeRisi, the Pediatric Transplantation and Cell Therapy Consortium

**Affiliations:** 1 Division of Critical Care Medicine, Department of Pediatrics, University of California, San Francisco, San Francisco, CA USA; 2 Division of Allergy, Immunology, and Bone Marrow Transplantation, Department of Pediatrics, University of California, San Francisco, San Francisco, CA USA; 3 Departments of Laboratory Medicine and Pathology, Yale School of Medicine, New Haven, CT USA; 4 The Children’s Hospital at Westmead, Sydney, New South Wales Australia; 5 Department of Pediatrics, Division of Critical Care Medicine, Indiana University, Indianapolis, IN USA; 6 Hematology/Oncology/BMT and Infectious Diseases, Nationwide Children’s Hospital, Columbus, OH USA; 7 Center for International Blood and Marrow Transplant Research, National Marrow Donor Program/Be The Match, Minneapolis, MN USA; 8 Division of Pediatric and Cellular Therapy, Duke University Medical Center, Durham, NC USA; 9 Cancer and Blood Disorders Center, Nicklaus Children’s Hospital, Miami, FL USA; 10 Division of Pediatric Oncology Harvard Medical School Department of Pediatrics, Dana-Farber Cancer Institute and Boston Children’s Hospital, Boston, MA USA; 11 Division of Pediatric Hematology/Oncology, Benioff Children’s Hospital Oakland, University of California, San Francisco, Oakland, CA USA; 12 Department of Child Health, Division of Critical Care Medicine, University of Arizona, Phoenix, AZ USA; 13 Department of Pediatrics, Division of Critical Care Medicine, University of Minnesota, Minneapolis, MN USA; 14 Department of Pediatrics, Division of Hematology/Oncology and Transplant and Cell Therapy, Keck School of Medicine, University of Southern California, Los Angeles, CA USA; 15 Loma Linda University School of Medicine, Cancer Center, Children Hospital and Medical Center, Loma Linda, CA USA; 16 Department of Pediatric Medicine, Division of Critical Care, St Jude Children’s Research Hospital, Memphis, TN USA; 17 CancerCare Manitoba, Manitoba Blood and Marrow Transplant Program, University of Manitoba, Winnipeg, Manitoba Canada; 18 Center for Cancer and Blood Disorders, Children’s Hospital Colorado and University of Colorado, Aurora, CO USA; 19 Aflac Cancer & Blood Disorders Center, Children’s Healthcare of Atlanta and Emory University, Atlanta, GA USA; 20 Department of Pediatrics, Division of Pediatric Critical Care, Weill Cornell Medicine, New York, NY USA; 21 Department of Anesthesiology and Critical Care, Perelman School of Medicine, Children’s Hospital of Philadelphia, University of Pennsylvania, Philadelphia, PA USA; 22 Department of Pediatric Hematology, Oncology and Blood and Marrow Transplantation, Pediatric Institute, Cleveland Clinic, Cleveland, OH USA; 23 Department of Pediatrics, Division of Hematology/Oncology, MD Anderson Cancer Center, Houston, TX USA; 24 Pediatric Blood and Marrow Transplantation Program, Texas Transplant Institute, Methodist Children’s Hospital, San Antonio, TX USA; 25 Section of Pediatric BMT and Cellular Therapy, Helen DeVos Children’s Hospital, Grand Rapids, MI USA; 26 Department of Pediatrics, Division of Pediatric Hematology, Oncology and Stem Cell Transplantation, Columbia University, New York, NY USA; 27 UF Health Shands Children’s Hospital, University of Florida, Gainesville, FL USA; 28 Department of Pediatrics, Division of Critical Care Medicine, Joseph M Sanzari Children’s Hospital at Hackensack University Medical Center, Hackensack, NJ USA; 29 Department of Pediatrics, Division of Critical Care Medicine, St. Barnabas Medical Center, Livingston, NJ USA; 30 Department of Pediatric Hematology-Oncology, Mattel Children’s Hospital, University of California, Los Angeles, Los Angeles, CA USA; 31 Department of Pediatrics, Division of Infectious Diseases, Children’s National Hospital, Washington DC, USA; 32 Department of Pediatrics, Division of Pediatric Hematology/Oncology/BMT, Medical College of Wisconsin, Milwaukee, WI USA; 33 Department of Pediatrics, Hematology/Oncology, University of Mississippi Medical Center, Jackson, MS USA; 34 Adult and Pediatric Blood & Marrow Transplantation, Pediatric Hematology/Oncology, Medical University of South Carolina Children’s Hospital/Hollings Cancer Center, Charleston, SC USA; 35 Pediatric Blood and Bone Marrow Transplantation, Michigan Medicine, University of Michigan, Ann Arbor, MI USA; 36 Division of Hematology, Oncology, Transplantation, and Immunology, Primary Children’s Hospital, Huntsman Cancer Institute, Spense Fox Eccles School of Medicine at the University of Utah, Salt Lake City, UT USA; 37 Department of Respiratory Medicine, Royal College of Surgeons in Ireland, Dublin, Ireland; 38 Department of Medicine, Division of Pulmonary and Critical Care Medicine, Laura and Isaac Perlmutter Cancer Center, New York University Grossman School of Medicine, New York University Langone Health, New York, NY USA; 39 Department of Stem Cell Transplantation, Princess Máxima Center for Pediatric Oncology, Utrecht, the Netherlands; 40 Division of Pediatrics, University Medical Center Utrecht, Utrecht, the Netherlands; 41 Transplantation and Cellular Therapy, MSK Kids, Department of Pediatrics, Memorial Sloan Kettering Cancer Center, New York, NY USA; 42 Department of Biochemistry and Biophysics, University of California, San Francisco, San Francisco, CA USA; 43 Chan Zuckerberg Biohub, San Francisco, CA USA

**Keywords:** Immunopathogenesis, Respiratory tract diseases, Translational research, Respiratory signs and symptoms, Infection

## Abstract

Hematopoietic cell transplantation (HCT) uses cytotoxic chemotherapy and/or radiation followed by intravenous infusion of stem cells to cure malignancies, bone marrow failure and inborn errors of immunity, hemoglobin and metabolism. Lung injury is a known complication of the process, due in part to disruption in the pulmonary microenvironment by insults such as infection, alloreactive inflammation and cellular toxicity. How microorganisms, immunity and the respiratory epithelium interact to contribute to lung injury is uncertain, limiting the development of prevention and treatment strategies. Here we used 278 bronchoalveolar lavage (BAL) fluid samples to study the lung microenvironment in 229 pediatric patients who have undergone HCT treated at 32 children’s hospitals between 2014 and 2022. By leveraging paired microbiome and human gene expression data, we identified high-risk BAL compositions associated with in-hospital mortality (*P* = 0.007). Disadvantageous profiles included bacterial overgrowth with neutrophilic inflammation, microbiome contraction with epithelial fibroproliferation and profound commensal depletion with viral and staphylococcal enrichment, lymphocytic activation and cellular injury, and were replicated in an independent cohort from the Netherlands (*P* = 0.022). In addition, a broad array of previously occult pathogens was identified, as well as a strong link between antibiotic exposure, commensal bacterial depletion and enrichment of viruses and fungi. Together these lung–immune system–microorganism interactions clarify the important drivers of fatal lung injury in pediatric patients who have undergone HCT. Further investigation is needed to determine how personalized interpretation of heterogeneous pulmonary microenvironments may be used to improve pediatric HCT outcomes.

## Abstract (web summary)

By leveraging paired microbiome and human gene expression data from pediatric patients who underwent hematopoietic cell transplantation, distinct lung–immune system–microorganism interactions were identified as important drivers of fatal lung injury.

## Main

Hematopoietic cell transplantation (HCT) involves high-dose chemotherapy and/or radiation followed by infusion of hematopoietic progenitor cells with the intention of correcting cellular defects, rescuing chemotherapy-ablated marrow or eradicating malignancy^1^. HCT is often the only curative therapy for patients with malignancy, bone marrow failure and inborn errors of immunity, hemoglobin and metabolism. However, direct chemotherapy toxicity, opportunistic infection and alloreactive inflammation can cause pulmonary injury in up to 40% of patients^2–4^, with hospital mortality rates approaching 50% when mechanical ventilation is required^5,6^.

As such, a deeper understanding of the pulmonary microenvironment is needed to develop next-generation diagnostics and treatments that will improve survival rates. The lung microenvironment harbors complex interactions between pulmonary microorganisms, immunity and the lung epithelium and stroma. We and others have shown that the lungs are not sterile, and in fact contain a variety of microorganisms of varying pathogenic potential that continually populate the lung via inhalation, aspiration, and, in some cases, hematogenous spread^7–9^. Lung sampling through bronchoscopic bronchoalveolar lavage (BAL) is used clinically to detect common pathogens; however, many pathogens evade detection because of preceding antimicrobial treatment, lack of serological immunity in the post-HCT setting or limited preselected targets on multiplex assays, all of which may lead to delayed or missed diagnoses and prolonged broad-spectrum antimicrobial exposure^10^. In addition, organisms of indeterminate clinical importance or context-dependent virulence are frequently identified, leading to questions about the structure, composition and significance of broader microbial communities in this population^8,11^.

We previously reported that in a cohort of children preparing to undergo allogeneic HCT, both pulmonary microbial depletion and pathogen enrichment were associated with poor lung function, concomitant inflammation and the eventual development of fatal post-HCT lung disease^12,13^. To expand these findings to the post-HCT setting, we applied metatranscriptomic sequencing to BAL from prospectively enrolled pediatric patients who have undergone HCT to characterize the pulmonary microbiome landscape, closely monitor occult infections and capture lung gene expression profiles. Overall, we found that depletion of commensal microbiome constituents was associated with pathogen enrichment, inflammation, fibroproliferation and poor survival. Our results suggest pathobiological signatures of dysbiotic lung injury that could be adapted into next-generation diagnostics and eventually leveraged in therapeutic pipelines to improve health outcomes.

## Results

### Patients

**Fig. 1:**
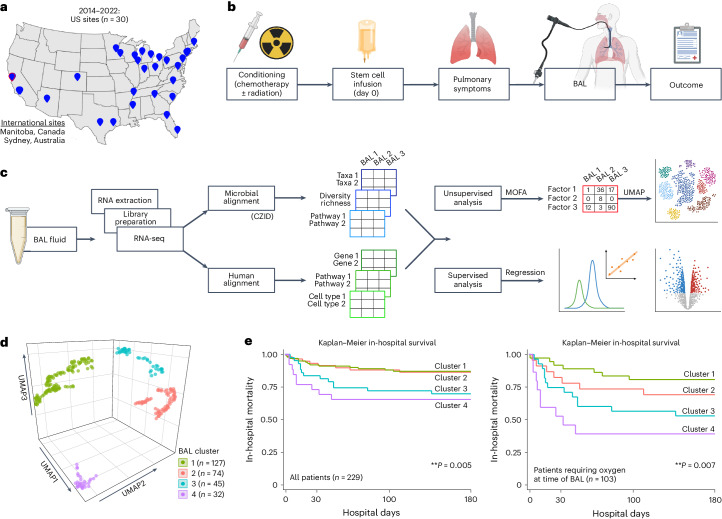
Study design and clinical outcomes. **a**, Patients were recruited from 32 participating children’s hospitals in the United States, Canada and Australia. **b**, Study design diagram. **c**, BAL processing and analysis workflow. **d**, Four microbiome–transcriptome clusters were identified. **e**, In-hospital survival for all patients (left) and the subset requiring respiratory support before testing (right) was plotted according to BAL cluster; differences were analyzed with the log-rank test.

We enrolled 229 pediatric recipients of HCT across 32 children’s hospitals in the United States, Canada and Australia who underwent 278 clinically indicated BAL between 2014 and 2022 (Fig. 1a,b and Table 1). Pulmonary symptoms developed or worsened a median 93 days after HCT (interquartile range (IQR) = 23–278) and were frequently associated with hypoxia and abnormal chest imaging, often in the setting of comorbidities such as graft-versus-host disease (GVHD) and sepsis. BAL was performed a median 112 days after HCT (IQR = 36–329), at which point lymphopenia was prevalent (median absolute lymphocyte count (ALC) = 420 cells per microliter, IQR = 156–1,035). Based on the BAL results, cases were classified as lower respiratory tract infection, non-pulmonary sepsis or idiopathic pneumonia syndrome (*n* = 116, 7 and 155, respectively). After each patient’s most recent BAL, 121 of 229 patients required intensive care (53%), 71 required 7 or more days of mechanical ventilation (31%) and 45 died in the hospital (20%).

**Table 1:** Patient characteristics

**Demographics (*n*** = **229)**	
Age, median years (IQR)	11.0 (4.7–16.7)
Sex, male (%)	133 (58.15)
Ethnicity, *n* (%)	
White	140 (61.1)
Black	29 (12.7)
Other or multiple	26 (11.4)
Asian or Pacific Islander	25 (10.9)
Native American	2 (0.9)
Unknown	7 (3.1)
Ethnic group, *n* (%)	
Latino or Hispanic	59 (25.8)
**Medical history (*n*** = **229)**	
Disease, *n* (%)	
Leukemia^a^	125 (54.6)
Inborn errors of immunity^b^	40 (17.5)
Nonmalignant hematological^c^	27 (11.8)
Solid tumor^d^	14 (6.1)
Lymphoma^e^	12 (5.2)
Inborn errors of metabolism^f^	11 (4.8)
HCT type, *n* (%)	
Allogeneic	213 (93.0)
Bone marrow	92 (43.2)
Peripheral blood	88 (41.3)
UCB	33 (15.5)
Autologous	16 (7.0)
HLA match (allogeneic only), *n* (%)	
Matched related donor	45 (21.1)
Matched unrelated donor (including 6/6 UCB)	49 (23.0)
Mismatched related donor (haplotype)	57 (26.8)
Mismatched unrelated donor (including <6/6 UCB)	62 (29.1)
Conditioning agents used, *n* (%)^g^	
Backbone agent	
Busulfan	86 (37.6)
Melphalan	146 (63.8)
Total body irradiation	63 (27.5)
Other^h^	20 (8.7)
Other alkylating agent	
Cyclophosphamide	91 (39.7)
Thiotepa	66 (28.8)
Antimetabolite	
Clofarabine	15 (6.6)
Cytarabine	5 (2.2)
Fludarabine	146 (63.8)
Serotherapy (anti-thymocyte globulin or alemtuzumab)	119 (52.0)
**Characteristics at enrollment (*n*** = **278 events with BAL)**	
Days from HCT to BAL, median (IQR)	114 (36–331)
Days from symptoms to BAL^i^, median (IQR)	8 (IQR 2–21)
Clinical presentation symptoms, *n* (%)	
Lower respiratory symptoms (e.g., cough, tachypnea)^j^	249 (89.7)
Hypoxia ≤96%	202 (72.7)
Abnormal chest X-ray^k^	174/207 (84.1)
Abnormal chest CT^l^	209/218 (95.9)
Worsening PFTs	16 (5.8)
Respiratory support before BAL, *n* (%)	
No oxygen	156 (56)
Nasal cannula or face mask	41 (15)
High-flow nasal cannula	19 (7)
Noninvasive positive pressure (CPAP or BiPAP)	10 (4)
Endotracheal intubation with mechanical ventilation	52 (19)
Comorbidities at the time of BAL, *n* (%)	
Engraftment syndrome	15 (5.4)
GVHD active at the time of BAL^m^	83/260 (31)
GVHD ever preceding BAL	126/260 (48.5)
Heart failure or reduced function	11 (4.0)
Kidney injury	47 (16.9)
Pericardial effusion	25 (9.0)
Pulmonary hemorrhage or hemoptysis	23 (8.3)
Sepsis	37 (13.3)
TA-TMA	22 (7.9)
VOD/SOS	24 (8.6)
Immunological function before BAL^n^	
WBC, median (IQR)	4,415 (2,370–8,400)
ANC, median (IQR)	3,060 (1,632–5,508)
ANC < 0.5 × 10^9^ l^−1^, *n* (%)	34 (12.2)
ALC, median (IQR)	420 (156–1,035)
ALC < 0.2 × 10^9^ l^−1^, *n* (%)	77 (27.7)
BAL clinical microbiology results, *n* (%)	
Any positive	116 (41.7)
Bacterial	51 (18.3)
Viral	76 (27.3)
Fungal and protozoal	25 (9.0)
More than one organism	29 (10.4)
Antimicrobials in the preceding week, median (IQR)	
Antibacterial	3 (2–4, range 0–9)
Antiviral	1 (1–2, range 0–3)
Antifungal	1 (0–1, range 0–3)
**Outcomes (*n*** = **229)**	
Required intensive care, *n* (%)	121 (52.8)
Required 7 or more days of mechanical ventilation, *n* (%)	71 (31.0)
In-hospital mortality, *n* (%)	45 (19.7)

^a^Includes B cell acute lymphoblastic leukemia (*n* = 54), acute myeloid leukemia (*n* = 39), T cell acute lymphoblastic leukemia (*n* = 12), juvenile myelomonocytic leukemia (*n* = 6), chronic myelogenous leukemia (*n* = 4) and other/myelodysplastic syndromes/mixed phenotype (*n* = 10).

^b^Includes severe combined immunodeficiency (*n* = 14), hemophagocytic lymphohistiocytosis (*n* = 7), chronic granulomatous disease (*n* = 4), Wiskott–Aldrich syndrome (*n* = 3) and other (*n* = 12).

^c^Includes severe aplastic anemia (*n* = 12), Fanconi anemia (*n* = 4), sickle cell disease (*n* = 9) and thalassemia (*n* = 2).

^d^Includes neuroblastoma (*n* = 10), medulloblastoma (*n* = 3) and other solid tumor (*n* = 1).

^e^Includes B cell lymphoma (*n* = 6), non-Epstein–Barr virus T cell lymphoma (*n* = 4) and Epstein–Barr virus + T cell lymphoma (*n* = 2).

^f^Includes Hurler syndrome (*n* = 4), osteopetrosis (*n* = 2), X-linked adrenoleukodystrophy (*n* = 2) and other (*n* = 3).

^g^Patients may have received multiple agents in the same or multiple categories.

^h^Includes carmustine (*n* = 2), treosulfan (*n* = 3), carboplatin (*n* = 4) and etoposide (*n* = 16).

^i^Missing in *n* = 14.

^j^Twenty-nine patients without clinical symptoms underwent BAL to evaluate declining PFTs or chest computed tomography (CT) abnormalities.

^k,l^Chest X-ray and chest CT obtained before *n* = 207 and *n* = 218 BALs, respectively.

^m^GVHD assessed in allograft recipients only.

^n^WBC, ANC and ALC expressed as 10^9^ cells per liter of whole blood.

BiPAP, biphasic positive airway pressure; CPAP, continuous positive airway pressure; PFT, pulmonary function test; SOS, sinusoidal obstruction syndrome; TA-TMA, transplant-associated thrombotic microangiopathy; UCB, umbilical cord blood; VOD, veno-occlusive disease; WBC, white blood cell count.

### Cluster derivation

BAL underwent bulk RNA sequencing (RNA-seq) followed by parallel alignment to microbial and human reference genomes (Fig. 1c and Methods). Microbial alignments were transformed from counts to quantitative masses using a reference spike-in, followed by stringent contamination subtraction. They were summarized according to taxa, Kyoto Encyclopedia of Genes and Genomes (KEGG) functional orthologs, richness and diversity. Human alignments were characterized according to normalized gene expression, pathway analysis, cell-type deconvolution and T and B cell receptor (BCR) alignments (Methods). To identify the underlying BAL subtypes with shared microbial–human metatranscriptomic composition, we used a two-step unsupervised approach consisting of (1) multi-factor dimensionality reduction (multi-omics factor analysis (MOFA)), followed by (2) uniform manifold approximation and projection (UMAP) with hierarchical clustering (Methods). Optimal fit statistics (Supplementary Fig. 1) suggested that four clusters best fitted the data (Fig. 1d).

### Clinical traits, illness severity and outcomes

Clinical data were analyzed after cluster assignment and revealed similar demographics, underlying disease and transplant regimens across clusters, with varying geographical regions and more females in clusters 3 and 4 (Supplementary Table 1). Patients in clusters 3 and 4 were generally sicker, as evidenced by greater need for respiratory support before BAL (*P* = 0.004), higher rates of renal injury and GVHD (*P* = 0.001 and *P* = 0.019), and greater use of intensive care (*P* = 0.001) or prolonged mechanical ventilation (≥7 days) after BAL (*P* = 0.001; Supplementary Table 2). Patients in clusters 3 and 4 also had significantly higher in-hospital mortality than patients in cluster 1 or 2 (log-rank *P* = 0.005; Fig. 1e). Among patients requiring respiratory support before BAL (44%), cluster-based mortality differences were pronounced and ranged from 22% to 30% in clusters 1 and 2 to 50–60% in clusters 3 and 4 (log-rank *P* = 0.007). Findings were similar when analyzing only patients enrolled within 100 days after HCT (Supplementary Table 3) and in a multivariable Cox regression model accounting for age, biological sex, absolute neutrophil count (ANC), ALC and presence of GVHD (*P* = 0.023; Supplementary Table 4).

### Microbial taxonomy

**Fig. 2:**
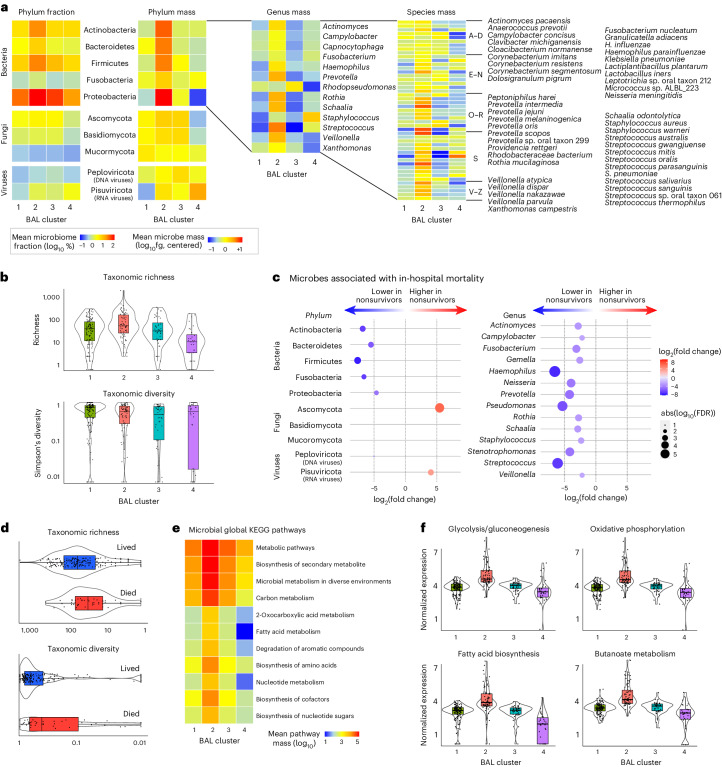
The BAL microbiome. **a**, The fraction (left) and mass (right) of major bacterial, viral and fungal phyla were plotted, with the shading representing the average for each of the four BAL clusters (*n* = 127, 74, 45 and 32 for clusters 1–4, respectively). The average mass of bacterial genera and species in each of the four BAL clusters are shown on the right. **b**, Taxonomic richness and diversity were plotted across the four BAL clusters. Richness and diversity varied across clusters (Kruskal–Wallis test, *P* < 0.001 and *P* = 0.002, respectively). **c**, Microorganisms associated with in-hospital mortality were identified using negative binomial generalized linear models (edgeR R package) and were plotted according to the log fold change (position, color) and FDR (dot size). **d**, Taxonomic richness and Simpson’s alpha diversity stratified according to survival status at the time of the most recent BAL (*n* = 184 survivors, *n* = 45 nonsurvivors). Richness and diversity differed according to survival outcome (Wilcoxon rank-sum test, *P* = 0.025 and *P* = 0.006, respectively). **e**, Microbial alignments to the KEGG metabolic pathways were averaged for each BAL cluster. **f**, Selected metabolic pathways that differed across the BAL clusters are shown. log_10_-normalized expression varied across clusters (Kruskal–Wallis test, FDR < 0.001 for each of glycolysis/gluconeogenesis, oxidative phosphorylation, fatty acid biosynthesis and butanoate metabolism). For all box plots: the boxes indicate the median and IQR; the whiskers extend to the largest value above the 75th percentile (or smallest value below the 25th percentile), that is, within 1.5 times the IQR.

To determine how microbiome composition drove differences between the clusters, we compared taxonomic mass, richness and diversity. Cluster 1 showed moderate microbiome mass and richness, high microbial diversity and a low burden of viruses. In contrast, cluster 2 showed high mass of bacterial phyla, high taxonomic richness and moderate microbial diversity (Fig. 2a,b and Supplementary Data 1). Cluster 3 demonstrated a reduced quantity and diversity of typically oropharyngeal microorganisms, with greater quantity of RNA viruses and the Ascomycota phylum of fungi, which contains medically relevant pathogens such as *Aspergillus*, *Candida* and *Pneumocystis*. In contrast, cluster 4 showed significant depletion of typical microbiome constituents, with minimal diversity and richness and concomitant enrichment of *Staphylococcus* and the Pisuviricota phylum of RNA viruses, which contains many respiratory RNA viruses, such as rhinovirus. When analyzed according to survivor status, nonsurvivors showed broad depletion of commensal taxa, higher quantities of fungal and viral RNA (Fig. 2c and Supplementary Data 2) and decreased BAL richness (*P* = 0.025) and diversity (Simpson’s diversity *P* = 0.006; Fig. 2d), which is consistent with the description of clusters 3 and 4. In contrast, survivors showed replete and bacterially diverse pulmonary microbiomes, consistent with the description of cluster 1.

### Microbial function

**Extended Data Fig. 1:**
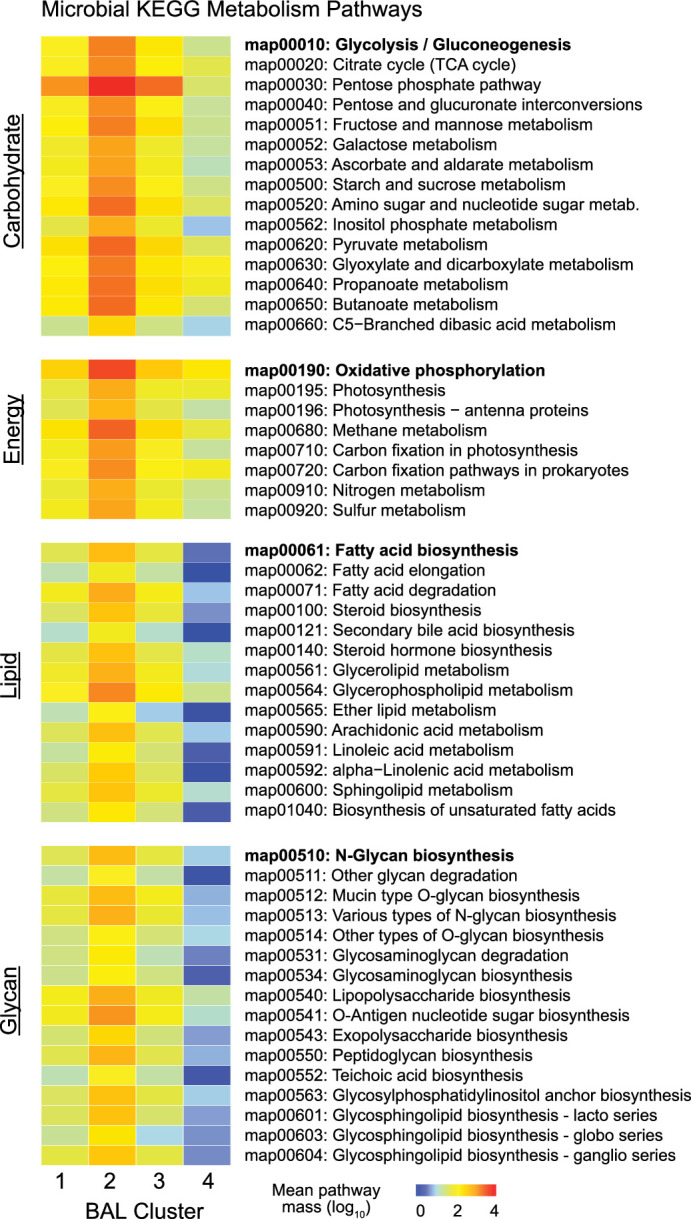
Microbial KEGG Metabolism Pathways. Mean ERCC-transformed normalized KEGG pathway expression for microbial Carbohydrate, Energy, Lipid, and Glycan metabolism pathways.

**Extended Data Fig. 2:**
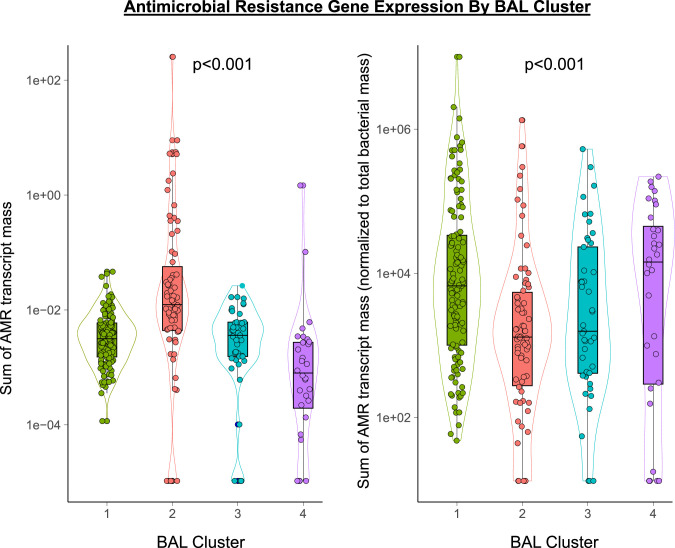
Antimicrobial Resistance Gene Expression By BAL Cluster. Antimicrobial resistance gene (AMR) expression was derived from human-subtracted sequencing files processed using the. CZID Antimicrobial Resistance (AMR) Gene Pipeline v0.2.4-beta, which leverages the Resistance Gene Identifier (RGI) v6.0.0 to generate read k-mer alignments (KMA) against the Comprehensive Antibiotic Resistance Database (CARD) v3.2.3 and WILDCARD v3.1.0. AMR transcripts were summed across all AMR genes and normalized to sample ERCC reads (left) and additionally to total BAL mass of bacteria (right) and varied by cluster (Kruskal-Wallis p < 0.001 and p < 0.001, respectively). n = 127, 74, 45, and 32 for Clusters 1-4, respectively. For all box-whisker plots: boxes indicate the median and interquartile range and whiskers extend to the largest value above the 75th percentile (or smallest value below the 25th percentile) that is within 1.5 times the IQR.

Transcriptomic markers of metabolic activity of microbial communities may complement taxonomic composition^14^. Using KEGG functional annotations, cluster 1 showed moderate transcription of myriad microbial metabolic functions across the domains of carbohydrate, lipid and fatty acid, and amino acid metabolism (Fig. 2e,f, Extended Data Fig. 1 and Supplementary Data 3). In contrast, the bacterially rich cluster 2 showed greater transcription of these domains and of glycan biosynthesis pathways, including peptidoglycan, lipopolysaccharide and other glycans that form bacterial cell walls. Cluster 3 showed significantly lower microbial function across the spectrum of the KEGG pathways; consistent with a depleted microbiome, cluster 4 showed minimal microbial metabolic activity. Antimicrobial resistance (AMR) gene expression was highest in the bacterially rich cluster 2 and lowest in the bacterially depleted cluster 4. However, AMR expression normalized to the quantity of BAL bacteria was lower for cluster 2 and highest in cluster 4, suggesting a shifting of bacterial metabolic function (Extended Data Fig. 2).

### Pathogen identification

Patients in this cohort had a wide range of distinct infections, thus lending unique elements to each microbiome. Therefore, we next compared the pathogenic microorganisms detected by hospital tests and sequencing (Supplementary Table 5 and Supplementary Data 4).

#### Viruses

**Fig. 3:**
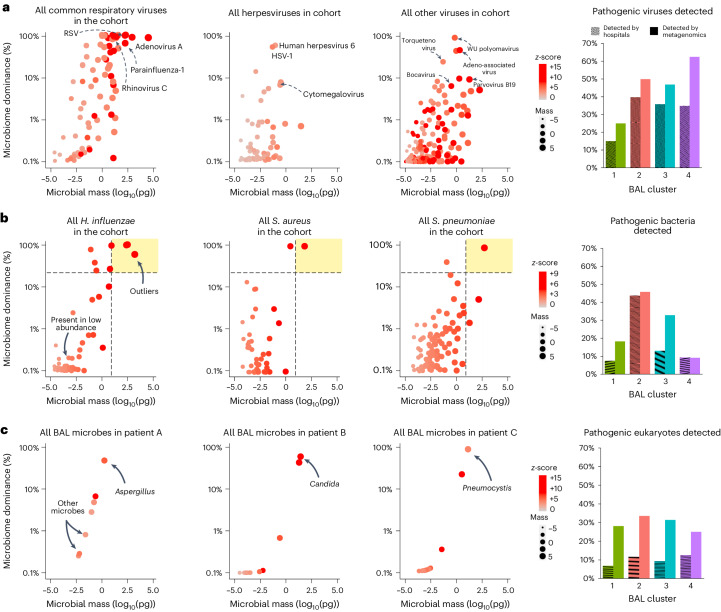
BAL pathogen detection. **a**, Left: dot plots of common community-transmitted respiratory viruses (left), herpesviruses (middle) and all other viruses (right) detected in the cohort, plotted according to microbial mass (*x* axis) and microbiome dominance (*y* axis). Right: bar chart comparing viral detection across the four BAL clusters according to hospital tests and metagenomic sequencing. **b**, Left: all *H. influenzae*, *S. aureus* and *S. pneumoniae* detected in the cohort were plotted, with the dashed lines indicating the cutoffs of mass ≥10 pg and bacterial dominance ≥20%. Taxa above these cutoffs are shown in the upper-right quadrant (shaded in yellow) to indicate outliers within the cohort. Right: bar chart comparing potentially pathogenic bacteria detected across the four BAL clusters according to hospital tests and metagenomic sequencing. **c**, Left: all microorganisms detected in the BAL of three patients are shown, with the arrows indicating fungi present in high quantities. Right: bar chart comparing potentially pathogenic eukaryotes detected across the four BAL clusters according to hospital tests and metagenomic sequencing.

Clinically, most community-acquired respiratory viruses (CRVs) are detected with multiplex PCR and reported as present or absent. Clinical testing found CRVs in 49 samples (18%), whereas sequencing identified CRVs in 77 samples (28%), highest in clusters 2, 3 and 4 (Fig. 3a). In addition to common CRVs, several variant strain CRVs, such as influenza C virus and rhinovirus C, were detected (GenBank: OQ116581, OQ116582, OQ116583). Clinical testing found herpesviruses, including cytomegalovirus and human herpesvirus 6 in 35 samples (13%), whereas sequencing found herpesviruses in 49 samples (16%), with the greatest detection in clusters 3 and 4 (Dunn’s test *P* = 0.018 and *P* = 0.021 for clusters 3 and 4 relative to cluster 1). Sequencing also detected many viruses known to have respiratory transmission but not typically included on respiratory viral panels, including BK, WU and KI polyomaviruses, bocavirus, parvovirus B19, lymphocytic choriomeningitis virus and non-vaccine strain rubella across 26 BALs from 23 patients. These viruses were most common in clusters 3 and 4 and associated with 39% in-hospital mortality (*n* = 9 of 23). The ubiquitous bystander torquetenovirus and its variants were detected in 55 samples (20%), again higher in clusters 2, 3 and 4 relative to cluster 1 (Supplementary Table 6; *P* < 0.001).

#### Bacteria

Clinically, most pathogenic respiratory bacteria are detected with selective culture media (blood, chocolate and MacConkey agar) optimized to grow certain pathogens above nonpathogenic flora, although PCR, serology and antigen tests may be used for certain organisms. In this study, clinical testing identified pathogenic bacteria in 51 samples, which were heavily overrepresented in the microbially rich cluster 2 (32 of 51 bacterial infections). In contrast, metagenomic sequencing is agnostic to organism pathogenicity and thus detects microorganisms broadly. As contamination is ubiquitous in low-biomass samples^15^, we used a strict approach to adjust for background taxa using internal spike-ins and batch-specific external controls (Methods). Still, many potentially pathogenic microorganisms were detected broadly; for example, *Streptococcus pneumoniae*, *Moraxella catarrhalis*, *Haemophilus influenzae*, *Staphylococcus aureus* and *Pseudomonas aeruginosa* were detected in 34%, 21%, 21%, 16% and 14% of samples (94, 58, 57, 44 and 39 samples), respectively. As some microorganisms could be present as commensals or pathogens depending on context and microbial burden, we then ranked bacteria according to RNA mass, dominance of the bacterial microbiome and intracohort *z*-score to parse the microorganisms most likely to be present in states of dysbiosis and thus potential infection (Fig. 3b). Using a conservative threshold of RNA mass of 10 pg or greater, bacterial dominance of 20% or greater and *z*-score of +2 or higher, we found potentially pathogenic bacteria in 76 samples, again with nearly half of these in cluster 2. In addition to new cases of common pathogens (for example, *P. aeruginosa*), many previously occult pathogens were identified above these thresholds, including *Bacillus cereus*, *Citrobacter freundii*, *Chlamydia pneumoniae*, *Klebsiella aerogenes*, *Salmonella enterica* and *Ureaplasma parvum*.

#### Eukaryotes

Using clinical assays, potentially pathogenic fungi were detected in 9% of samples (*n* = 25). As with bacteria, sequencing detected many potentially pathogenic fungi broadly in this cohort, for example, *Candida*, *Aspergillus*, *Fusarium* and *Rhizopus* were detected in 18%, 16%, 9% and 5% of samples (50, 44, 25 and 13), respectively. Applying a threshold of mass of 10 pg or greater and *z*-score of +2 or higher, potentially pathogenic fungi were detected in 30% of samples (83), with high detection across clusters 2, 3 and 4 (Fig. 3c). Several relevant fungi were detected exclusively using metagenomic sequencing, including *Cryptococcus* and *Pneumocystis*. No BAL parasites were detected through clinical assays, whereas metagenomic sequencing detected *Toxoplasma* in four patients and *Acanthamoeba* in three patients, with predominance in clusters 3 and 4 (Supplementary Data 4) and more than 50% mortality rate (*n* = 4/7).

**Extended Data Fig. 3:**
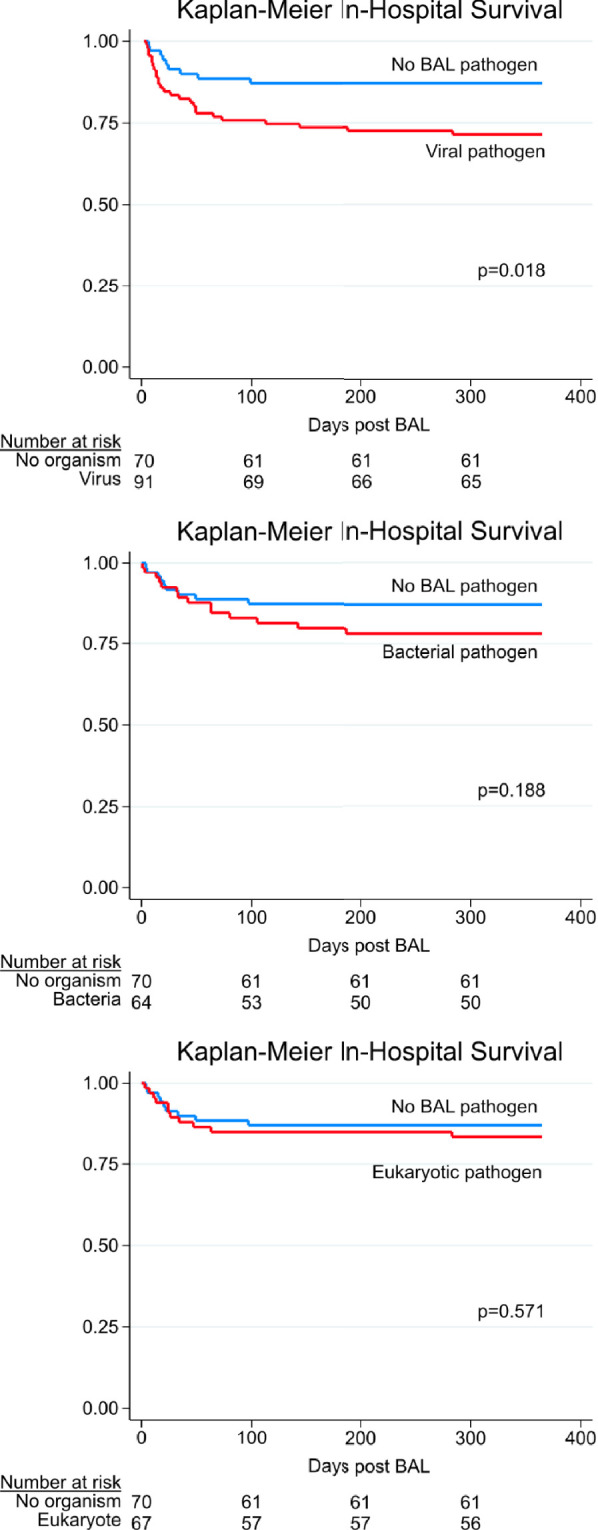
In-Hospital Survival Stratified by BAL Pathogen Detected. In-hospital survival for patients with a viral pathogen (top), bacterial pathogen (middle), or eukaryotic pathogen (bottom) detected on BAL, relative to no pathogen detected on BAL. Survival curves were compared using the two-sided log-rank test.

Overall, clinical testing identified 173 pathogens in 116 of 278 samples (41.7%), while metagenomic sequencing using conservative thresholds identified 360 pathogens in 196 of 278 samples (70.5%, McNemar’s *P* < 0.001; Supplementary Table 7). Combined clinical testing and metagenomic sequencing identified 429 pathogens in 209 of 278 samples (75.2%; Supplementary Table 5). A total of 90 cases of idiopathic pneumonia syndrome were reclassified as lower respiratory tract infection. Whereas clinical testing identified pathogens in 22 of 45 nonsurvivors (49%), sequencing identified credible pathogens in 36 of 45 nonsurvivors (80%, *P* = 0.002; Supplementary Table 8). In-hospital mortality was highest for those with a pathogen detected by both clinical testing and metagenomics, and lower if a pathogen was detected by metagenomics alone or was not detected at all (27% versus 19% versus 13%; Supplementary Table 9 and Extended Data Fig. 3).

### Impact of antimicrobial exposure

**Fig. 4:**
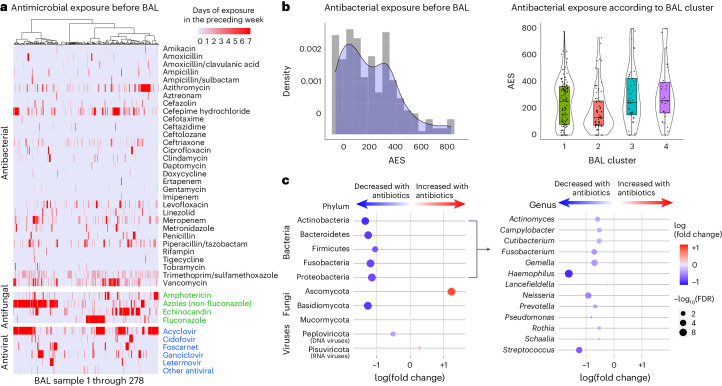
Antibiotic exposure and impact on BAL microbiome. **a**, Days of antimicrobial exposure are listed for antibacterials (black), antifungals (green) and antivirals (blue). Patients are listed in the columns and the shading indicates the number of days of exposure to each antibiotic in the week preceding BAL. **b**, AES was calculated before each BAL as the sum of antibiotic exposure days × a broadness weighting factor, summed for all therapies received in the week preceding BAL. AES varied across the clusters (*n* = 127, 74, 45 and 32 for clusters 1–4, respectively) and was highest for patients in cluster 4 (Kruskal–Wallis test, *P* = 0.005). For all box plots: the boxes indicate the median and IQR; the whiskers extend to the largest value above the 75th percentile (or the smallest value below the 25th percentile), that is, within 1.5 times the IQR. **c**, Negative binomial generalized linear models were used to test for BAL microorganisms associated with AES. Microorganisms are listed in the rows, with phyla shown on the left and bacterial genera shown on the right.

**Extended Data Fig. 4:**
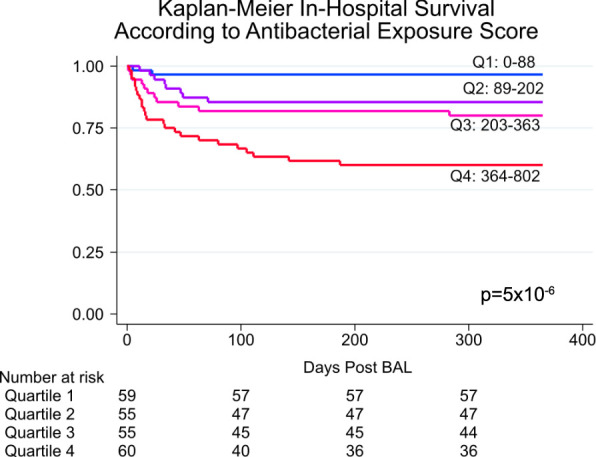
In-Hospital Survival Stratified by Antibacterial Exposure Score. Antibacterial exposure score (AES) was divided into 4 quartiles of equal patient number and in-hospital survival was plotted for each quartile and compared with the two-sided log-rank test.

To investigate the impact of antimicrobial exposure on BAL microbiomes, we quantified patient-level antibacterial exposure in the week preceding BAL by weighting the cumulative antibiotic exposure days with an agent-specific broadness score to yield an antibiotic exposure score (AES) (Fig. 4a,b and Methods). AES varied across clusters (*P* = 0.005) and was lowest for the microbially rich cluster 2 and highest for the microbially depleted clusters 3 and 4. Greater AES was associated with reduced BAL microbial richness (Spearman rho = −0.14, *P* = 0.018); depletion of all the major bacterial phyla, including many oropharyngeal-resident taxa; and enrichment of the fungal phylum Ascomycota (false discovery rate (FDR) < 0.05; Fig. 4c and Supplementary Data 5). Consistent with expected bacterial depletion, greater preceding AES was associated with lower BAL expression of AMR genes (Poisson regression *P* < 0.001); however, higher preceding AES was associated with greater BAL expression of AMR genes when normalized to total BAL bacterial mass (Poisson regression *P* < 0.001). In addition, AES was significantly greater among nonsurvivors (median = 352, IQR = 210–507 versus 175, IQR = 75–336, Wilcoxon rank-sum test *P* < 0.001; Extended Data Fig. 4). Using causal mediation analysis based on linear structural equation modeling (Methods), the association between greater AES and mortality was statistically mediated by an antibiotic-induced depletion of key commensal pulmonary bacteria including *Actinomyces*, *Fusobacterium*, *Gemella*, *Haemophilus*, *Neisseria*, *Rothia*, *Schaalia* and *Streptococcus* (*P* < 0.001; Supplementary Data 6). However, evidence for mediation was significantly diminished after adjusting models for preceding oxygen support, ANC and ALC (Supplementary Data 7). Similar to above, anti-anaerobic exposure was higher in nonsurvivors (*P* = 0.011) and was associated with BAL depletion of many anaerobes including *Prevotella*, *Gemella* and *Fusobacterium* (Supplementary Data 8). Antifungal exposure was higher in the microbially depleted cluster 4, driven largely by higher exposure to echinocandins (*P* = 0.019); antiviral exposure was higher in clusters 3 and 4, driven largely by higher exposure to cidofovir (*P* = 0.045).

### Impact of clinical immune status

The pulmonary microbiome exists in a state of reciprocal interaction with the lung epithelium, stroma and immune system. Analysis of patient immune laboratory tests showed that ANC was highest in the bacterially rich cluster 2 (*P* = 0.029; Supplementary Table 2) but was not associated with mortality overall (*P* = 0.810). In contrast, ALC did not vary across clusters (*P* = 0.997) but was lower in nonsurvivors (median = 273 cells per microliter, IQR = 125–650 versus 422, IQR = 179–1120, *P* = 0.028).

### Pulmonary gene expression

**Fig. 5:**
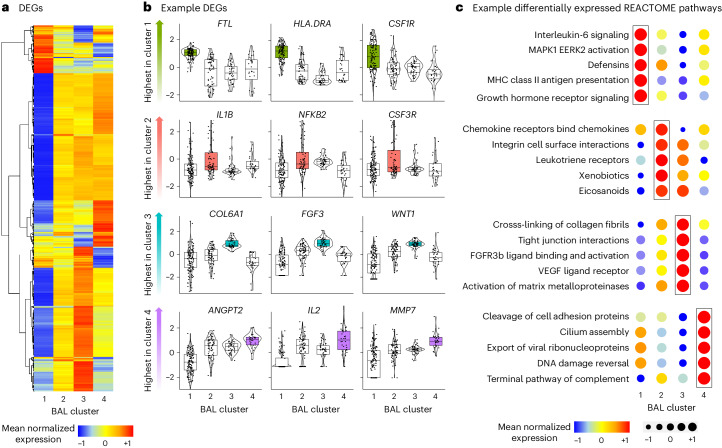
BAL gene expression. **a**, DEGs were identified using a four-way analysis of variance-like analysis with negative binomial generalized linear models. Mean normalized expression levels for significant genes are displayed for the four BAL clusters. **b**, Individual DEGs were identified across the four clusters (edgeR R package); variance-stabilized transformed gene counts for select genes highest in each of the four clusters were plotted (*n* = 127, 74, 45 and 32 for clusters 1–4, respectively). For all box plots: boxes indicate the median and IQR; the whiskers extend to the largest value above the 75th percentile (or smallest value below the 25th percentile), that is, within 1.5 times the IQR. **c**, Gene set enrichment scores to REACTOME pathways were calculated and example gene sets most enriched in each of the four clusters are shown.

We then compared BAL human gene expression across the four clusters and identified 18,158 differentially expressed genes (DEGs) (Fig. 5a and Supplementary Data 9). Select genes most differentially expressed in each cluster are displayed in Fig. 5b. Using REACTOME gene set enrichment scores (Supplementary Data 10), we showed that clusters were differentiated by high expression of pathways related to antigen-presenting cell activation (cluster 1); neutrophil and innate immune activation, bacterial processing and airway inflammation (cluster 2); collagen deposition and fibroproliferation (cluster 3); and antiviral and cellular injury genes (cluster 4; Fig. 5c). To replicate these findings orthogonally, we identified 1,253 genes differentially expressed between survivors and nonsurvivors (Supplementary Data 11). Consistent with the description of clusters 3 and 4, nonsurvivors showed broad downregulation of innate immune and antigen-presenting signals and a significant upregulation in collagen deposition, matrix metalloproteinases, alveolar epithelial hyperplasia and fibroproliferative genes (for example, *COL1A1*, *COL3A1*, *CXCL5*, *IL13*, *MMP7*, *SFTPA1*, *SFTPC* and *TIMP3*, each detected at an FDR < 0.05).

### BAL cell-type imputation

**Extended Data Fig. 5:**
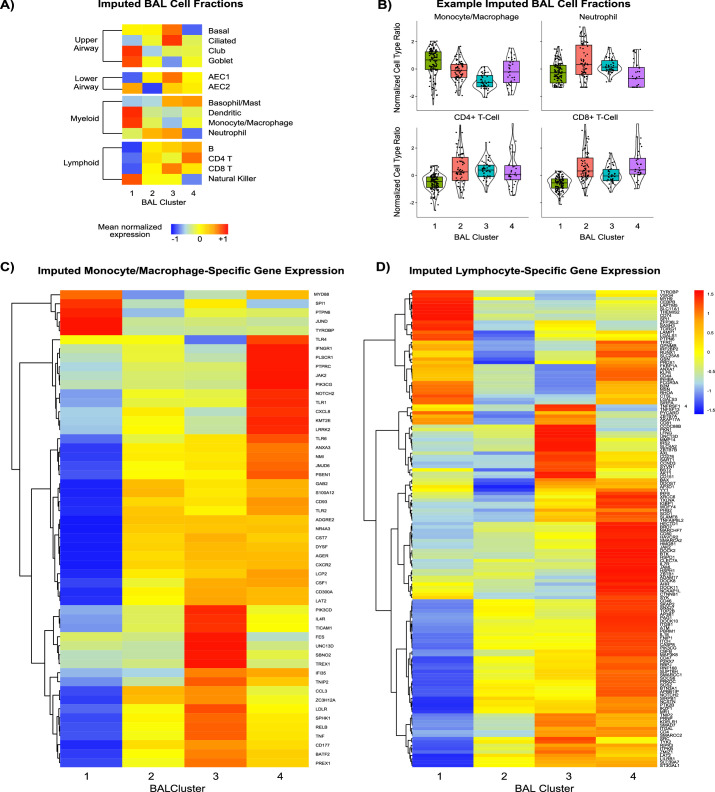
BAL Cell-Type Deconvolution and Imputed Cell-Specific Gene Expression. Cell type fractions were imputed using bulk gene expression and a reference single cell lung atlas. (**a**) Mean centered, scaled fractions are depicted for each cluster and varied across clusters for Monocyte/macrophages, neutrophils, CD4 + T-cells, and CD8+ Tcells (Kruskal-Wallis p = 4.14 × 10-24, 3.81×10-5, 1.89×10-22, and 3.36×10-13, respectively). (**b**) Raw values are shown for specific cell types. (**c**) Monocyte/macrophage-specific expression of the ‘GOBP Myeloid Leukocyte Activation’ gene set was imputed. Genes that were statistically significantly differentially expressed across clusters were selected for the heatmap, and average cell-type specific gene expression across the 4 clusters is displayed. (**d**) Lymphocyte-specific expression of ‘GOBP Lymphocyte Activation’ gene set was imputed. Genes that were statistically significantly differentially expressed across clusters were selected for the heatmap, and average cell-type specific gene expression across the 4 clusters is displayed. n = 127, 74, 45, and 32 for Clusters 1-4, respectively. For all box-whisker plots: boxes indicate the median and interquartile range and whiskers extend to the largest value above the 75th percentile (or smallest value below the 25th percentile) that is within 1.5 times the IQR.

**Extended Data Fig. 6:**
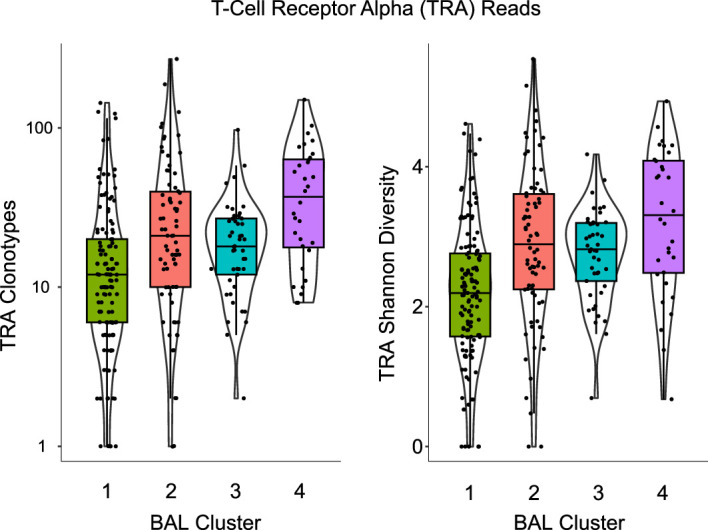
BAL T-Cell Receptor Repertoires. CDR3 alignments were computed and clonotypes and Shannon diversity of TCRα alignments are shown for each of the BAL clusters. n = 127, 74, 45, and 32 for Clusters 1-4, respectively. TRA clonotypes and shannon diversity varied by cluster (Kruskal-Wallis p = 1.32 × 10-7 and 3.27 × 10-8, respectively). For all box-whisker plots: boxes indicate the median and interquartile range and whiskers extend to the largest value above the 75th percentile (or smallest value below the 25th percentile) that is within 1.5 times the IQR.

BAL contains an admixture of cell types in contact with the lumen of the lower respiratory tract; thus, varying cell proportions or activity levels may account for differential gene expression detected by bulk sequencing. Using cell-type deconvolution, we showed that clusters were differentiated by high fractions of monocytes and macrophages (cluster 1), neutrophils (cluster 2), CD4^+^ T cells (cluster 3) and CD8^+^ T cells (cluster 4) (Methods and Extended Data Fig. 5a,b). To assess differences in cell-type-specific gene expression, we next imputed monocyte-specific expression of the Gene Ontology Biological Process (GOBP) ‘Myeloid Leukocyte Activation’ gene set (including *CSF1*, *IFNGR1*, *LDLR*, *TLR1* and *TNF*) and found the highest expression in clusters 2, 3 and 4 (Methods and Extended Data Fig. 5c). Although cluster 1 had a high monocyte and macrophage cell fraction, lineage-specific inflammatory gene activation was relatively low in this cluster. Similarly, lymphocyte-specific expression of the GOBP ‘Lymphocyte Activation’ gene set (including *AKT1*, *BTK*, *CD4*, *DOCK8*, *JAK2* and *IL7R*) was highest in clusters 3 and 4 (Extended Data Fig. 5d). We then used ImReP to measure lymphocyte receptor repertoires across the clusters, which showed that most CDR3 alignments were for T cell receptor-α (TCRα), with many fewer alignments to β, γ and δ and BCR H, K, or L. Whereas the virally enriched cluster 4 showed the highest number of TCRα clonotypes and diversity, cluster 1 showed the lowest (Extended Data Fig. 6). Notably, BAL TCRαβ clonotype numbers and diversity were not correlated with blood ALC (*P* = 0.646), although BAL TCRγδ and BCR subtypes were higher in patients with higher blood ALC (*P* = 0.041 and *P* = 0.006, respectively).

### Cluster transitions

**Extended Data Fig. 7:**
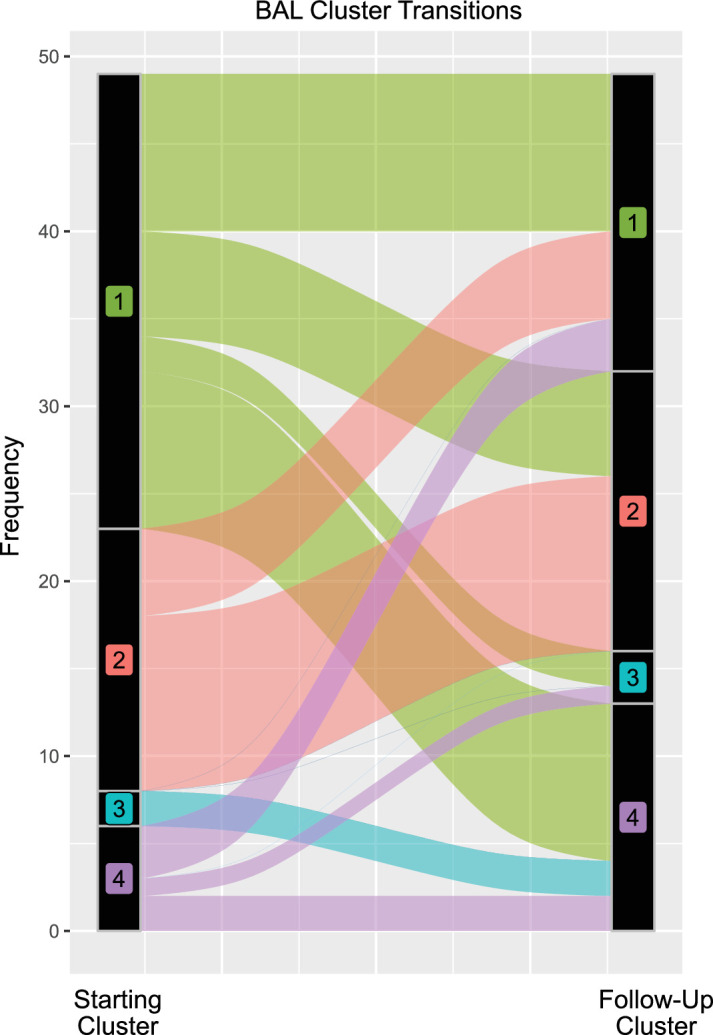
BAL Cluster Transitions. 34 patients had ≥2 BALs in this study (total 49 BALs were repeat samples). Cluster transitions are shown here, indicating a general transition away from the low-risk Cluster 1 on repeat samples.

We next assessed whether original cluster assignments were stable over time. After the first BAL, 34 patients underwent an additional 1 or more BALs separated by a median of 79 days (IQR = 21–243) due to worsening lung disease or concern for a new pulmonary process. Most patients who started in the low-risk cluster 1 moved out of cluster 1 (17 of 26) to a higher-risk cluster; patients who started outside cluster 1 rarely moved into cluster 1 (8 of 49), driving an overall change in the cluster burden over time (*P* < 0.001; Extended Data Fig. 7 and Supplementary Tables 10 and 11). This suggests that, for patients with recurrent or non-resolving symptoms, progression to an adverse BAL phenotype is common.

### Classification model and external cluster validation

**Extended Data Fig. 8:**
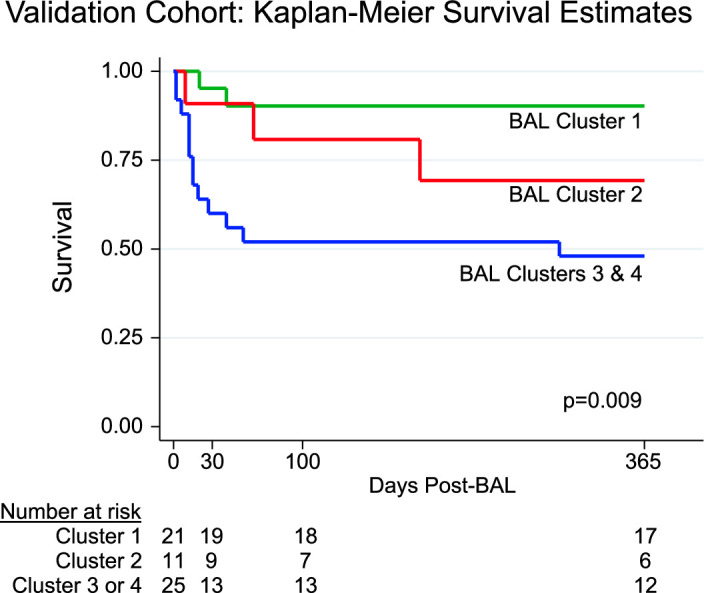
Validation Cohort Survival Stratified by BAL Cluster. A random forest classifier using BAL metagenomic and transcriptomic data was grown using the derivation validation set. The classifier was applied to BAL data from a validation cohort, and 1-year non-relapse mortality was plotted according to cluster assignment and compared using the two-sided log-rank test.

Finally, as cluster assignments cannot be directly applied to external cohorts, we used taxonomic and gene expression data to grow a random forest of 10,000 trees to be used as a cluster classifier. The out-of-bag area under the curve (AUC) was 0.923, indicating good cluster discrimination (Supplementary Table 12). Lung gene expression variables were significantly more important to cluster classification than taxonomic variables, with the 500 most important genes showing significant enrichment for immune processes (Supplementary Data 12). The random forest classifier was then applied to taxonomic and gene expression data from an independent cohort of *n* = 57 BALs obtained from pediatric recipients of HCT at the University Medical Center in Utrecht, the Netherlands, between 2005 and 2016 (clinical traits are described in Supplementary Table 13). Although this cohort differed in geography, underlying diseases, allograft characteristics and treatment protocols, 1-year non-relapse mortality was lowest among patients with BALs assigned to the low-risk cluster 1 (9.5%, 2 of 21), was higher for patients assigned to the bacterially rich cluster 2 (36%, 4 of 11), and was highest for patients in the high-risk clusters 3 or 4 (52%, 13 of 25, *P* = 0.009; Extended Data Fig. 8 and Supplementary Table 14), thus confirming the external validity and clinical significance of the BAL cluster profiles.

## Discussion

Lung injury in pediatric patients who have undergone HCT is frequently fatal, yet a lack of investigable biospecimens has hindered progress in elucidating disease pathobiology. In this prospective multicenter study, we used BAL from children at 32 hospitals to identify microbial dysbiosis, undetected infection and subtypes of inflammation and fibroproliferation as hallmarks of fatal disease. Our findings come from a broad, international cohort of children with poor immunity and high antimicrobial exposure and were replicated in an unrelated validation cohort. These findings extend our previous work in pediatric candidates for HCT and suggest the possibility for precision pulmonary phenotyping as a key step for future trials.

**Extended Data Fig. 9:**
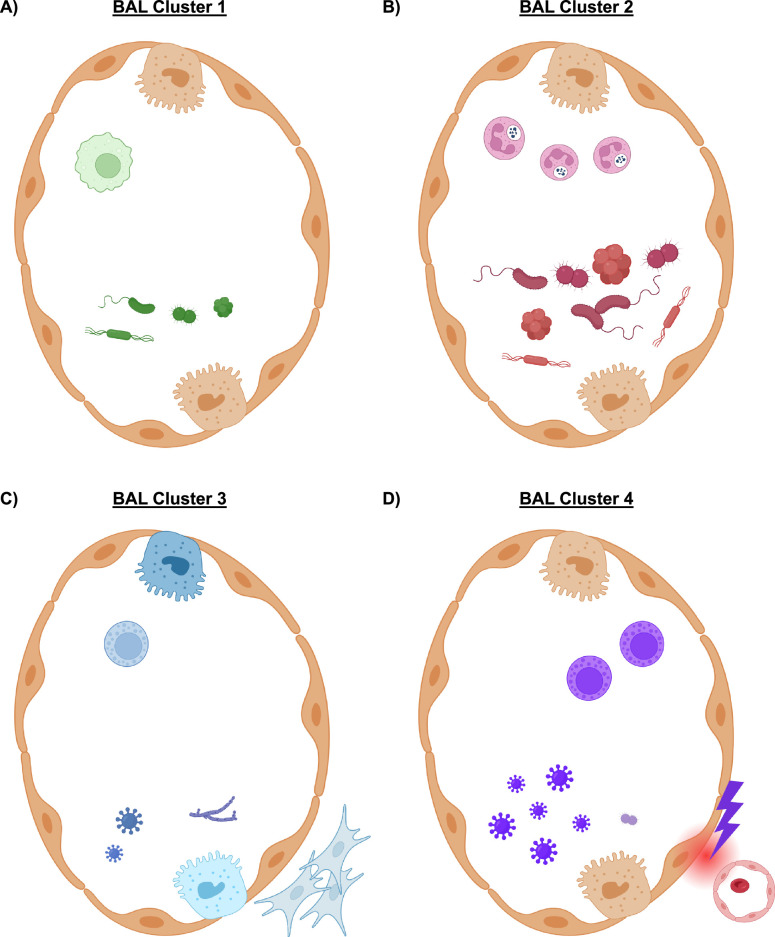
BAL Cluster Schema. (**a**) BAL Cluster 1 was most common, had moderate microbial burden, low rates of infection, predominantly alveolar macrophage-related signaling, and the lowest mortality rates. (**b**) Cluster 2 showed high rates of microbial burden and bacterial infections, higher neutrophil markers, and moderate mortality. (**c**) Cluster 3 showed microbiome depletion with enrichment of viruses and fungi and fibroproliferative gene expression. (**d**) Cluster 4 showed significant microbiome depletion with relative sparing of Staphylococci and enrichment of viruses, commensurate with lymphocytic inflammation, cellular injury, and the highest mortality rate.

A major finding of our work is the identification of biological subtypes where disease classification has been historically difficult^2^. BAL cluster 1 was most common, had moderate microbial burden, low rates of infection, predominantly alveolar macrophage-related signaling and the lowest mortality rates. In contrast, cluster 2 showed high rates of microbial burden and bacterial infections, higher neutrophil markers and moderate mortality. Cluster 3 showed microbiome depletion with enrichment of viruses and fungi and epithelial fibroproliferative gene expression. Cluster 4 showed significant microbiome depletion with relative sparing of staphylococci and enrichment of viruses, commensurate with lymphocytic inflammation, cellular injury and the highest mortality rate (summarized in Extended Data Fig. 9). In the field of pulmonology, subclasses of asthma, acute respiratory distress syndrome and chronic obstructive pulmonary disease (COPD) have recently been associated with distinct clinical trajectories such that subclass-specific clinical trials are now emerging^16–18^. The identification of heterogeneous clusters may be the first step in improving bedside phenotyping and ultimately enrolling pediatric patients who have undergone HCT in biology-targeted interventional trials.

A second major finding of our work is the illumination of the delicate balance between the pulmonary microbiome and mortality. The pulmonary microbiome is populated early in life by aerosolization of oropharyngeal microorganisms during tidal ventilation, gastric aspiration and disease-related hematogenous spread^7,9,19,20^. The near-continuous exposure of the lungs to microorganisms introduces the opportunity for infection but also supports immune and epithelial education in the form of tolerance and memory^21,22^. The ideal properties of the peri-HCT pulmonary microbiome probably require delicate balance between overpopulation and eradication^7,9^. Favoring the need to limit microbial overpopulation, studies in cystic fibrosis and COPD showed that an increase in pulmonary microbial mass is associated with neutrophilic inflammation and disease exacerbations^23–25^, a paradigm similar to patients in our bacterially enriched and neutrophil-enriched cluster 2. Favoring the latter, recent studies showed that patients who have undergone HCT with dysbiotic intestinal microbiomes develop higher mortality rates because of excess colitis, GVHD and pulmonary disease, which is similar to patients in our clusters 3 and 4 (refs. ^26–28^). Our data show that commensal biodiversity exists reciprocally with pathogenic taxa such as *S. aureus*, *P. aeruginosa*, fungi and viruses, suggesting that commensal constituents may limit the ability for pathogens to expand^29,30^, perhaps through local immunomodulation or by direct nutrient competition^24,31–34^. We showed that the transcriptional activity of BAL microorganisms is quite broad in patients with better clinical outcomes, raising the possibility that microbial metabolites might benefit airway health, as recently showed for the anti-apoptotic microbial metabolite indole-3-acetic acid^14,35,36^.

Antimicrobial exposure has been strongly associated with intestinal microbiome depletion and, to a lesser extent, pulmonary microbiome alterations mostly in the populations with cystic fibrosis and COPD^37–44^. While our data show that high AES is associated with microbiome depletion and in-hospital mortality, disentangling the relationship between antibiotic exposure, depleted microbiomes and poor clinical outcomes is difficult in an observational study, especially because sicker patients generally receive more antibiotics. Interestingly, we found that the quantity of the fungal phylum Ascomycota increased with greater AES, supporting existing evidence that depletion of commensal microorganisms may open a niche for opportunistic fungal growth^45–48^. Increased AES was associated with greater BAL quantity of respiratory RNA viruses, which is consistent with previous associations between antibiotic exposure and viral expansion^49,50^. Certainly for critically ill patients with unclear diagnoses, it will be difficult to feel confident in stopping antibiotics, although rapid turnaround of clinical metagenomics assays may facilitate this in some cases^51^. Ultimately, microbiome restorative therapies in patients necessarily antibiotic-exposed merits investigation in this population^52^.

Over the past 30 years, many studies have confirmed that metagenomic sequencing can increase diagnostic yield for pathogens^53–55^. However, application to respiratory fluid has been hindered by difficulty discriminating when a normal microbiome constituent such as *S. pneumoniae* expands to function as a pathogen. To address this, we transformed our sequencing data from fractional to absolute using reference spike-ins and then compared each microorganismʼs detected level to that of other microorganisms in the sample (dominance) as well as to other samples in the cohort (*z*-score). By parsing microorganisms in the context of the broader microbiome, we provide a logical and intuitive approach to pathogen detection in unsterile body sites. This approach nearly doubled the number of patients with detected infections, while also providing a safeguard against overcalling hits. Importantly, we identified new viral strains, common and rare bacteria, and many fungi and parasites as previously undetected causes of lung injury. Our data support the premise of a clinical trial using metagenomics to augment the utility of hospital diagnostics for patients who have undergone HCT, in which pathogen eradication, antibiotic de-escalation and avoidance of dysbiosis may be useful outcome metrics.

The relationship between the pulmonary microbiome, lung epithelium and the transplanted immune system is characterized by a continuous mutually influential interaction. In murine models of allogeneic HCT, immune responses to pathogens can be both impaired and exaggerated, leading to delayed phagocytosis, excessive myeloid cell recruitment and unremitting inflammation because of a lack of functional natural killer and T cells^56–59^. Our data support this paradigm and reveal a complex heterogeneous immune response. Cluster 1, with a replete and diverse pulmonary microbiome, showed the lowest mortality rates, low levels of granulocyte activation and low levels of lymphocyte diversity and lymphocyte-specific activation markers. In contrast, cluster 2 showed neutrophil enrichment, and clusters 3 and 4 showed a diverse lymphocyte population with markers of activation. Clinically, these distinctions may be important because patients might benefit from different approaches to immunomodulation. Notably, cluster 3 showed many markers of fibroproliferation and cellular senescence, suggesting transition to a fibrotic phenotype that may merit treatment in upcoming clinical trials using new antifibrotic agents^60^.

This study has several limitations. First, the cohort’s clinical heterogeneity requires interpreting the findings broadly. Second, clinical protocols were not standardized and post-HCT care varied across centers. Third, BAL collection was not standardized across centers and bronchoscope controls were not obtained. Fourth, controls from healthy children were not available. Fifth, without detailed histopathology, we could not adjudicate the contribution of identified microorganisms to each patient’s pulmonary disease. Sixth, clinical microbiological testing of BAL varied across hospitals and was not standardized. Finally, as with all observational human studies, we cannot prove causal relationships between exposures, measurements and outcomes.

In summary, we present the largest investigation to date of the pulmonary microbiome and transcriptome in pediatric patients who have undergone HCT. We identified four unique BAL clusters, with the worst outcomes observed for those with commensal microorganism depletion, viral or fungal enrichment, lymphocyte activation and fibroproliferation. Overall, these findings represent a step forward in understanding lung disease biology in patients who have undergone HCT and may be used to guide a future biology-targeted clinical trial.

## Methods

### Ethics statement

Patients or their guardians were approached prospectively for written informed consent under local institutional review board (IRB) approval at each site (University of California, San Francisco (UCSF) IRB nos. 14-13546 and 16-18908; Utrecht IRB nos. 05/143 and 11/063) in accordance with the 2013 Declaration of Helsinki and permission was obtained to collect leftover BAL fluid.

### Patients

The derivation cohort was enrolled through the Pediatric Transplantation and Cell Therapy Consortium (PTCTC) (NCT02926612) and the validation cohort was collected at the University Medical Center in Utrecht, the Netherlands. Participating pediatric centers screened all patients with a history of allogeneic (both cohorts) or autologous (PTCTC cohort only) HCT preparing to undergo clinically indicated bronchoscopic BAL for diagnostic assessment of pulmonary disease. Patients were excluded if there was a limitation of care, such as do not resuscitate at the time of BAL.

### BAL specimen collection

Bronchoscopy and BAL were performed at the discretion of the treating team using local institutional protocols. All BAL samples were obtained by pediatric pulmonologists trained in fiberoptic bronchoscopy with anesthesia provided by anesthesiologists or critical care physicians. The lavage protocol was not dictated by the study but typically involved 3–6 aliquots of 10 ml sterile saline inserted into the diseased areas of the lung as determined by preceding chest imaging or physical examination. The percentage of lavage returned was not routinely documented and lavage aliquots were typically pooled by the clinical team immediately after collection. After aliquoting for clinical testing, excess lavage was placed immediately on dry ice, stored at −70 °C, shipped to UCSF and stored at −70 °C until processing.

### Clinical protocols and data collection

Clinical microbiological testing was determined by the treating team and typically included culture for bacteria, fungi and acid-fast bacillus; multiplex PCR for respiratory viruses; galactomannan antigen; and cytology for *Pneumocystis carinii* pneumonia. Additional molecular diagnostics, such as PCR for atypical bacteria or fungi, were used at the discretion of the site. After BAL, supportive care protocols were determined by the treating team; all patients were enrolled at centers with pediatric intensive care units. Patient demographics, medical history and transplant-specific data were documented by trained study coordinators at each site. The most recent ANC and ALC measured clinically before BAL were documented. The results of clinical microbiological testing on BAL were documented and not considered complete until 4 weeks after collection. For the PTCTC cohort, all doses of antimicrobials administered in the 7 days before BAL were documented. The AES was calculated by summing the days of exposure to each antibacterial agent weighted with an agent-specific broadness score ranging from 4 to 49.75 (for example, ampicillin 13.50, meropenem 41.50)^61^. Daily dosages were not collected. The number of anti-anaerobe days were calculated as the sum of the preceding exposure to each of the following: amoxicillin/clavulanic acid; ampicillin/sulbactam; piperacillin/tazobactam; meropenem; ertapenem; imipenem; levofloxacin; clindamycin; doxycycline; tigecycline; or metronidazole. Patients were followed until hospital discharge (PTCTC) or until at least 1 year after BAL (Utrecht), with no loss to follow-up.

### BAL RNA extraction

After collection across 32 centers in the PTCTC cohort and one center in the Utrecht cohort, all samples were shipped to and processed in one laboratory at UCSF; the PTCTC samples were processed and sequenced in four batches. Samples were used on the first or second thaw. All samples underwent a previously described RNA extraction protocol optimized for BAL fluid^8^. A total of 200 µl of BAL was combined with 200 µl DNA/RNA Shield (Zymo Research) and 0.5-mm glass bashing beads (Omni) for five cycles of 25-s bashing at 30 Hz, with 60 s of rest on ice between each cycle (TissueLyser II, QIAGEN). Subsequently, samples were centrifuged for 10 min at 4 °C and the supernatant was used for column-based RNA extraction with DNase treatment according to the manufacturer’s recommendations (ZR-Duet DNA/RNA MiniPrep Kit, Zymo Research). The resultant RNA was eluted in 5 µl sterile water and stored at −70 °C until sequencing library preparation.

### BAL RNA-seq

Samples underwent a previously described sequencing library preparation protocol optimized for BAL fluid^62^. First, BAL RNA was dehydrated at 40 °C for 25 min in a 384-well plate (Genevac EZ2). Second, sequencing libraries were prepared using miniaturized protocols adapted from the Ultra II RNA Library Prep Kit (New England Biolabs) (dx.doi.org/10.17504/protocols.io.tcaeise). Reagents were dispensed using the Echo 525 (Labcyte) and underwent Ampure-XP bead cleaning on a Biomek NX^P^ instrument (Beckman Coulter). Libraries underwent 19 cycles of PCR amplification, size selection to a target 300–700 nucleotides (nt) and were pooled to facilitate approximately even depth of sequencing. Twenty-five picograms of External RNA Controls Consortium (ERCC) pooled standards were spiked-in to each sample after RNA extraction and before library preparation to serve as internal positive controls (catalog no. 4456740, Thermo Fisher Scientific). In addition, to identify contamination in laboratory reagents and the laboratory environment, each batch contained two samples of 200 µl sterile water and 6–8 samples of 200 µl HeLa cells taken from a laboratory stock and processed identically to the patient samples to account for laboratory-introduced and reagent-introduced contamination. These samples were processed at the same time as the patient BAL samples using the same lot of reagents to minimize batch effect on control samples. Samples were pooled across lanes of an Illumina NovaSeq 6000 instrument and sequenced to a target depth of 40 million read pairs with sequencing read length of 125 nt.

### Sequencing file processing

#### Human alignments

Resultant FASTQ files underwent alignment to hg38 (STAR package), producing 60,590 total genes detected across all samples (median = 44,063, IQR = 31,553–52,129). Human reads occupied a mean 96.8% of all transcripts (s.d. = 6.1%, range = 52.6–99.9%). Mitochondrial, ribosomal and non-protein-coding transcripts were excluded, leading to the detection of 19,032 protein-coding genes (median 18,259 genes per sample, IQR = 16,988–18,871). Batch effect was tested by performing principal component analysis of normalized transcript counts (DESeq2, vst R packages) and overlaying extraction batch on a three-dimensional plot of the first three principal components; we did not detect sample clustering according to batch.

#### Microbial taxonomic alignment

Human-subtracted sequencing files were generated using the CZID pipeline v.7.1 (https://github.com/chanzuckerberg/czid-web)^63^. Briefly, FASTQ files underwent a first round of human read subtraction (STAR to hg38) followed by Illumina adapter removal (Trimmomatic), quality filtering (PriceSeq package) and Lempel–Ziv–Welch complexity filtering. Duplicate nonhuman reads were temporarily set aside to facilitate efficient microbial alignment (CD-HIT-DUP), Next, sequencing files underwent a second more stringent round of human read subtraction (Bowtie 2) followed by a third round of human read subtraction (STAR), subsampling to 1 million fragments, and a fourth and final round of human read subtraction (GSNAP). Human-subtracted files underwent alignment to the NCBI nt/nr database using GSNAP with a minimum alignment length greater than 36. Quality metrics for the sequencing run, including the percentage of reads that passed the PriceSeq filter step and the percentage of reads that passed all steps were examined and samples with poor sequencing quality were resequenced. Duplicate reads were added back in and taxa counts were generated with associated metrics of percentage identity, contig length and e-value to the nearest NCBI hit. To reduce spurious associations due to ambiguous alignments, taxa were excluded if they (1) aligned to archaea or uncultured microorganisms, (2) had 6 or fewer total reads, (3) had less than 100 nt alignment length, or (4) had less than 80%, 90% or 95% nucleotide percentage identity for viruses, eukaryotes and bacteria, respectively. In addition, samples with low biomass (less than 100 pg) were further filtered to keep only taxa with 10 or more transcripts forming a contig of 250 nt or more with 80% or more percentage identity to the nearest NCBI hit. After all filtering, high-quality microbial reads occupied a mean 1.6% of all reads (s.d. = 2.1%, range = 3 × 10^−5^–10.4%).

#### Microbial functional alignment

Human-subtracted sequencing files were processed using FMAP v.0.15 (ref. ^64^) to profile the metabolic pathways present in each sample. FMAP_mapping.pl paired with diamond v.0.9.24 (ref. ^65^) and FMAP_quantification.pl were used with default settings to identify and quantify associated proteins in the UniRef90 database^66,67^. Gene assignments were regrouped by KEGG descriptors^68^ and their annotation was summarized at levels 1–3. In addition, human-subtracted sequencing files were processed using the CZID AMR Gene Pipeline v.0.2.4-beta, which leverages the Resistance Gene Identifier v.6.0.0 to generate read *k*-mer alignments against the Comprehensive Antibiotic Resistance Database v.3.2.3 and WILDCARD v.3.1.0. AMR transcripts were removed if coverage breadth was less than 5% or if they were highly expressed in HeLa and water samples (TEM-116, TEM-70).

### Microbial quantification and contamination

Low-biomass samples are susceptible to contamination^15^. We previously showed that a positive control spike-in to each sample can be used to back-calculate the original RNA mass of the sample and its various components^69^. Using varying quantities of RNA input, we demonstrated a linear relationship between log_10_(input or mass) and log_10_(output or sequencing reads). Hence, the original RNA mass of each clinical sample can be back-calculated by solving the linear proportionality equation (total sample reads/total sample mass) ≈ (ERCC reads/ERCC mass), where sample reads and ERCC reads were detected using the above protocol and ERCC input was standardized as 25 pg (ref. ^69^). In this study, we verified this relationship (Supplementary Fig. 2a) and then calculated the mass of each sample according to the formula above, further reduced by 25 pg (the ERCC input) to equal the original sample mass before ERCC addition. As the input RNA mass of the water controls was determined to be about 5 pg, presumably reflecting 5 pg of sequenceable contamination, we discarded samples whose total input mass was below 10 pg, as we were unable to reliably differentiate between contamination and true constituents. As low-biomass samples will preferentially amplify contaminants, we then used the ERCC spike-in to transform reads into estimated mass, allowing the analysis of both fractional and absolute microbiome properties. As each BAL microbiome consists of contributions from the patient and externally introduced contaminants, we then calculated the unique contamination profile of the water and HeLa samples for each sequencing batch (Supplementary Fig. 2b and Supplementary Data 13 and 14), and subtracted the mean + 2 s.d. of each contaminant taxa from the patient samples processed in the respective batch. Mass-transformed and contamination-adjusted values were used for downstream analysis, including unsupervised clustering analysis.

### Statistical analysis

#### Unsupervised clustering analysis

As microbiome data can be described using taxonomy, functional annotation or summary measures, we used MOFA to reduce dimensionality and identify a core set of factors^70^. This approach accommodates different data structures and distributions and is tolerant of collinearity. Data were filtered to include phyla, genera, species and KEGG pathways present in more than 15% of samples, underwent variance stabilizing transformation (vst, DESeq2 R packages) and were combined with aggregate metrics of total microbial mass, Simpson’s and Shannon’s alpha diversity (vegan), and richness, which was defined as the number of species detected at a threshold of 1 pg or more^71,72^. MOFA was used to identify 15 core latent factors that together explained the most variance in the data structure. The matrix of latent factor values then underwent UMAP (umap R package) and BAL clusters were identified using hierarchical clustering of Euclidean distances (eclust, factoextra R packages). The ideal number of clusters was determined to be four using silhouette, elbow and gap statistic plots. Sample processing batches were overlaid on clusters to confirm lack of batch effect.

#### Clinical characteristics

Kaplan–Meier survival analysis was used to plot in-hospital mortality according to BAL cluster; survival curves were compared using the log-rank test of equality (survival R package). Differences in clinical traits across clusters (for example, antimicrobial exposure score, ANC) were tested using the nonparametric Kruskal–Wallis (kruskaltests R package) and Dunn’s tests (dunn.test R package) or chi-squared test as appropriate. All analyses involving ten or more comparisons were subjected to FDR adjustment to address multiple hypothesis testing.

#### Microbiome comparisons

Differences in microbial taxa, KEGG pathways, richness and diversity across the four BAL clusters were tested using the nonparametric Kruskal–Wallis (kruskaltests R package) and Dunn’s tests (dunn.test R package) with Benjamini–Hochberg correction for multiple hypothesis testing. Differences in microbial taxa and KEGG pathways were also tested using negative binomial generalized linear models, which account for both microbiome composition and size by the inclusion of taxa-specific dispersion factors (edgeR R package)^73^. Associations between microbial taxa and clinical variables (for example, antimicrobial exposure score, in-hospital mortality) were tested using edgeR. AMR transcripts were analyzed by summing across all classes, normalized from counts to input mass using the sample-specific ERCC value, and then further normalized to sample-specific total bacterial mass. Data were visualized with heatmaps showing the cluster means for each variable (pheatmap R package) with individual comparisons shown using box plots (ggplot R package). Causal mediation was used to test whether the association between antimicrobial exposure and mortality was mediated by an antibiotic-induced reduction in certain BAL microorganisms (mediation R package)^74^. Using the latent structural equation framework, we fitted (1) Poisson models for the association between preceding AES and BAL quantity of a certain microorganism, and (2) logistic regression models for the association between BAL quantity of a given microorganism and outcome, independent of AES. Mediation was tested using 500 simulations with bootstrapped confidence intervals; direct and indirect effects were plotted.

#### Pathogen identification

Taxa considered as potential respiratory pathogens were adapted from the CZID Pathogen List (https://czid.org/pathogen_list) with modifications for immunocompromised patients and pathogens specific to the respiratory system. The final list of taxa considered is detailed in Supplementary Table 15. We did not include avirulent viruses, such as torquetenovirus, or bacterial commensals that are infrequently a cause of pulmonary disease, such as *Prevotella* species, coagulase-negative staphylococci, non-diphtheria *Corynebacterium* and viridans group streptococci, although these have at times been implicated in pulmonary disease in immunocompromised individuals. To identify potentially pathogenic viruses, we applied a threshold of viral detection at any level above background (after applying the quality and contamination filters described above). This presence or absence approach was selected to mirror the approach used in clinical respiratory viral panels, which typically dichotomizes any level of detection as present or absent. To identify potentially pathogenic bacteria, we applied a threshold of detection with mass of 10 pg or greater, bacterial dominance of 20% or greater and *z*-score of +2 or greater, where the *z*-score was calculated as the number of standard deviations above the mean of the log_10_-transformed mass values for each microorganism in the cohort. Requiring a minimum mass, dominance and *z*-score was based on the historical framework that bacterial infections occur when microorganisms are present at high mass that is greater than other microorganisms and greater than in other (noninfected) patients, although this may not be true in all instances. Cutoff values were selected empirically after analysis of data distributions and could be exchanged for other cutoffs to alter the balance between sensitivity and specificity of calls. Finally, to identify potentially pathogenic fungi, we applied a threshold of detection with mass of 10 pg or greater and *z*-score of +2 or greater. We did not apply a microbiome dominance cutoff for fungal pathogens because the relationship between organisms in the pulmonary mycobiome is less well understood.

#### Gene expression

Only genes present in more than 25% of samples were used for differential gene expression. To identify individual DEGs, we used a four-way analysis of variance-like approach with negative binomial generalized linear models (edgeR R package). Selected DEGs identified at a threshold FDR ≤ 0.05 were visualized with box plots of variance stabilization-transformed counts. To compute gene set enrichment scores, we used nonparametric gene set variation analysis with Poisson distributions (gsva R package) and the REACTOME set of *n* = 1,554 gene sets^75,76^. Differences in enrichment scores across the BAL clusters were compared using Kruskal–Wallis (kruskaltests R package) and Dunn’s (dunn.test R package) tests; gene sets with significant differences were visualized using dot plots of the mean expression scores (pheatmap R package). Next, cell types contributing to bulk sequencing expression were imputed using CIBERSORTx (Docker version), which uses a user-defined reference single-cell atlas to identify cell-type-specific transcript ratios and impute cell fractions (we selected the lung cell atlas from ref. ^77^)^78,77^. Cell-type-specific gene expression was imputed using CIBERSORTx in high-resolution mode, which uses previously created cell fractions to impute cell-type-specific expression. Finally, lymphocyte receptor repertoires were imputed using ImReP (Linux install), which identifies CDR3 alignments from within bulk gene expression data^79^.

#### Classification and validation

As cluster assignments cannot be directly applied to an external dataset, a classification tool is required to predict cluster assignments. We trained a random forest of 10,000 trees using microbiome taxonomy and lung gene expression datasets as the input, and 1.5× weighting of clusters 3 and 4 given the BAL cluster imbalance (randomForestSRC R package)^80^. Ideal forest parameters determined using tune were similar to the default settings; thus, default settings were used for all other parameters (for example, mtry, nodesize). Forest accuracy was determined using out-of-bag AUCs and a confusion matrix. Variable importance was determined using permutation VIMP (Breiman–Cutler importance) by permuting out-of-bag cases (vimp R package). To validate the classifier, the random forest classifier was applied to microbiome taxonomy and lung gene expression data from the 57 Utrecht BALs and 1 year after BAL non-relapse mortality rates were compared according to predicted BAL cluster type using Kaplan–Meier survival curves with the log-rank test.

### Reporting summary

Further information on research design is available in the Nature Portfolio Reporting Summary linked to this article.

## Online content

Any methods, additional references, Nature Portfolio reporting summaries, source data, extended data, supplementary information, acknowledgements, peer review information; details of author contributions and competing interests; and statements of data and code availability are available at 10.1038/s41591-024-02999-4.

### Supplementary information

Supplementary Information Supplementary Tables 1–15, Figs. 1 and 2, and Data 6–8.

Reporting Summary

Supplementary Data Supplementary Data 1–5 and 9–15.

## Data Availability

Raw sequencing files and instructions on how to download data are available under controlled access on the National Institutes of Health database of Genotypes and Phenotypes at https://ncbi.nlm.nih.gov/projects/gap/cgi-bin/study.cgi?study_id=phs001684.v3.p1. Individual-level data are available indefinitely.

## Code availability

The code used in this study is available on GitHub (https://github.com/zinterm/pedBMT_BALseq).
