# Supplementary material for: Pathobiological signatures of dysbiotic lung injury in pediatric patients undergoing stem cell transplantation
Source: Nat Med. Author manuscript; Available in PMC 2024 Jul 28. (PMC11271406; doi:10.1038/s41591-024-02999-4)
Supplement: Supplementary Information [file NIHMS2010237-supplement-Supplementary_Information.pdf]

nature medicine

Supplementary information https://doi.org/10.1038/s41591-024-02999-4

Pathobiological signatures of dysbiotic lung injury in pediatric patients undergoing stem cell transplantation

In the format provided by the authors and unedited

TITLE: Pathobiologic signatures of dysbiotic lung injury in pediatric patients undergoing stem cell transplantation.

Contents:

PTCTC Site Principal Investigators & Support Staff

Supplementary Table 1: Clinical Background, Stratified by Cluster

Supplementary Table 2: Illness Severity and Clinical Outcomes, Stratified by Cluster

Supplementary Table 3: In-Hospital Mortality by Cluster, Subset for Patients ≤100 Days Post-HCT

Supplementary Table 4: Multivariable Cox Model for In-Hospital Mortality According to BAL Cluster

Supplementary Table 5: Diagnostic Agreement Table for Potential Pathogens Detected in BAL by Clinical and Metagenomic Approaches

Supplementary Table 6: Torquetenovirus Detection, Stratified by Cluster

Supplementary Table 7: Hospital test vs Metagenomic Sequencing

Supplementary Table 8: Characteristics of Non-Survivors

Supplementary Table 9: In-Hospital Mortality Stratified by Hospital-Based vs Metagenomics Results

Supplementary Table 10: Cluster transitions from 1st to 2nd+ BAL

Supplementary Table 11: Cluster prevalence according to 1st vs 2nd+ BAL

Supplementary Table 12: Random Forest Classifier

Supplementary Table 13: Validation Cohort Characteristics

Supplementary Table 14: Validation Cohort Cluster Assignments

Supplementary Figure 1: Multi-Omics Factor Analysis Results

Supplementary Figure 2: Supplementary Figure 2: Microbial Mass Calculation and Batch Contamination

Supplementary Data 6: Univariate Causal Mediation Analysis for % of Association between Antibacterial Exposure & In-Hospital Mortality that is Mediated by Antibacterial Exposure-Induced Changes in BAL Microbes

Supplementary Data 7: Multivariable Causal Mediation Analysis for % of Association between Antibacterial Exposure & In-Hospital Mortality that is Mediated by Antibacterial Exposure-Induced Changes in BAL Microbes

Supplementary Data 8: Univariate Causal Mediation Analysis for % of Association between Anti-Anaerobic Exposure & In-Hospital Mortality that is Mediated by Anti-Anaerobic Exposure-Induced Changes in BAL Microbes

PTCTC Site Principal Investigators & Support Staff

Site	Site Name	Site PI	Specialty	Research Coordinators, Support Staff
AUS	Children's Hospital at Westmead	Peter J. Shaw	BMT	Jun Cai
AZ	Phoenix Children's Hospital	Erin M. Kreml	ICU	Brian Burrows, Jessica Twyford
CA	Children’s Hospital Los Angeles	Hisham Abdel-Azim	BMT	Anju Nair, Sandy Gutierrez
CA	UCLA	Theodore B. Moore	BMT	LaMarr Taylor Smith, Andres Vargas
CA	UCSF-San Francisco	Matt Zinter	ICU	Kevin Magruder, Anne McKenzie
CA	UCSF-Oakland	Nahal Lalefar	BMT	Ad hoc
CAN	Cancer Care Manitoba	Geoffrey D.E. Cuvelier	BMT	Laura Keuler, Erin Richards
CO	Children's Hospital Colorado	Amy K. Keating	BMT	Garrett Donegan, Joanna Wozniak, Steven Kyker
DC	Children's National	Benjamin Hanisch	ID	Alexa Yarish
FL	Nicklaus Children's	Kamar Godder	BMT	Guido Elias, Kristofer Rosales
FL	University of Florida, Gainesville	Paul Castillo	BMT	Giselle Moore-Higgs, Beate Greer, Joshua Terrell
GA	Emory	Muna Qayed	BMT	Andrea Peters, Ashley Griffin
IL	Lurie Children's	Sonali Chaudhury	BMT	Olga Jonas, Dian’Ella Ramsey
IN	Riley Children's	Courtney M. Rowan	ICU	Kirsten Ramberg, Jill Mazurczyk, Andrea Hudgins
MA	Boston Children's	Christine N. Duncan	BMT	Lauren Leonard, Sophie Silverstein, Miranda Fry
MI	Helen DeVos Children's	Aly Abdel-Mageed	BMT	Jennifer Hanenburg
MN	University of Minnesota	Janet R. Hume	ICU	Lexie Goertzen
MN	Mayo Clinic	Shakila P. Khan	BMT	Julia Byrne, Becky Winslow-Rain
MS	Children's Hospital Mississippi	Dereck B. Davis	BMT	Sarah Elkin, Katie Williams, Michelle Kimble
NC	Duke University	Paul L. Martin	BMT	Lucy Harris, Linda Brown
NJ	Hackensack	Shira J. Gertz	ICU	Elana Smilow, Gina Dovi, Jeanette Haugh
NY	Columbia	Prakash Satwani	BMT	Aaleen Cox, Chez Brivett, Brian Volonte
NY	Memorial Sloan Kettering/Cornell	James S. Killinger	ICU	Jennifer Sollitto, Jennifer Direnzo
OH	Cleveland Clinic	Rabi Hanna	BMT	Rawan Nawabit, Alexis Smith
OH	Nationwide	Jeffrey J. Auletta	BMT	Clelie Peck, Kelly Ortman, Mary Scholz
PA	Children’s Hospital of Philadelphia	Julie C. Fitzgerald	ICU	Jenny Bush, Mary Diliberto, Martha Sisko
PA	Pittsburg	Jessie L. Barnum	BMT	Olga Greg
SC	Medical University South Carolina	Michelle P. Hudspeth	BMT	Jared Hortman
TN	St. Jude	Caitlin Hurley	ICU	Ad hoc
TX	MD Anderson	Kris M. Mahadeo	BMT	LaTarsha Williams, So Jung Hong
TX	Methodist San Antonio	Troy C. Quigg	BMT	Candace Taylor, Marisa Palacios
WI	Medical College of Wisconsin	Rachel Phelan	BMT	Adam Fiebelkorn, Melissa Schussman

Supplementary Table 1: Clinical Background, Stratified by Cluster

**Demographics (n=229 patients)**	**Cluster 1 (n=101)**	**Cluster 2 (n=59)**	**Cluster 3 (n=43)**	**Cluster 4 (n=26)**	**Sig.**
Age (median years, IQR)	11.5 (4.5-17.4)	11.0 (5.4-15.1)	10.5 (5.1-14.5)	10.6 (4.2-16.6)	P=0.924
Sex (male)	64 (64%)	40 (68%)	18 (42%)	11 (42%)	**P=0.013**
Race					P=0.071
- White, not Latinx	68 (67%)	31 (53%)	27 (63%)	14 (54%)	
- Black	9 (9%)	9 (15%)	8 (19%)	3 (12%)	
- Other/multiple	11 (11%)	4 (7%)	4 (9%)	7 (27%)	
- Asian/PI	10 (10%)	11 (19%)	3 (7%)	1 (4%)	
- Native American	1 (1%)	0 (0%)	0 (0%)	1 (4%)	
- Unknown	2 (2%)	4 (7%)	1 (2%)	0 (0%)	
Ethnicity - Latino/Hispanic	25 (25%)	11 (19%)	12 (28%)	11 (42%)	P=0.142
Region					**P=1.652x10^{-4}**
- Australia	9 (7)	12 (16)	2 (4)	1 (3)	
- Canada	1 (1)	0 (0)	4 (9)	0 (0)	
- USA – Atlantic	11 (9)	8 (11)	9 (20)	0 (0)	
- USA – Midwest	21 (17)	8 (11)	5 (11)	5 (16)	
- USA – Northeast	9 (7)	6 (8)	4 (9)	1 (3)	
- USA – Pacific	61 (48)	37 (50)	13 (29)	24 (75)	
- USA – South	7 (6)	1 (1)	6 (13)	0 (0)	
- USA – Southwest	8 (6)	2 (3)	2 (4)	1 (3)	
**Medical History (n=229 patients)**					
Disease					
- Leukemia[a]	55 (54%)	35 (59%)	21 (49%)	14 (54%)	P=0.599
- Inborn errors of immunity[b]	19 (19%)	9 (15%)	8 (19%)	4 (15%)	
- Non-malignant hematologic[c]	7 (7%)	7 (12%)	9 (21%)	4 (15%)	
- Solid tumor[d]	7 (7%)	2 (3%)	3 (7%)	2 (8%)	
- Lymphoma[e]	7 (7%)	3 (5%)	2 (5%)	0 (0%)	
- Inborn errors of metabolism[f]	6 (6%)	3 (5%)	0 (0%)	2 (8%)	
HCT Type					
- Allogeneic	92 (91%)	57 (97%)	40 (93%)	24 (92%)	P=0.520
- Bone marrow	- 38 (41%)	- 27 (47%)	- 20 (50%)	- 7 (29%)	
- Peripheral blood	- 36 (39%)	- 24 (42%)	- 15 (38%)	- 13 (54%)	
- Umbilical cord blood (UCB)	- 18 (20%)	- 6 (11%)	- 5 (13%)	- 4 (17%)	
- Autologous	9 (9%)	2 (3%)	3 (7%)	2 (8%)	
HLA match (allogeneic only)					P=0.950
- Matched related donor	20 (22%)	13 (23%)	7 (18%)	4 (21%)	
- Matched unrelated donor (inc. 6/6 UCB)	18 (20%)	14 (25%)	9 (23%)	8 (33%)	
- Mismatched related donor (haplo)	27 (29%)	13 (23%)	11 (28%)	6 (25%)	
- Mismatched unrelated donor (inc. <6/6 UCB)	27 (29%)	17 (30%)	13 (33%)	5 (21%)	
Conditioning Agents Used[g]					
- Backbone agent					
- Busulfan	33 (33%)	24 (41%)	16 (37%)	13 (50%)	P=0.393
- Melphalan	28 (28%)	16 (27%)	11 (26%)	8 (31%)	P=0.973
- Total body irradiation (TBI)	31 (31%)	14 (24%)	9 (21%)	5 (19%)	P=0.472
- Other alkylating agent					
- Cyclophosphamide	43 (43%)	21 (36%)	16 (37%)	11 (42%)	P=0.814
- Thiotepa	29 (29%)	17 (29%)	14 (33%)	6 (23%)	P=0.870
- Antimetabolite					
- Clofarabine	6 (6%)	4 (7%)	2 (5%)	3 (12%)	P=0.711
- Fludarabine	61 (60%)	41 (69%)	29 (67%)	15 (58%)	P=0.572
- Serotherapy (ATG or Alemtuzumab)	51 (51%)	33 (56%)	21 (49%)	14 (54%)	P=0.881

Legend: Characteristics compared using two-sided Kruskal-Wallis test or Chi-squared test as appropriate.

Supplementary Table 2: Clinical Presentation and Outcomes, Stratified by Cluster

Characteristics at Enrollment (n=278 BALs)	Cluster 1 N=127	Cluster 2 N=74	Cluster 3 N=45	Cluster 4 N=32	Sig
Days from HCT to BAL (n, %)	105 (28-309)	218 (50-476)	82 (37-146)	121 (47-351)	**P=0.016**
Days from Symptoms to BAL[a] (n, %)	6 (2-14)	11 (3-30)	8 (2-22)	9 (3-28)	P=0.190
Clinical Presentation Symptoms (n, %)					
- Lower respiratory symptoms)	104 (82%)	71 (96%)	43 (96%)	31 (97%)	**P=0.002**
- Hypoxia ≤96%	93 (73%)	46 (62%)	38 (84%)	25 (78%)	P=0.051
- Abnormal chest x-ray	78/88 (89%)	43/56 (77%)	32/39 (82%)	21/24 (88%)	P=0.271
- Abnormal chest CT	102/107 (95%)	51/51 (100%)	31/34 (91%)	25/26 (96%)	P=0.243
- Worse PFTs	10 (8%)	2 (3%)	1 (2%)	3 (9%)	P=0.248
Oxygen Required Prior to BAL					
- No	79 (62%)	47 (64%)	16 (36%)	14 (44%)	**P=0.004**
- Yes	48 (38%)	27 (36%)	29 (64%)	18 (56%)	
Oxygen Type Prior to BAL					
- None	79 (62%)	47 (64%)	16 (36%)	14 (44%)	**P=0.019**
- Nasal cannula or face mask	18 (14%)	10 (14%)	9 (20%)	4 (13%)	
- HFNC, NIV, or Intubation	30 (24%)	17 (23%)	20 (44%)	14 (44%)	
Comorbidities at time of BAL (n, %)					
- Engraftment syndrome	10 (8%)	3 (4%)	2 (4%)	0 (0%)	P=0.296
- GVHD active at time of BAL	29 (25%)	28 (39%)	11 (27%)	15 (48%)	**P=0.040**
- GVHD ever preceding BAL	46 (40%)	42 (58%)	18 (44%)	20 (65%)	**P=0.019**
- Heart failure or reduced function	4 (3%)	2 (3%)	3 (7%)	2 (6%)	P=0.607
- Kidney injury	16 (13%)	7 (9%)	14 (31%)	10 (31%)	**P=0.001**
- Pericardial effusion	13 (10%)	3 (4%)	5 (11%)	4 (12%)	P=0.366
- Pulmonary hemorrhage/hemoptysis	14 (11%)	2 (3%)	3 (7%)	4 (13%)	P=0.158
- Sepsis	16 (13%)	11 (15%)	7 (16%)	3 (9%)	P=0.841
- TA-TMA	10 (8%)	5 (5%)	3 (7%)	5 (16%)	P=0.341
- VOD/SOS	11 (9%)	3 (4%)	6 (13%)	4 (13%)	P=0.280
Immunologic Function Prior to BAL					
- WBC (median. IQR)	4,230 (2,300-7,300)	5,700 (2,500-12.5k)	4,400 (1,500-7,790)	4,685 (3,400-8,750)	P=0.098
- ANC (median, IQR)	2,774 (1,233-4,581)	4,059 (1,960-7,155)	2,490 (940-5,125)	3,344 (2,007-5,640)	**P=0.029**
- ANC <0.5 $x10^9$/L (n, %)	19 (15%)	5 (7%)	9 (20%)	1 (3%)	**P=0.047**
- ALC (median, IQR)	423 (179-994)	414 (131-1,102)	417 (155-1,106)	405 (196-1,187)	P=0.997
- ALC <0.2 $x10^9$/L (n, %)	34 (27%)	21 (28%)	13 (29%)	8 (25%)	P=0.977
**Outcomes**					
**Intensive Care After BAL** (yes/no)	64 (50%)	29 (39%)	34 (76%)	20 (62%)	**P=0.001**
**Mechanical Ventilation After BAL**					
- ≥ 24 hours	49 (39%)	23 (31%)	26 (58%)	16 (50%)	**P=0.022**
- ≥ 7 days	33 (26%)	16 (22%)	23 (51%)	14 (44%)	**P=0.001**
**In-Hospital Mortality (n=229 patients)**	N=101	N=59	N=43	N=26	
- By 14 days	6 (6%)	2 (3%)	3 (7%)	6 (23%)	**P=0.012**
- By 28 days	9 (9%)	4 (7%)	7 (16%)	7 (27%)	**P=0.032**
- All In-hospital	14 (14%)	8 (14%)	14 (33%)	9 (35%)	**P=0.008**
**In-Hospital Mortality if requiring oxygen prior to BAL (n=103 patients)**	N=37	N=23	N=28	N=15	
- By 14 days	2 (5%)	2 (9%)	3 (11%)	6 (40%)	**P=0.006**
- By 28 days	4 (11%)	4 (17%)	7 (25%)	7 (47%)	**P=0.035**
- All In-hospital	8 (22%)	7 (30%)	14 (50%)	9 (60%)	**P=0.022**

Legend: Since some patients had multiple BALs, in-hospital mortality was calculating using the most recent BAL. Characteristics compared using two-sided Kruskal-Wallis test or Chi-squared test, as appropriate.

Supplementary Table 3: In-Hospital Mortality by Cluster, Subset for Patients ≤100 Days Post-HCT

	Cluster 1 N=51	Cluster 2 N=24	Cluster 3 N=25	Cluster 4 N=12	Significance
**In-Hospital Mortality**					
- **Yes**	6 (12%)	7 (29%)	10 (40%)	6 (50%)	P=0.019
- **No**	45 (88%)	17 (71%)	15 (60%)	6 (50%)	

Legend: Only the most recent BAL for each patient was used. Significance tested with two-sided Chi-squared test.

Supplementary Table 4: Multivariable Cox Model for In-Hospital Mortality

	Hazard Ratio (95% CI)	Significance
**BAL Cluster (relative to Cluster 1)**		
- **Cluster 2**	1.19 (0.44-3.20)	P=0.734
- **Cluster 3**	2.63 (1.10-6.31)	**P=0.030**
- **Cluster 4**	3.43 (1.34-8.80)	**P=0.010**
**Age (years)**	0.99 (0.94-1.05)	P=0.826
**Biologic Sex (male reference)**	0.64 (0.33-1.25)	P=0.191
**ANC**	1.04 (0.98-1.10)	P=0.268
**ALC**	0.35 (0.17-0.75)	**P=0.007**
**GVHD (yes/no)**	1.17 (0.59-2.33)	P=0.658

Legend: Cox multivariable model for in-hospital mortality. Significance was assessed with two-sided alpha threshold <0.05.

Supplementary Table 5: Diagnostic Agreement Table for Potential Pathogens Detected in BAL by Clinical and Metagenomic Approaches

	C	C+M	M		C	C+M	M		C	C+M	M
**Community Viruses**				**Cultivable Bacteria**				**Fungi**			
Adenovirus	4	4	7	Achromobacter	0	1	0	Alternaria	0	0	4
Coronavirus	1	3	5	Bacillus	0	0	1	Aspergillus [a]	13	2	16
Influenza virus	1	1	3	Citrobacter	0	0	1	Candida	3	2	19
Metapneumovirus	0	1	1	Escherchia	1	3	8	Cladosporium	0	0	3
Parainfluenza virus	1	3	3	Enterococcus	2	1	2	Cryptococcus	0	0	1
Rhinovirus	5	27	20	Haemophilus	3	8	3	Exophiala	0	0	4
RSV	0	2	2	Klebsiella	2	1	4	Exserohilum	0	0	8
				Moraxella	1	3	2	Fusarium	1	0	17
**Herpesviruses**				Pseudomonas	1	9	2	Mucor	0	1	6
Cytomegalovirus	9	14	9	Salmonella	0	0	1	Pneumocystis	1	2	6
Epstein-Bar Virus	3	0	8	Staphylococcus	3	3	1	Saccharomyces	0	1	13
Herpes Simplex Virus 1	2	0	6	Stenotrophomonas	1	4	12				
Human Herpes Virus-6	5	4	13	Streptococcus	1	1	1	**Parasites**			
Human Herpes Virus-7	1	0	3					Acanthamoeba	0	0	4
Varicella Zoster Virus	0	0	0	**Atypical Bacteria**				Toxoplasma	0	0	4
				Chlamydia	0	0	1				
**Other Viruses**				Legionella	0	0	0				
Bocavirus	0	0	4	Mycoplasma	0	0	4				
BK polyoma	0	0	1	Ureaplasma	0	0	1				
KI polyoma	0	0	10								
LCMV	0	0	1	**Fastidious Bacteria**							
Parvovirus B19	0	0	1	Actinomyces	0	1	1	**Total:**	69	104	256
Rubella	0	0	1	Mycobacteria	2	0	0				
WU polyoma	0	0	8	Nocardia	2	1	0				

Legend: Potentially pathogenic microbes detected by clinical testing (C), metagenomic testing (M), or both clinical and metagenomic testing (C+M). Samples with multiple pathogens will contribute multiple entries in the table. Refer to **Data File 4** for list of potentially pathogenic taxa. Microbes not typically considered pulmonary pathogens, such as *S.epidermidis*, *P.melaninogenica*, and *R.mucilaginosa*, were not included. Refer to **Data File 5** for patient-level analysis. Refer to text for algorithm for discriminating potential pathogens from the background pulmonary microbiome. [a] While overlap between clinical and sequencing-based Aspergillus detection was low, of the n=13 Aspergillus detected clinically without NGS confirmation, all showed "one" or "rare" colonies on culture and only one was associated with a positive BAL galactomannan (0.589). Additionally, n=10 samples showed a positive BAL galactomannan and yet had no Aspergillus detected clinically or by sequencing with the above thresholds, suggesting a high rate of false-positivity for hospital-based testing.[28,29]

Supplementary Table 6: Torquetenovirus Detection, Stratified by Cluster

All BALs (n=278)	Cluster 1 N=127	Cluster 2 N=74	Cluster 3 N=45	Cluster 4 N=32	Significance
**TTV Detected**					
- **Yes**	10 (8%)	23 (31%)	12 (27%)	10 (31%)	$P=9.1x10^{-5}$
- **No**	117 (92%)	51 (69%)	33 (73%)	22 (69%)	

Legend: BAL detection of Alphatorquevirus, Betatorquevirus, or Gammatorquevirus RNA above background controls. Significance tested with two-sided Chi-squared test.

Supplementary Table 7: Comparison of Hospital-Based vs Metagenomics Results

	Metagenomics Positive	Metagenomics Negative	McNemar's Test
**Any pathogen**			
- Hospital testing positive	102	14	$P=5.7x10^{-14}$
- Hospital testing negative	91	71	
**Any virus**			
- Hospital testing positive	59	17	$P=3.7x10^{-5}$
- Hospital testing negative	51	151	
**Any bacteria**			
- Hospital testing positive	35	16	$P=6.4x10^{-4}$
- Hospital testing negative	42	185	
**Any eukaryote**			
- Hospital testing positive	10	15	$P=6.3x10^{-10}$
- Hospital testing negative	73	180	

Legend: Comparison of 2 diagnostic tests performed on same samples using two-sided McNemar's test.

Supplementary Table 8: Characteristics of Non-Survivors

BAL	Age	Sex	Disease	HSCT Type	HSCT Source	HSCT Donor	ANC	ALC	HCT to BAL (days)	Pulmonary Diagnosis	BAL to death (Days)	Cause of Death	Contributors
BAL_001	2.0	F	PID	Allo	PB	MUD	4.30	0.83	97	LRTI	24	Pichia kluyveri, HHV6, Polyomavirus3	GVHD
BAL_012	11.5	F	HLH	Allo	BM	MUD	1.74	0.41	31	LRTI	47	RhinovirusB, HSV1, CMV, Polyomavirus3	Renal failure
BAL_016	10.0	M	SAA	Allo	BM	MRD	7.14	0.27	87	LRTI	80	Stenotrophomonas maltophilia	Renal failure, TMA
BAL_018	19.8	F	Leuk.	Allo	UCB	MMUD	1.92	0.48	37	LRTI	71	RhinovirusC	Pulmonary hemorrhage
BAL_019	12.5	M	Leuk.	Allo	PB	MUD	2.01	0.19	69	LRTI	8	CMV, HHV6	Renal failure, VOD
BAL_020	4.9	F	SAA	Allo	BM	MRD	5.21	0.15	100	IPS	15	IPS	GVHD, renal failure
BAL_021	1.4	M	PID	Allo	PB	MMRD	0.00	0.00	40	LRTI	13	Stenotrophomonas maltophilia, Candida tropicalis	Renal failure
BAL_022	20.1	F	Heme	Allo	PB	MMRD	4.88	0.42	70	LRTI	16	Stenotrophomonas maltophilia	
BAL_023	2.5	F	SAA	Allo	UCB	MMUD	3.63	1.30	74	LRTI	45	CMV, HHV6, Bocavirus	
BAL_033	20.4	M	SAA	Allo	BM	MMUD	11.14	0.23	61	LRTI	34	Toxoplasma	Renal failure
BAL_039	15.6	M	Leuk.	Allo	BM	MUD	0.15	0.65	132	LRTI	33	Enterococcus faecium, CMV	GVHD
BAL_044	8.5	F	ST	Auto			2.26	0.70	18	LRTI	18	Stenotrophomonas maltophilia, CMV, HHV6	
BAL_054	6.6	M	ST	Auto					255	IPS	5	IPS	Sepsis (culture negative)
BAL_057	19.0	M	Leuk.	Allo	PB	MUD			9	LRTI	1	Stenotrophomonas maltophilia	Sepsis
BAL_063	0.5	F	PID	Allo	UCB	MUD	0.18	2.09	22	LRTI	63	Stenotrophomonas maltophilia, PCP	Sepsis
BAL_068	17.1	F	Metab.	Allo	BM	MUD	3.68	0.20	47	Sepsis	33	Non-pulmonary sepsis (Strep viridans)	GVHD, pulmonary hemorrhage
BAL_069	14.6	F	Leuk.	Allo	BM	MRD	0.24	0.07	104	LRTI	11	Toxoplasma, RhinovirusC, AdenovirusB	GVHD, renal failure, pericardial effusion, sepsis
BAL_076	16.7	F	Leuk.	Allo	PB	MMRD	1.66	0.32	191	LRTI	7	HCOV 229E, CMV, HHV7, BKV, Polyomavirus3	GVHD, renal failure, pericardial effusion
BAL_077	4.2	F	PID	Allo	PB	MMRD	5.61	0.00	78	LRTI	4	HCOV OC43	
BAL_082	10.8	F	Leuk.	Allo	PB	MMUD	0.91	0.03	37	LRTI	9	AdenovirusC, RhinovirusA, CMV	Renal failure, VOD
BAL_086	22.1	M	Leuk.	Allo	PB	MRD	3.59	0.14	19	LRTI	3	Achromobacter xylosoxidans, Rhizopus, RhinovirusC	GVHD, renal failure, sepsis, VOD
BAL_099	3.6	M	ST	Auto			3.06	0.33	58	LRTI	11	HHV7	Pulmonary hemorrhage, TMA, VOD
BAL_108	7.5	M	Lymph.	Allo	PB	MMRD	4.16	0.22	716	LRTI	187	E.coli, Polyomavirus3, CMV	GVHD, renal failure, pulmonary hemorrhage
BAL_109	19.3	F	Leuk.	Allo	PB	MMRD	2.12	0.37	113	LRTI	2	Polyomavirus4, HSV1	Heart failure
BAL_113	9.0	F	PID	Allo	BM	MUD	2.12	0.17	263	LRTI	142	Pseudomonas aeruginosa, AdenovirusA, AdenovirusC, HHV6	GVHD, renal failure, pulmonary hemorrhage
BAL_118	12.3	F	Leuk.	Allo	PB	MMRD	12.53	0.35	28	IPS	49	IPS	GVHD, pulmonary hemorrhage
BAL_122	13.0	M	Leuk.	Allo	BM	MRD			172	IPS	4	IPS	GVHD
BAL_127	17.4	M	Leuk.	Allo	UCB	MMRD	0.10	0.08	18	LRTI	105	E.coli	
BAL_128	12.5	F	ST	Auto			12.35	1.25	448	IPS	97	IPS	Pericardial effusion
BAL_129	1.6	F	Leuk.	Allo	UCB	MMUD	0.15	0.26	219	LRTI	283	Rhizopus, RhinovirusA	Pericardial effusion
BAL_131	10.9	M	Leuk.	Allo	BM	MRD	0.04	0.02	630	LRTI	7	Candida parapsilosis, hMPV, Bocavirus	GVHD, renal failure, sepsis, VOD
BAL_140	13.3	M	Leuk.	Allo	PB	MUD	26.11	0.00	12	LRTI	111	RhinovirusA	Renal failure
BAL_142	14.5	M	Leuk.	Allo	BM	MMUD	0.08	0.00	13	LRTI	15	RhinovirusC, HHV7	
BAL_166	12.8	F	Leuk.	Allo	PB	MMRD	12.18	0.52	63	LRTI	42	E.coli, HCOV NL63, CMV	GVHD, renal failure, sepsis
BAL_185	17.2	F	Leuk.	Allo	PB	MRD	4.08	0.69	91	LRTI	63	E.faecium, hMPV, CMV	GVHD
BAL_196	15.3	F	Leuk.	Allo	PB	MUD	2.73	0.12	130	LRTI	26	Aspergillus fumigatus, Bocavirus	GVHD, heart failure, renal failure, sepsis
BAL_204	20.1	M	Lymph.	Allo	PB	MMRD	3.08	0.75	103	LRTI	47	Aspergillus flavus, Candida parapsilosis, RSV, CMV	GVHD
BAL_210	12.5	M	Lymph.	Allo	UCB	MMUD	1.10	1.23	37	LRTI	20	HHV6	VOD
BAL_217	10.5	M	Leuk.	Allo	BM	MUD	9.49	0.00	48	IPS	17	IPS	
BAL_242	4.9	M	Leuk.	Allo	PB	MMRD	23.94	0.25	42	LRTI	4	RhinovirusC	GVHD
BAL_245	17.1	F	Leuk.	Allo	BM	MMUD	5.51	0.75	224	IPS	22	IPS	GVHD
BAL_247	7.1	F	Leuk.	Allo	PB	MMRD	6.11	0.69	30	LRTI	24	Fusarium	GVHD
BAL_270	10.2	F	Leuk.	Allo	PB	MMUD	2.22	0.06	529	LRTI	13	RhinovirusA, Acanthamoeba	GVHD, renal failure
BAL_273	1.8	M	Metab.	Allo	UCB	MMUD	1.96	0.41	35	LRTI	32	E.coli, CMV, Polyomavirus4	
BAL_274	11.5	M	Leuk.	Allo	UCB	MUD			753	LRTI	13	RhinovirusA, CMV, HSV1	Sepsis (culture negative)

Legend: Abbreviations: Heme (non-malignant hematologic disorder), HLH (hemophagocytic lymphohistiocytosis), Leuk (leukemia), Lymph. (lymphoma), PID (primary immunodeficiency), SAA (severe aplastic anemia), ST (solid tumor).

Supplementary Table 9: In-Hospital Mortality Stratified by Hospital-Based vs Metagenomics Results

	Metagenomics Positive	Metagenomics Negative	Significance
**Any pathogen**			
- Hospital testing positive	21/79 (27%)	1/13 (8%)	P=0.190
- Hospital testing negative	15/80 (19%)	8/57 (14%)	

Legend: Significance assessed with the two-sided Chi-squared test.

Supplementary Table 10: Cluster transitions from 1st to 2nd+ BAL

	2nd+ sample			
**1st sample**	**Cluster 1**	**Cluster 2**	**Cluster 3**	**Cluster 4**
**Cluster 1**	9	6	2	9
**Cluster 2**	5	10	0	0
**Cluster 3**	0	0	0	2
**Cluster 4**	3	0	1	2

Legend: Raw data for BAL cluster transitions for each patient contributing ≥2 BALs to the cohort.

Supplementary Table 11: Cluster prevalence according to 1st vs 2nd+ BAL

	1st BAL (n=229)	2nd+ BAL (n=49)
**Cluster 1**	111 (49%)	17 (33%)
**Cluster 2**	57 (25%)	16 (35%)
**Cluster 3**	42 (18%)	3 (6%)
**Cluster 4**	19 (8%)	13 (27%)

Legend: Significance assessed with two-sided Chi-squared test ($p=4.46 \times 10^{-4}$).

Supplementary Table 12: Random Forest Classifier

	Predicted:			
Observed:	**Cluster 1**	**Cluster 2**	**Cluster 3**	**Cluster 4**
**Cluster 1**	115	12	0	0
**Cluster 2**	8	51	10	5
**Cluster 3**	1	7	37	0
**Cluster 4**	5	10	0	17

Legend: Derivation Cohort confusion matrix of actual cluster assignments vs. those predicted by the random forest classifier using out-of-bag (OOB) predictions.

Supplementary Table 13: Validation Cohort Characteristics (Utrecht, The Netherlands)

**Demographics (n=57 patients)**	
Age (median years, IQR)	3.1 (IQR 1.3-13.5)
Sex (male)	33 (57.9%)
Race<br>- Caucasian<br>- African/North African<br>- Middle Eastern<br>- Asian/SE Asian<br>- Eastern European/Russian<br>- Multiracial/Other	 42 (80.1%)<br>5 (9.6%)<br>2 (3.9%)<br>1 (1.9%)<br>1 (1.9%)<br>1 (1.9%)
**Medical History (n=57 patients)**	
Disease<br>- Leukemia[a]<br>- Inborn errors of immunity[b]<br>- Inborn errors of metabolism[c]<br>- Non-malignant hematologic[d]<br>- Lymphoma[e]	 24 (42.1%)<br>14 (24.6%)<br>13 (22.8%)<br>5 (8.8%)<br>1 (1.7%)
HCT Type<br>- Allogeneic<br>- Bone marrow<br>- Peripheral blood<br>- Umbilical cord blood (UCB)	 57 (100.0%)<br>- 13 (22.8%)<br>- 4 (7.0%)<br>- 40 (70.2%)
HLA match<br>- Matched related donor (BM/PB only)<br>- Matched unrelated donor (BM/PB only)<br>- Mismatched unrelated donor (BM/PB only)<br>- Matched UCB<br>- Mismatched UCB	 6 (10.5%)<br>10 (17.5%)<br>1 (1.7%)<br>15 (26.3%)<br>25 (43.9%)
Conditioning Regimen[f]<br>- Alkylating agent<br>- Busulfan<br>- Cyclophosphamide<br>- Melphalan<br>- Etoposide<br>- Treosulfan<br>- Antimetabolite<br>- Clofarabine<br>- Fludarabine<br>- Serotherapy (ATG or Alemtuzumab)<br>- Total body irradiation (TBI)	 <br>49 (86.0%)<br>15 (26.3%)<br>3 (5.3%)<br>7 (12.3%)<br>1 (1.8%)<br><br>9 (15.8%)<br>41 (71.9%)<br>40 (70.2%)<br>6 (10.5%)
**Characteristics at time of BAL (n=57 patients)**	
Days from HCT to BAL (n, %)	70 (IQR 21-104)
Comorbidities prior to BAL (n, %)<br>- acute GVHD preceding BAL<br>- any grade<br>- grade 3 or 4<br>- chronic GVHD preceding BAL<br>- any stage<br>- extensive	 <br>22 (38.6%)<br>7 (12.3%)<br><br>11 (19.3%)<br>4 (7.0%)

- VOD preceding BAL<br>- any severity	 6 (10.5%)
BAL Clinical Microbiology Results (n, %)[g]<br>- Any positive<br>- Bacterial<br>- Viral<br>- Fungal/Protozoal<br>- More than 1 organism	 23 (40.4%)<br>4 (7.0%)<br>18 (31.6%)<br>12 (21.1%)<br>10 (17.5%)
**Outcomes at 365 days post BAL (n=57 patients)**	
Survival to last follow-up<br>Non-relapse mortality<br>Relapse	31 (52.6%)<br>19 (35.1%)<br>7 (12.3%)

Legend: [a] includes ALL (n=13), AML (n=10), JMML (n=1). [b] includes SCID (n=3), HLH (n=2), CGD (n=4), WAS (n=1), other (n=4). [c] includes Hurler syndrome (n=6), metachromatic leukodystrophy (n=3), other (n=4). [d] includes SAA (n=2), Fanconi anemia (n=1), thalassemia (n=1), other (n=1). [e] includes Hepatosplenic T-cell lymphoma (n=1). [f] Patients may have received multiple agents in the same or multiple categories. [g] Bacteria included Moraxella catarrhalis (n=1), Mycobacterium kansasii (n=1), Staphylococcus aureus (n=1), Stenotrophomonas maltophilia (n=1). Viruses included Adenovirus (n=5), CMV (n=1), Coronavirus (n=3), EBV (n=1), HHV-6 (n=3), HSV-2 (n=1), Metapneumovirus (n=1), Parainfluenzavirus 2 or 4 (n=1), RSV (n=1), Rhinovirus (n=11). Fungi/protozoa included Aspergillus (n=7 by positive galactomannan but no culture growth, n=2 by culture growth but negative galactomannan), Candida (n=1), Cladosporium (n=1), Pneumocystis (n=1).

Supplementary Table 14: Validation Cohort Cluster Assignments

BAL Classification	N=57	Average Ratio of Assigned Cluster vs Cluster 1	Average Ratio of Assigned Cluster vs Cluster 2	Average Ratio of Assigned Cluster vs Cluster 3	Average Ratio of Assigned Cluster vs Cluster 4
Cluster 1	21	--	3.4	28.9	5.1
Cluster 2	11	2.6	--	7.2	2.9
Cluster 3	24	4.4	1.3	--	6.6
Cluster 4	1	2.3	1.3	13.8	--

Legend: Random forest classifier for BAL cluster, assigned to validation cohort. Final assignments determined as most likely outcome from applying random forest. Ratio of assignments to designated class vs each other class was computed for each of the n=57 patients in the validation cohort, and the mean ratios across each assigned cluster are listed.

Supplementary Figure 1: Multi-Omics Factor Analysis Results

Legend: (A) Percent of Variance Explained by MOFA Factors The overall variance explained by the sum of all fifteen latent factors. Greater total variance was explained for lung gene expression variables than for microbiome variables. **(B) MOFA Factor Correlation**. Fifteen latent factors were calculated for each patient, using all available microbiome and gene expression data. Latent factors showed minimal cross-correlation. **(C) MOFA Factor Importance.** Scaled weights for the top 5 variables (out of all input variables) that explained the most variance for each factor. **(D) Ideal number of clusters.** Elbow plot, silhouette plot, and gap statistic plot depicting ideal number of clusters based on umap of MOFA factors. Based on these metrics, 4 clusters were chosen. **(E) Factor correlation with clinical values.** Spearman correlation coefficients were calculated and are displayed if $p<0.05$.

Supplementary Figure 2: Microbial Mass Calculation and Batch Contamination

Legend: (A) 25pg of External RNA Consortium Controls (ERCC) was spiked into each sample prior to library preparation. Each aliquot of ERCC contains 92 different transcripts in different quantities. The scatterplots depict the input mass of each ERCC on the x-axis and the output transcript count of each ERCC on the y-mass. These data demonstrate log-linear amplification across 7 orders of magnitude, allowing for back-calculation of original RNA mass of each BAL sample and its microbiome constituents. **(B)** Water and HeLa "no-template" controls underwent RNA extraction, library preparation, and sequencing with each batch of samples. The mass of microbial contaminants in the negative controls of each batch was back-calculated using the ERCC equation. This box-whisker plot depicts the median and IQR of contaminants detected in each batch. To address ubiquitous contamination, the mass of each microbe in each BAL sample was reduced by the (mean + 2 SD) of what was observed in the control samples of the respective batch**.**

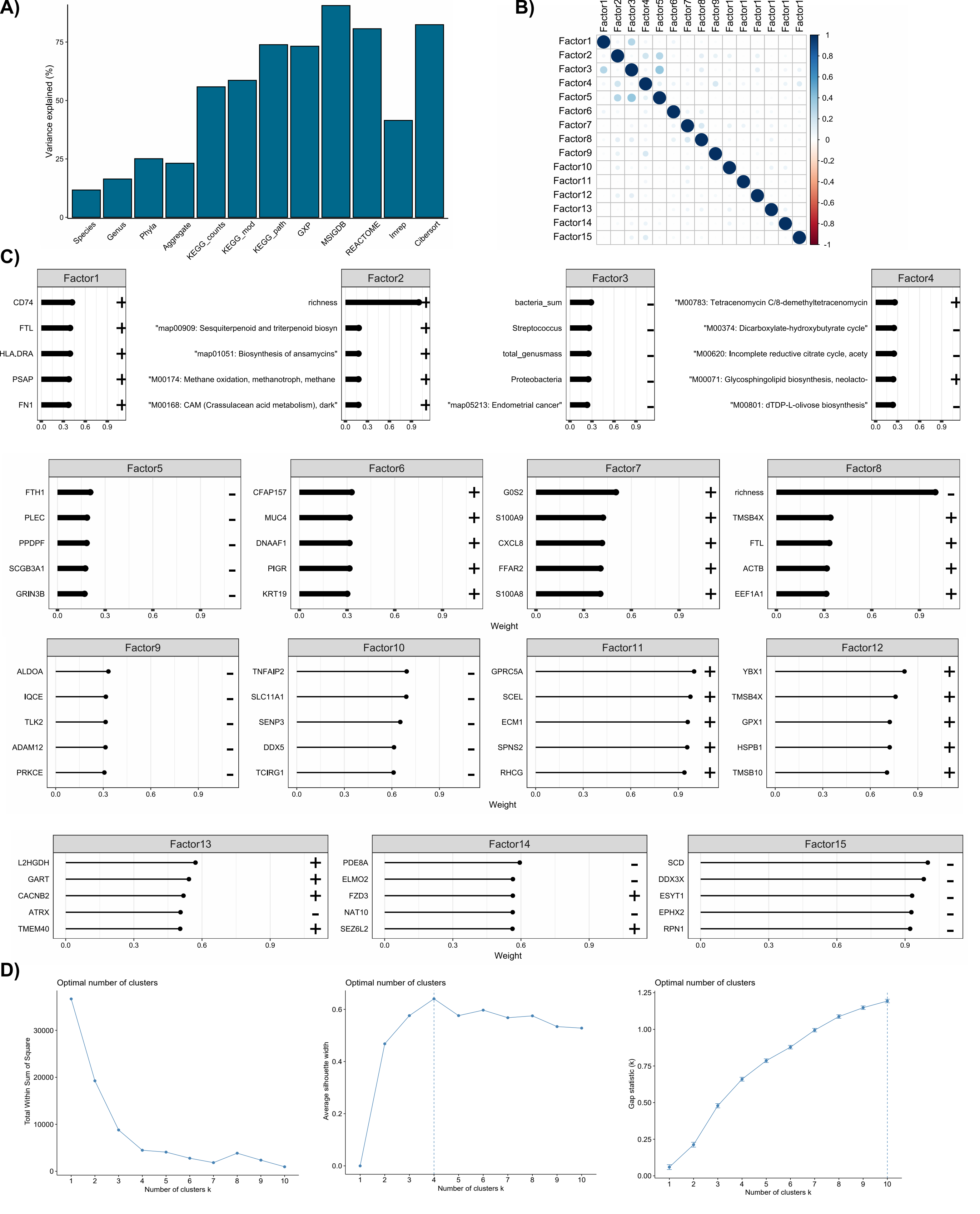

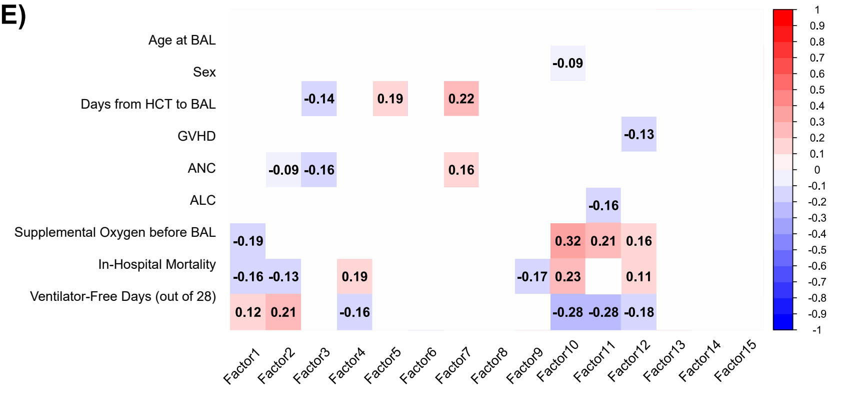

Legend: (A) Percent of Variance Explained by MOFA Factors The overall variance explained by the sum of all fifteen latent factors. Greater total variance was explained for lung gene expression variables than for microbiome variables. **(B) MOFA Factor Correlation.** Fifteen latent factors were calculated for each patient, using all available microbiome and gene expression data. Latent factors showed minimal cross-correlation. **(C) MOFA Factor Importance.** Scaled weights for the top 5 variables (out of all input variables) that explained the most variance for each factor. **(D) Ideal number of clusters.** Elbow plot, silhouette plot, and gap statistic plot depicting ideal number of clusters based on umap of MOFA factors. Based on these metrics, 4 clusters were chosen. **(E) Factor correlation with clinical values.** Spearman correlation coefficients were calculated and are displayed if p<0.05.

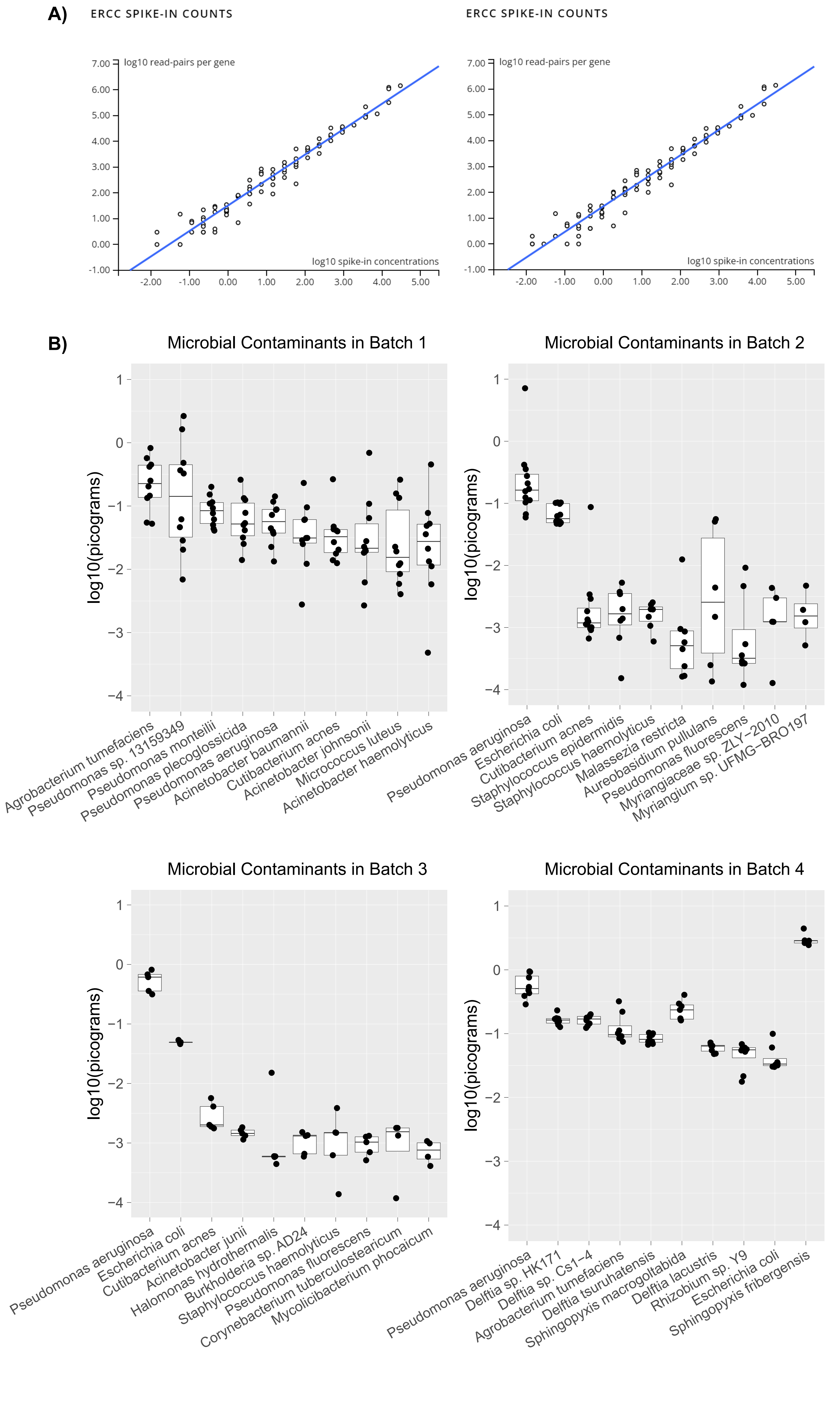

Legend: (A) 25pg of External RNA Consortium Controls (ERCC) was spiked into each sample prior to library preparation. Each aliquot of ERCC contains 92 different transcripts in different quantities. The scatterplots depict the input mass of each ERCC on the x-axis and the output transcript count of each ERCC on the y-mass. These data demonstrate log-linear amplification across 7 orders of magnitude, allowing for back-calculation of original RNA mass of each BAL sample and its microbiome constituents. **(B)** Water and HeLa "no-template" controls underwent RNA extraction, library preparation, and sequencing with each batch of samples. The mass of microbial contaminants in the negative controls of each batch was back-calculated using the ERCC equation. This box-whisker plot depicts the median and IQR of contaminants detected in each batch. To address ubiquitous contamination, the mass of each microbe in each BAL sample was reduced by the (mean + 2 SD) of what was observed in the control samples of the respective batch.

Univariate Causal Mediation Analysis for % of Association between Antibacterial Exposure & In-Hospital Mortality that is Mediated by Antibacterial Exposure-Induced Changes in BAL Microbes

ACTINOMYCES

```
glm(formula = Actinomyces ~ antibact_score_sum, family = "poisson",
    data = df)

Deviance Residuals:
    Min       1Q   Median       3Q      Max
-1.6095  -1.1789  -0.7188  -0.4633  16.7224

Coefficients:
                     Estimate Std. Error z value Pr(>|z|)
(Intercept)         0.2586731  0.1276646   2.026   0.0427 *
antibact_score_sum -0.0063539  0.0008531  -7.448 9.47e-14 ***
---
Signif. codes:  0 '***' 0.001 '**' 0.01 '*' 0.05 '.' 0.1 ' ' 1

(Dispersion parameter for poisson family taken to be 1)

    Null deviance: 882.52  on 228  degrees of freedom
Residual deviance: 799.64  on 227  degrees of freedom
AIC: Inf
```

```
glm(formula = outcome_dichot ~ Actinomyces + antibact_score_sum,
    family = binomial(link = "logit"), data = df)

Deviance Residuals:
    Min       1Q   Median       3Q      Max
-1.6707  -0.6628  -0.4252  -0.1132   2.4002

Coefficients:
                     Estimate Std. Error z value Pr(>|z|)
(Intercept)         -2.822691   0.380261  -7.423 1.14e-13 ***
Actinomyces        -12.738502  13.161541  -0.968    0.333
antibact_score_sum   0.005081   0.000988   5.143 2.71e-07 ***
---
Signif. codes:  0 '***' 0.001 '**' 0.01 '*' 0.05 '.' 0.1 ' ' 1

(Dispersion parameter for binomial family taken to be 1)

    Null deviance: 226.95  on 228  degrees of freedom
Residual deviance: 188.64  on 226  degrees of freedom
AIC: 194.64
```

```
Causal Mediation Analysis

Nonparametric Bootstrap Confidence Intervals with the BCa Method

                         Estimate 95% CI Lower 95% CI Upper p-value
ACME (control)            0.03994      0.00250         0.09   0.012 *
ACME (treated)            0.55227      0.01062         0.78   0.012 *
ADE (control)             0.19802      0.03430         0.76  <2e-16 ***
ADE (treated)             0.71036      0.42772         0.85  <2e-16 ***
Total Effect              0.75030      0.47643         0.87  <2e-16 ***
Prop. Mediated (control)  0.05323      0.00568         0.17   0.012 *
Prop. Mediated (treated)  0.73607      0.00724         0.95   0.012 *
ACME (average)            0.29611      0.00526         0.41   0.012 *
ADE (average)             0.45419      0.29833         0.77  <2e-16 ***
Prop. Mediated (average)  0.39465      0.00481         0.52   0.012 *
---
Signif. codes:  0 '***' 0.001 '**' 0.01 '*' 0.05 '.' 0.1 ' ' 1

Sample Size Used: 229


Simulations: 500
```

BACTEROIDES

```
glm(formula = Bacteroides ~ antibact_score_sum, family = "poisson", 
    data = df)

Deviance Residuals: 
    Min       1Q   Median       3Q      Max  
-0.2105  -0.1357  -0.1143  -0.1019   1.4559  

Coefficients:
                    Estimate Std. Error z value Pr(>|z|)    
(Intercept)        -5.380611   1.342982  -4.006 6.16e-05 ***
antibact_score_sum  0.001960   0.003449   0.568     0.57    
---
Signif. codes:  0 '***' 0.001 '**' 0.01 '*' 0.05 '.' 0.1 ' ' 1

(Dispersion parameter for poisson family taken to be 1)

    Null deviance: 11.976  on 228  degrees of freedom
Residual deviance: 11.674  on 227  degrees of freedom
AIC: Inf
```

```
glm(formula = outcome_dichot ~ Bacteroides + antibact_score_sum, 
    family = binomial(link = "logit"), data = df)

Deviance Residuals: 
    Min       1Q   Median       3Q      Max  
-1.6745  -0.6456  -0.4277  -0.3175   2.4546  

Coefficients:
                     Estimate Std. Error z value Pr(>|z|)    
(Intercept)        -2.9621110  0.3760765  -7.876 3.37e-15 ***
Bacteroides        -0.4859977  3.4035693  -0.143    0.886    
antibact_score_sum  0.0052716  0.0009875   5.339 9.37e-08 ***
---
Signif. codes:  0 '***' 0.001 '**' 0.01 '*' 0.05 '.' 0.1 ' ' 1

(Dispersion parameter for binomial family taken to be 1)

    Null deviance: 226.95  on 228  degrees of freedom
Residual deviance: 192.11  on 226  degrees of freedom
AIC: 198.11
```

```
Causal Mediation Analysis 

Nonparametric Bootstrap Confidence Intervals with the BCa Method

                          Estimate 95% CI Lower 95% CI Upper p-value    
ACME (control)           -0.000481     0.000152         0.13    0.58    
ACME (treated)           -0.002485    -0.011508         0.03    0.58    
ADE (control)             0.728699     0.461857         0.85  <2e-16 ***
ADE (treated)             0.726694     0.448437         0.85  <2e-16 ***
Total Effect              0.726214     0.457336         0.85  <2e-16 ***
Prop. Mediated (control) -0.000662     0.000216         0.22    0.58    
Prop. Mediated (treated) -0.003422    -0.009961         0.05    0.58    
ACME (average)           -0.001483    -0.001787         0.08    0.58    
ADE (average)             0.727696     0.455762         0.85  <2e-16 ***
Prop. Mediated (average) -0.002042    -0.001650         0.13    0.58    
---
Signif. codes:  0 '***' 0.001 '**' 0.01 '*' 0.05 '.' 0.1 ' ' 1

Sample Size Used: 229 


Simulations: 500 
```

CAPNOCYTOPHAGA

```
glm(formula = Capnocytophaga ~ antibact_score_sum, family = "poisson",
    data = df)

Deviance Residuals:
    Min       1Q   Median       3Q      Max
-0.6516  -0.5911  -0.5686  -0.5521  11.4695

Coefficients:
                     Estimate Std. Error z value Pr(>|z|)
(Intercept)        -1.8956720  0.2680530  -7.072 1.53e-12 ***
antibact_score_sum  0.0004315  0.0008209   0.526    0.599
---
Signif. codes:  0 '***' 0.001 '**' 0.01 '*' 0.05 '.' 0.1 ' ' 1

(Dispersion parameter for poisson family taken to be 1)

    Null deviance: 304.16  on 228  degrees of freedom
Residual deviance: 303.89  on 227  degrees of freedom
AIC: Inf
```

```
glm(formula = outcome_dichot ~ Capnocytophaga + antibact_score_sum,
    family = binomial(link = "logit"), data = df)

Deviance Residuals:
    Min       1Q   Median       3Q      Max
-1.6721  -0.6454  -0.4221  -0.3178   2.4539

Coefficients:
                     Estimate Std. Error z value Pr(>|z|)
(Intercept)        -2.9602206  0.3762064  -7.869 3.59e-15 ***
Capnocytophaga     -0.0207750  0.1544278  -0.135    0.893
antibact_score_sum  0.0052622  0.0009836   5.350 8.79e-08 ***
---
Signif. codes:  0 '***' 0.001 '**' 0.01 '*' 0.05 '.' 0.1 ' ' 1

(Dispersion parameter for binomial family taken to be 1)

    Null deviance: 226.95  on 228  degrees of freedom
Residual deviance: 192.11  on 226  degrees of freedom
AIC: 198.11
```

```
Causal Mediation Analysis

Nonparametric Bootstrap Confidence Intervals with the BCa Method

                          Estimate 95% CI Lower 95% CI Upper p-value
ACME (control)            3.37e-05    -2.03e-05         0.19     0.3
ACME (treated)            1.26e-04    -1.62e-04         0.38     0.3
ADE (control)             7.27e-01     3.67e-01         0.87  <2e-16 ***
ADE (treated)             7.28e-01     5.01e-01         0.87  <2e-16 ***
Total Effect              7.28e-01     5.02e-01         0.86  <2e-16 ***
Prop. Mediated (control)  4.64e-05    -2.39e-05         0.23     0.3
Prop. Mediated (treated)  1.73e-04    -2.02e-04         0.51     0.3
ACME (average)            8.00e-05    -1.44e-04         0.24     0.3
ADE (average)             7.28e-01     4.74e-01         0.86  <2e-16 ***
Prop. Mediated (average)  1.10e-04    -1.77e-04         0.30     0.3
---
Signif. codes:  0 '***' 0.001 '**' 0.01 '*' 0.05 '.' 0.1 ' ' 1

Sample Size Used: 229


Simulations:  500
```

FUSOBACTERIUM

```
glm(formula = Fusobacterium ~ antibact_score_sum, family = "poisson",
    data = df)

Deviance Residuals:
   Min      1Q  Median      3Q     Max
-2.474  -1.398  -0.495  -0.209  32.045

Coefficients:
                   Estimate Std. Error z value Pr(>|z|)
(Intercept)         1.11849    0.09616   11.63   <2e-16 ***
antibact_score_sum -0.01305    0.00118  -11.06   <2e-16 ***
---
Signif. codes:  0 '***' 0.001 '**' 0.01 '*' 0.05 '.' 0.1 ' ' 1

(Dispersion parameter for poisson family taken to be 1)

    Null deviance: 1637.6  on 228  degrees of freedom
Residual deviance: 1361.5  on 227  degrees of freedom
AIC: Inf
```

```
glm(formula = outcome_dichot ~ Fusobacterium + antibact_score_sum,
    family = binomial(link = "logit"), data = df)

Deviance Residuals:
    Min       1Q   Median       3Q      Max
-1.6742  -0.6531  -0.4179  -0.3239   2.4388

Coefficients:
                     Estimate Std. Error  z value Pr(>|z|)
(Intercept)        -2.9213715  0.3781588  -7.725 1.12e-14 ***
Fusobacterium      -1.9926571  3.4178250  -0.583     0.56
antibact_score_sum  0.0052182  0.0009865   5.290 1.23e-07 ***
---
Signif. codes:  0 '***' 0.001 '**' 0.01 '*' 0.05 '.' 0.1 ' ' 1

(Dispersion parameter for binomial family taken to be 1)

    Null deviance: 226.95  on 228  degrees of freedom
Residual deviance: 191.31  on 226  degrees of freedom
AIC: 197.31
```

```
Causal Mediation Analysis

Nonparametric Bootstrap Confidence Intervals with the BCa Method

                         Estimate 95% CI Lower 95% CI Upper p-value
ACME (control)           4.88e-02     1.69e-04         0.10    0.02 *
ACME (treated)           7.09e-01     5.95e-05         0.86    0.02 *
ADE (control)            6.68e-02     0.00e+00         0.84  <2e-16 ***
ADE (treated)            7.27e-01     4.80e-01         0.87  <2e-16 ***
Total Effect             7.76e-01     5.02e-01         0.89  <2e-16 ***
Prop. Mediated (control) 6.29e-02     7.14e-04         0.15    0.02 *
Prop. Mediated (treated) 9.14e-01     2.42e-05         1.00    0.02 *
ACME (average)           3.79e-01     2.76e-05         0.45    0.02 *
ADE (average)            3.97e-01     3.04e-01         0.85  <2e-16 ***
Prop. Mediated (average) 4.88e-01     0.00e+00         0.55    0.02 *
---
Signif. codes:  0 '***' 0.001 '**' 0.01 '*' 0.05 '.' 0.1 ' ' 1

Sample Size Used: 229


Simulations:  500
```

GEMELLA

```
glm(formula = Gemella ~ antibact_score_sum, family = "poisson",
    data = df)

Deviance Residuals:
    Min       1Q   Median       3Q      Max
-1.8604  -1.2058  -0.5866  -0.3158  23.1607

Coefficients:
                    Estimate Std. Error z value Pr(>|z|)
(Intercept)         0.548463   0.119560   4.587 4.49e-06 ***
antibact_score_sum -0.009364   0.001075  -8.708  < 2e-16 ***
---
Signif. codes:  0 '***' 0.001 '**' 0.01 '*' 0.05 '.' 0.1 ' ' 1

(Dispersion parameter for poisson family taken to be 1)

    Null deviance: 1026.10  on 228  degrees of freedom
Residual deviance:  887.43  on 227  degrees of freedom
AIC: Inf
```

```
glm(formula = outcome_dichot ~ Gemella + antibact_score_sum,
    family = binomial(link = "logit"), data = df)

Deviance Residuals:
     Min        1Q    Median        3Q       Max
-1.65796  -0.66823  -0.42913  -0.00255   2.38188

Coefficients:
                     Estimate Std. Error  z value Pr(>|z|)
(Intercept)         -2.776262   0.384005  -7.230 4.84e-13 ***
Gemella            -78.071071  98.525673  -0.792    0.428
antibact_score_sum   0.004984   0.001000   4.982 6.30e-07 ***
---
Signif. codes:  0 '***' 0.001 '**' 0.01 '*' 0.05 '.' 0.1 ' ' 1

(Dispersion parameter for binomial family taken to be 1)

    Null deviance: 226.95  on 228  degrees of freedom
Residual deviance: 186.91  on 226  degrees of freedom
AIC: 192.91
```

```
Causal Mediation Analysis

Nonparametric Bootstrap Confidence Intervals with the BCa Method

                         Estimate 95% CI Lower 95% CI Upper p-value
ACME (control)            0.04966      0.00319         0.11  <2e-16 ***
ACME (treated)            0.65273      0.01306         0.79  <2e-16 ***
ADE (control)             0.10880      0.00950         0.77  <2e-16 ***
ADE (treated)             0.71187      0.43264         0.85  <2e-16 ***
Total Effect              0.76153      0.49866         0.88  <2e-16 ***
Prop. Mediated (control)  0.06521      0.00678         0.22  <2e-16 ***
Prop. Mediated (treated)  0.85713      0.01673         0.99  <2e-16 ***
ACME (average)            0.35120      0.00686         0.42  <2e-16 ***
ADE (average)             0.41034      0.29770         0.80  <2e-16 ***
Prop. Mediated (average)  0.46117      0.00913         0.54  <2e-16 ***
---
Signif. codes:  0 '***' 0.001 '**' 0.01 '*' 0.05 '.' 0.1 ' ' 1

Sample Size Used: 229


Simulations:  500
```

GRANULICATELLA

```
glm(formula = Granulicatella ~ antibact_score_sum, family = "poisson",
    data = df)

Deviance Residuals:
    Min       1Q   Median       3Q      Max
-0.4315  -0.4028  -0.3613  -0.3323  10.4022

Coefficients:
                    Estimate Std. Error z value Pr(>|z|)
(Intercept)        -2.374085   0.382758  -6.203 5.55e-10 ***
antibact_score_sum -0.001291   0.001439  -0.897     0.37
---
Signif. codes:  0 '***' 0.001 '**' 0.01 '*' 0.05 '.' 0.1 ' ' 1

(Dispersion parameter for poisson family taken to be 1)

    Null deviance: 145.93  on 228  degrees of freedom
Residual deviance: 145.07  on 227  degrees of freedom
AIC: Inf
```

```
glm(formula = outcome_dichot ~ Granulicatella + antibact_score_sum,
    family = binomial(link = "logit"), data = df)

Deviance Residuals:
    Min       1Q   Median       3Q      Max
-1.6708  -0.6464  -0.4232  -0.3189   2.4511

Coefficients:
                     Estimate Std. Error z value Pr(>|z|)
(Intercept)        -2.9531770  0.3761074  -7.852 4.10e-15 ***
Granulicatella     -0.1699046  0.6161536  -0.276    0.783
antibact_score_sum  0.0052497  0.0009829   5.341 9.25e-08 ***
---
Signif. codes:  0 '***' 0.001 '**' 0.01 '*' 0.05 '.' 0.1 ' ' 1

(Dispersion parameter for binomial family taken to be 1)

    Null deviance: 226.95  on 228  degrees of freedom
Residual deviance: 191.97  on 226  degrees of freedom
AIC: 197.97
```

```
Causal Mediation Analysis

Nonparametric Bootstrap Confidence Intervals with the BCa Method

                          Estimate 95% CI Lower 95% CI Upper p-value
ACME (control)            0.000444    -0.020031         0.01    0.58
ACME (treated)            0.001909    -0.003250         0.24    0.58
ADE (control)             0.724832     0.485993         0.87  <2e-16 ***
ADE (treated)             0.726296     0.469151         0.87  <2e-16 ***
Total Effect              0.726741     0.469894         0.87  <2e-16 ***
Prop. Mediated (control)  0.000611    -0.034836         0.01    0.58
Prop. Mediated (treated)  0.002627    -0.005957         0.30    0.58
ACME (average)            0.001177    -0.006363         0.12    0.58
ADE (average)             0.725564     0.482772         0.87  <2e-16 ***
Prop. Mediated (average)  0.001619    -0.012552         0.15    0.58
---
Signif. codes:  0 '***' 0.001 '**' 0.01 '*' 0.05 '.' 0.1 ' ' 1

Sample Size Used: 229


Simulations:  500
```

HAEMOPHILUS

```
glm(formula = Haemophilus ~ antibact_score_sum, family = "poisson",
    data = df)

Deviance Residuals:
    Min       1Q   Median       3Q      Max
 -7.540   -4.861   -2.370   -1.321  101.805

Coefficients:
                     Estimate Std. Error z value Pr(>|z|)
(Intercept)         3.3473128  0.0293948  113.87   <2e-16 ***
antibact_score_sum -0.0092048  0.0002605  -35.34   <2e-16 ***
---
Signif. codes:  0 ‘***’ 0.001 ‘**’ 0.01 ‘*’ 0.05 ‘.’ 0.1 ‘ ’ 1

(Dispersion parameter for poisson family taken to be 1)

    Null deviance: 17523  on 228  degrees of freedom
Residual deviance: 15263  on 227  degrees of freedom
AIC: Inf
```

```
glm(formula = outcome_dichot ~ Haemophilus + antibact_score_sum,
    family = binomial(link = "logit"), data = df)

Deviance Residuals:
    Min       1Q   Median       3Q      Max
-1.6309  -0.6750  -0.4336   0.0000   2.3545

Coefficients:
                     Estimate Std. Error z value Pr(>|z|)
(Intercept)         -2.707163   0.387787  -6.981 2.93e-12 ***
Haemophilus        -23.640828  33.727910  -0.701    0.483
antibact_score_sum   0.004818   0.001004   4.800 1.58e-06 ***
---
Signif. codes:  0 ‘***’ 0.001 ‘**’ 0.01 ‘*’ 0.05 ‘.’ 0.1 ‘ ’ 1

(Dispersion parameter for binomial family taken to be 1)

    Null deviance: 226.95  on 228  degrees of freedom
Residual deviance: 186.17  on 226  degrees of freedom
AIC: 192.17
```

```
Causal Mediation Analysis

Nonparametric Bootstrap Confidence Intervals with the BCa Method

                         Estimate 95% CI Lower 95% CI Upper p-value
ACME (control)             0.0604       0.0240         0.11  <2e-16 ***
ACME (treated)             0.7324       0.3047         0.86  <2e-16 ***
ADE (control)              0.0000       0.0000         0.60       1
ADE (treated)              0.6721       0.4418         0.84  <2e-16 ***
Total Effect               0.7324       0.5119         0.88  <2e-16 ***
Prop. Mediated (control)   0.0824       0.0325         0.20  <2e-16 ***
Prop. Mediated (treated)   1.0000       0.2063         1.00  <2e-16 ***
ACME (average)             0.3964       0.1428         0.44  <2e-16 ***
ADE (average)              0.3360       0.2434         0.53  <2e-16 ***
Prop. Mediated (average)   0.5412       0.1263         0.55  <2e-16 ***
---
Signif. codes:  0 ‘***’ 0.001 ‘**’ 0.01 ‘*’ 0.05 ‘.’ 0.1 ‘ ’ 1

Sample Size Used: 229


Simulations: 500
```

NEISSERIA

```
glm(formula = Neisseria ~ antibact_score_sum, family = "poisson",
    data = df)

Deviance Residuals:
   Min      1Q  Median      3Q     Max
-3.062  -2.178  -1.250  -0.770  33.602

Coefficients:
                    Estimate Std. Error z value Pr(>|z|)
(Intercept)        1.5452842  0.0683089   22.62   <2e-16 ***
antibact_score_sum -0.0069521  0.0004857  -14.31   <2e-16 ***
---
Signif. codes:  0 '***' 0.001 '**' 0.01 '*' 0.05 '.' 0.1 ' ' 1

(Dispersion parameter for poisson family taken to be 1)

    Null deviance: 2789.9  on 228  degrees of freedom
Residual deviance: 2470.8  on 227  degrees of freedom
AIC: Inf
```

```
glm(formula = outcome_dichot ~ Neisseria + antibact_score_sum,
    family = binomial(link = "logit"), data = df)

Deviance Residuals:
    Min       1Q   Median       3Q      Max
-1.6563  -0.6650  -0.4304  -0.1822   2.4018

Coefficients:
                    Estimate Std. Error z value Pr(>|z|)
(Intercept)       -2.8267919  0.3800141  -7.439 1.02e-13 ***
Neisseria         -1.2738305  1.5274730  -0.834    0.404
antibact_score_sum  0.0050448  0.0009859   5.117 3.11e-07 ***
---
Signif. codes:  0 '***' 0.001 '**' 0.01 '*' 0.05 '.' 0.1 ' ' 1

(Dispersion parameter for binomial family taken to be 1)

    Null deviance: 226.95  on 228  degrees of freedom
Residual deviance: 189.24  on 226  degrees of freedom
AIC: 195.24
```

```
Causal Mediation Analysis

Nonparametric Bootstrap Confidence Intervals with the BCa Method

                         Estimate 95% CI Lower 95% CI Upper p-value
ACME (control)            0.05289      0.00825         0.09  <2e-16 ***
ACME (treated)            0.69212      0.03376         0.79  <2e-16 ***
ADE (control)             0.07070      0.00272         0.74  <2e-16 ***
ADE (treated)             0.70993      0.49161         0.85  <2e-16 ***
Total Effect              0.76282      0.54605         0.89  <2e-16 ***
Prop. Mediated (control)  0.06933      0.01327         0.14  <2e-16 ***
Prop. Mediated (treated)  0.90732      0.03890         1.00  <2e-16 ***
ACME (average)            0.37250      0.02013         0.42  <2e-16 ***
ADE (average)             0.39031      0.30957         0.76  <2e-16 ***
Prop. Mediated (average)  0.48833      0.02400         0.54  <2e-16 ***
---
Signif. codes:  0 '***' 0.001 '**' 0.01 '*' 0.05 '.' 0.1 ' ' 1

Sample Size Used: 229


Simulations: 500
```

PREVOTELLA

```
glm(formula = Prevotella ~ antibact_score_sum, family = "poisson",
    data = df)

Deviance Residuals:
   Min      1Q  Median      3Q     Max
-5.718  -3.933  -2.242  -1.271  79.841

Coefficients:
                     Estimate Std. Error z value Pr(>|z|)
(Intercept)         2.7939535  0.0374684   74.57   <2e-16 ***
antibact_score_sum -0.0078131  0.0002906  -26.89   <2e-16 ***
---
Signif. codes:  0 '***' 0.001 '**' 0.01 '*' 0.05 '.' 0.1 ' ' 1

(Dispersion parameter for poisson family taken to be 1)

    Null deviance: 10856.9  on 228  degrees of freedom
Residual deviance:  9662.8  on 227  degrees of freedom
AIC: Inf
```

```
glm(formula = outcome_dichot ~ Prevotella + antibact_score_sum,
    family = binomial(link = "logit"), data = df)

Deviance Residuals:
    Min       1Q   Median       3Q      Max
-1.6851  -0.6455  -0.4169  -0.3219   2.4424

Coefficients:
                     Estimate Std. Error z value Pr(>|z|)
(Intercept)        -2.9307360  0.3764834  -7.785 7.00e-15 ***
Prevotella         -0.0539406  0.0738593  -0.730    0.465
antibact_score_sum  0.0052616  0.0009858   5.338 9.42e-08 ***
---
Signif. codes:  0 '***' 0.001 '**' 0.01 '*' 0.05 '.' 0.1 ' ' 1

(Dispersion parameter for binomial family taken to be 1)

    Null deviance: 226.95  on 228  degrees of freedom
Residual deviance: 191.07  on 226  degrees of freedom
AIC: 197.07
```

```
Causal Mediation Analysis

Nonparametric Bootstrap Confidence Intervals with the BCa Method

                          Estimate 95% CI Lower 95% CI Upper p-value
ACME (control)             0.02837     -0.00213         0.13    0.34
ACME (treated)             0.18378     -0.00441         0.90    0.35
ADE (control)              0.57590      0.00000         0.79  <2e-16 ***
ADE (treated)              0.73131      0.14295         0.83  <2e-16 ***
Total Effect               0.75968      0.12397         0.85  <2e-16 ***
Prop. Mediated (control)   0.03735     -0.00413         0.20    0.34
Prop. Mediated (treated)   0.24192     -0.00986         1.00    0.35
ACME (average)             0.10608     -0.00272         0.47    0.35
ADE (average)              0.65361      0.14270         0.81  <2e-16 ***
Prop. Mediated (average)   0.13963     -0.00671         0.62    0.35
---
Signif. codes:  0 '***' 0.001 '**' 0.01 '*' 0.05 '.' 0.1 ' ' 1

Sample Size Used: 229


Simulations: 500
```

PORPHYROMONAS

```
glm(formula = Porphyromonas ~ antibact_score_sum, family = "poisson",
    data = df)

Deviance Residuals:
    Min       1Q   Median       3Q      Max
-0.1418  -0.1240  -0.1170  -0.1132   3.6648

Coefficients:
                     Estimate Std. Error z value Pr(>|z|)
(Intercept)        -5.0732188  1.2966706  -3.912 9.13e-05 ***
antibact_score_sum  0.0005912  0.0038972   0.152    0.879
---
Signif. codes:  0 '***' 0.001 '**' 0.01 '*' 0.05 '.' 0.1 ' ' 1

(Dispersion parameter for poisson family taken to be 1)

    Null deviance: 16.644  on 228  degrees of freedom
Residual deviance: 16.621  on 227  degrees of freedom
AIC: Inf
```

```
glm(formula = outcome_dichot ~ Porphyromonas + antibact_score_sum,
    family = binomial(link = "logit"), data = df)

Deviance Residuals:
    Min       1Q   Median       3Q      Max
-1.7090  -0.6506  -0.4324  -0.2558   2.5991

Coefficients:
                     Estimate Std. Error z value Pr(>|z|)
(Intercept)        -2.824e+00  3.764e-01  -7.501 6.31e-14 ***
Porphyromonas      -9.852e+02  7.902e+02  -1.247    0.212
antibact_score_sum  5.192e-03  9.850e-04   5.271 1.36e-07 ***
---
Signif. codes:  0 '***' 0.001 '**' 0.01 '*' 0.05 '.' 0.1 ' ' 1

(Dispersion parameter for binomial family taken to be 1)

    Null deviance: 226.95  on 228  degrees of freedom
Residual deviance: 188.04  on 226  degrees of freedom
AIC: 194.04
```

```
Causal Mediation Analysis

Nonparametric Bootstrap Confidence Intervals with the BCa Method

                          Estimate 95% CI Lower 95% CI Upper p-value
ACME (control)           -0.000979    -0.002398         0.00    0.44
ACME (treated)           -0.013813    -0.028713         0.01    0.44
ADE (control)             0.734758     0.538377         0.87  <2e-16 ***
ADE (treated)             0.721924     0.529938         0.87  <2e-16 ***
Total Effect              0.720945     0.527871         0.87  <2e-16 ***
Prop. Mediated (control) -0.001358    -0.004219         0.00    0.44
Prop. Mediated (treated) -0.019160    -0.041405         0.01    0.44
ACME (average)           -0.007396    -0.015371         0.00    0.44
ADE (average)             0.728341     0.534865         0.87  <2e-16 ***
Prop. Mediated (average) -0.010259    -0.022910         0.01    0.44
---
Signif. codes:  0 '***' 0.001 '**' 0.01 '*' 0.05 '.' 0.1 ' ' 1

Sample Size Used: 229


Simulations: 500
```

ROTHIA

```
glm(formula = Rothia ~ antibact_score_sum, family = "poisson",
    data = df)

Deviance Residuals:
    Min       1Q   Median       3Q      Max
-1.4291  -1.1364  -0.7791  -0.5539  14.6527

Coefficients:
                     Estimate Std. Error z value Pr(>|z|)
(Intercept)         0.0209155  0.1345777   0.155    0.876
antibact_score_sum -0.0044445  0.0007306  -6.083 1.18e-09 ***
---
Signif. codes:  0 '***' 0.001 '**' 0.01 '*' 0.05 '.' 0.1 ' ' 1

(Dispersion parameter for poisson family taken to be 1)

    Null deviance: 662.09  on 228  degrees of freedom
Residual deviance: 613.67  on 227  degrees of freedom
AIC: Inf
```

```
glm(formula = outcome_dichot ~ Rothia + antibact_score_sum, family = binomial(link = "logit"),
    data = df)

Deviance Residuals:
    Min       1Q   Median       3Q      Max
-1.6968  -0.6785  -0.4289   0.0000   2.3614

Coefficients:
                     Estimate Std. Error z value Pr(>|z|)
(Intercept)         -2.724547   0.391837  -6.953 3.57e-12 ***
Rothia             -32.708510  33.866858  -0.966    0.334
antibact_score_sum   0.005029   0.001021   4.924 8.48e-07 ***
---
Signif. codes:  0 '***' 0.001 '**' 0.01 '*' 0.05 '.' 0.1 ' ' 1

(Dispersion parameter for binomial family taken to be 1)

    Null deviance: 226.95  on 228  degrees of freedom
Residual deviance: 182.34  on 226  degrees of freedom
AIC: 188.34
```

```
Causal Mediation Analysis

Nonparametric Bootstrap Confidence Intervals with the BCa Method

                         Estimate 95% CI Lower 95% CI Upper p-value
ACME (control)            0.03950      0.00769         0.09  <2e-16 ***
ACME (treated)            0.50429      0.09298         0.73  <2e-16 ***
ADE (control)             0.23713      0.05112         0.61  <2e-16 ***
ADE (treated)             0.70192      0.44756         0.84  <2e-16 ***
Total Effect              0.74142      0.50530         0.87  <2e-16 ***
Prop. Mediated (control)  0.05328      0.01396         0.16  <2e-16 ***
Prop. Mediated (treated)  0.68016      0.12358         0.91  <2e-16 ***
ACME (average)            0.27190      0.04565         0.38  <2e-16 ***
ADE (average)             0.46953      0.31758         0.67  <2e-16 ***
Prop. Mediated (average)  0.36672      0.06687         0.50  <2e-16 ***
---
Signif. codes:  0 '***' 0.001 '**' 0.01 '*' 0.05 '.' 0.1 ' ' 1

Sample Size Used: 229


Simulations: 500
```

SCHAALIA

```
glm(formula = Schaalia ~ antibact_score_sum, family = "poisson",
    data = df)

Deviance Residuals:
   Min      1Q  Median      3Q     Max
-1.774  -1.400  -0.955  -0.669  19.028

Coefficients:
                     Estimate Std. Error z value Pr(>|z|)
(Intercept)         0.4537146  0.1102128   4.117 3.84e-05 ***
antibact_score_sum -0.0048870  0.0006286  -7.774 7.60e-15 ***
---
Signif. codes:  0 '***' 0.001 '**' 0.01 '*' 0.05 '.' 0.1 ' ' 1

(Dispersion parameter for poisson family taken to be 1)

    Null deviance: 1165.0  on 228  degrees of freedom
Residual deviance: 1083.5  on 227  degrees of freedom
AIC: Inf
```

```
glm(formula = outcome_dichot ~ Schaalia + antibact_score_sum,
    family = binomial(link = "logit"), data = df)

Deviance Residuals:
    Min       1Q   Median       3Q      Max
-1.6856  -0.6502  -0.4177  -0.3257   2.4336

Coefficients:
                     Estimate Std. Error z value Pr(>|z|)
(Intercept)        -2.9081705  0.3770688  -7.713 1.23e-14 ***
Schaalia           -0.5931053  0.7261879  -0.817    0.414
antibact_score_sum  0.0052338  0.0009863   5.307 1.12e-07 ***
---
Signif. codes:  0 '***' 0.001 '**' 0.01 '*' 0.05 '.' 0.1 ' ' 1

(Dispersion parameter for binomial family taken to be 1)

    Null deviance: 226.95  on 228  degrees of freedom
Residual deviance: 190.45  on 226  degrees of freedom
AIC: 196.45
```

```
Causal Mediation Analysis

Nonparametric Bootstrap Confidence Intervals with the BCa Method

                          Estimate 95% CI Lower 95% CI Upper p-value
ACME (control)             0.02521      0.00106         0.11   0.048 *
ACME (treated)             0.19811      0.00996         0.81   0.048 *
ADE (control)              0.55430      0.00473         0.78  <2e-16 ***
ADE (treated)              0.72719      0.42298         0.86  <2e-16 ***
Total Effect               0.75240      0.46186         0.87  <2e-16 ***
Prop. Mediated (control)   0.03351      0.00267         0.20   0.048 *
Prop. Mediated (treated)   0.26330      0.01093         1.00   0.048 *
ACME (average)             0.11166      0.00536         0.43   0.048 *
ADE (average)              0.64074      0.26881         0.82  <2e-16 ***
Prop. Mediated (average)   0.14840      0.00696         0.58   0.048 *
---
Signif. codes:  0 '***' 0.001 '**' 0.01 '*' 0.05 '.' 0.1 ' ' 1

Sample Size Used: 229


Simulations: 500
```

STAPHYLOCOCCUS

```
glm(formula = Staphylococcus ~ antibact_score_sum, family = "poisson",
    data = df)

Deviance Residuals:
   Min      1Q  Median      3Q     Max
-1.167  -1.143  -1.100  -1.059  22.777

Coefficients:
                     Estimate Std. Error z value Pr(>|z|)
(Intercept)        -0.3845185  0.1341187  -2.867  0.00414 **
antibact_score_sum -0.0004437  0.0004558  -0.974  0.33029
---
Signif. codes:  0 '***' 0.001 '**' 0.01 '*' 0.05 '.' 0.1 ' ' 1

(Dispersion parameter for poisson family taken to be 1)

    Null deviance: 1108.1  on 228  degrees of freedom
Residual deviance: 1107.1  on 227  degrees of freedom
AIC: Inf
```

```
glm(formula = outcome_dichot ~ Staphylococcus + antibact_score_sum,
    family = binomial(link = "logit"), data = df)

Deviance Residuals:
    Min       1Q   Median       3Q      Max
-1.7249  -0.6513  -0.4127  -0.3195   2.4497

Coefficients:
                    Estimate Std. Error z value Pr(>|z|)
(Intercept)        -2.949568   0.380204  -7.758 8.64e-15 ***
Staphylococcus     -0.774916   0.893756  -0.867    0.386
antibact_score_sum  0.005400   0.001006   5.368 7.95e-08 ***
---
Signif. codes:  0 '***' 0.001 '**' 0.01 '*' 0.05 '.' 0.1 ' ' 1

(Dispersion parameter for binomial family taken to be 1)

    Null deviance: 226.95  on 228  degrees of freedom
Residual deviance: 188.94  on 226  degrees of freedom
AIC: 194.94
```

```
Causal Mediation Analysis

Nonparametric Bootstrap Confidence Intervals with the BCa Method

                         Estimate 95% CI Lower 95% CI Upper p-value
ACME (control)            0.00622     -0.01696         0.03    0.71
ACME (treated)            0.05564     -0.18720         0.40    0.71
ADE (control)             0.64304      0.21468         0.83  <2e-16 ***
ADE (treated)             0.69246      0.20188         0.84  <2e-16 ***
Total Effect              0.69868      0.18496         0.85  <2e-16 ***
Prop. Mediated (control)  0.00891     -0.09223         0.03    0.71
Prop. Mediated (treated)  0.07964     -1.37601         0.40    0.71
ACME (average)            0.03093     -0.10742         0.21    0.70
ADE (average)             0.66775      0.24423         0.83  <2e-16 ***
Prop. Mediated (average)  0.04427     -0.72540         0.22    0.70
---
Signif. codes:  0 '***' 0.001 '**' 0.01 '*' 0.05 '.' 0.1 ' ' 1

Sample Size Used: 229


Simulations: 500
```

STREPTOCOCCUS

```
glm(formula = Streptococcus ~ antibact_score_sum, family = "poisson",
    data = df)

Deviance Residuals:
   Min      1Q  Median      3Q     Max
-6.375  -4.749  -2.975  -1.926  64.905

Coefficients:
                    Estimate Std. Error z value Pr(>|z|)
(Intercept)        3.0117050  0.0318812   94.47   <2e-16 ***
antibact_score_sum -0.0060072  0.0002054  -29.25   <2e-16 ***
---
Signif. codes:  0 '***' 0.001 '**' 0.01 '*' 0.05 '.' 0.1 ' ' 1

(Dispersion parameter for poisson family taken to be 1)

    Null deviance: 12796  on 228  degrees of freedom
Residual deviance: 11548  on 227  degrees of freedom
AIC: Inf
```

```
glm(formula = outcome_dichot ~ Streptococcus + antibact_score_sum,
    family = binomial(link = "logit"), data = df)

Deviance Residuals:
     Min        1Q    Median        3Q       Max
-1.63937  -0.66626  -0.43530  -0.00134   2.37988

Coefficients:
                    Estimate Std. Error z value Pr(>|z|)
(Intercept)       -2.6751751  0.3842762  -6.962 3.36e-12 ***
Streptococcus     -2.2760856  1.9583829  -1.162    0.245
antibact_score_sum  0.0048004  0.0009904   4.847 1.25e-06 ***
---
Signif. codes:  0 '***' 0.001 '**' 0.01 '*' 0.05 '.' 0.1 ' ' 1

(Dispersion parameter for binomial family taken to be 1)

    Null deviance: 226.95  on 228  degrees of freedom
Residual deviance: 185.68  on 226  degrees of freedom
AIC: 191.68
```

```
Causal Mediation Analysis

Nonparametric Bootstrap Confidence Intervals with the BCa Method

                         Estimate 95% CI Lower 95% CI Upper p-value
ACME (control)           5.51e-02     1.21e-02         0.11  <2e-16 ***
ACME (treated)           6.77e-01     9.14e-02         0.82  <2e-16 ***
ADE (control)            1.70e-10     1.30e-06         0.54  <2e-16 ***
ADE (treated)            6.22e-01     1.08e-01         0.81  <2e-16 ***
Total Effect             6.77e-01     9.61e-02         0.82  <2e-16 ***
Prop. Mediated (control) 8.14e-02     3.52e-02         0.25  <2e-16 ***
Prop. Mediated (treated) 1.00e+00     1.73e-01         1.00  <2e-16 ***
ACME (average)           3.66e-01     5.28e-02         0.43  <2e-16 ***
ADE (average)            3.11e-01     6.31e-02         0.42  <2e-16 ***
Prop. Mediated (average) 5.41e-01     2.27e-01         0.57  <2e-16 ***
---
Signif. codes:  0 '***' 0.001 '**' 0.01 '*' 0.05 '.' 0.1 ' ' 1

Sample Size Used: 229


Simulations: 500
```

VEILLONELLA

```
glm(formula = Veillonella ~ antibact_score_sum, family = "poisson", 
    data = df)

Deviance Residuals: 
   Min      1Q  Median      3Q     Max  
-1.519  -1.389  -1.205  -1.080  25.854  

Coefficients:
                     Estimate Std. Error z value Pr(>|z|)    
(Intercept)         0.1430844  0.1104797   1.295 0.195279    
antibact_score_sum -0.0015645  0.0004291  -3.646 0.000266 ***
---
Signif. codes:  0 ‘***’ 0.001 ‘**’ 0.01 ‘*’ 0.05 ‘.’ 0.1 ‘ ’ 1

(Dispersion parameter for poisson family taken to be 1)

    Null deviance: 1358.5  on 228  degrees of freedom
Residual deviance: 1344.1  on 227  degrees of freedom
AIC: Inf
```

```
glm(formula = outcome_dichot ~ Veillonella + antibact_score_sum, 
    family = binomial(link = "logit"), data = df)

Deviance Residuals: 
     Min        1Q    Median        3Q       Max  
-1.67204  -0.66484  -0.42885  -0.07462   2.38343  

Coefficients:
                     Estimate Std. Error z value Pr(>|z|)    
(Intercept)        -2.7801872  0.3811136  -7.295 2.99e-13 ***
Veillonella        -3.8874335  3.5626132  -1.091    0.275    
antibact_score_sum  0.0050296  0.0009902   5.080 3.78e-07 ***
---
Signif. codes:  0 ‘***’ 0.001 ‘**’ 0.01 ‘*’ 0.05 ‘.’ 0.1 ‘ ’ 1

(Dispersion parameter for binomial family taken to be 1)

    Null deviance: 226.95  on 228  degrees of freedom
Residual deviance: 187.06  on 226  degrees of freedom
AIC: 193.06
```

```
Causal Mediation Analysis 

Nonparametric Bootstrap Confidence Intervals with the BCa Method

                         Estimate 95% CI Lower 95% CI Upper p-value    
ACME (control)            0.01925      0.00336         0.07   0.056 .  
ACME (treated)            0.25069      0.00823         0.59   0.052 .  
ADE (control)             0.30141      0.10011         0.66  <2e-16 ***
ADE (treated)             0.53286      0.15514         0.80  <2e-16 ***
Total Effect              0.55211      0.16437         0.82  <2e-16 ***
Prop. Mediated (control)  0.03487      0.00608         0.14   0.056 .  
Prop. Mediated (treated)  0.45407     -0.07833         0.78   0.052 .  
ACME (average)            0.13497      0.00755         0.32   0.056 .  
ADE (average)             0.41713      0.14533         0.69  <2e-16 ***
Prop. Mediated (average)  0.24447     -0.04275         0.43   0.056 .  
---
Signif. codes:  0 ‘***’ 0.001 ‘**’ 0.01 ‘*’ 0.05 ‘.’ 0.1 ‘ ’ 1

Sample Size Used: 229 


Simulations: 500
```

Multivariable Causal Mediation Analysis for % of Association between Antibacterial Exposure & In-Hospital Mortality that is Mediated by Antibacterial Exposure-Induced Changes in BAL Microbes

ACTINOMYCES

```
glm(formula = Actinomyces ~ antibact_score_sum + anc + alc +
    spo2_support_dichot, family = "poisson", data = df)

Deviance Residuals:
    Min       1Q   Median       3Q      Max
-2.5402  -1.1653  -0.9259  -0.7566   4.4745

Coefficients:
                      Estimate Std. Error z value Pr(>|z|)
(Intercept)         -0.3042620  0.1805116  -1.686 0.091882 .
antibact_score_sum  -0.0019059  0.0005595  -3.407 0.000658 ***
anc                  0.0704659  0.0144390   4.880 1.06e-06 ***
alc                 -0.0426583  0.0677339  -0.630 0.528830
spo2_support_dichot -0.3274055  0.2022289  -1.619 0.105451
---
Signif. codes:  0 '***' 0.001 '**' 0.01 '*' 0.05 '.' 0.1 ' ' 1

(Dispersion parameter for poisson family taken to be 1)

    Null deviance: 486.05  on 214  degrees of freedom
Residual deviance: 446.47  on 210  degrees of freedom
  (14 observations deleted due to missingness)
AIC: Inf
```

```
glm(formula = outcome_dichot_new ~ Actinomyces + antibact_score_sum +
    anc + alc + spo2_support_dichot, family = binomial(link = "logit"),
    data = df)

Deviance Residuals:
    Min       1Q   Median       3Q      Max
-1.8192  -0.5893  -0.2445  -0.1393   2.8183

Coefficients:
                     Estimate Std. Error z value Pr(>|z|)
(Intercept)         -4.371811   0.721148  -6.062 1.34e-09 ***
Actinomyces         -0.181344   0.219462  -0.826    0.409
antibact_score_sum   0.004974   0.001211   4.106 4.02e-05 ***
anc                  0.048255   0.046955   1.028    0.304
alc                 -0.240207   0.321617  -0.747    0.455
spo2_support_dichot  2.169999   0.535985   4.049 5.15e-05 ***
---
Signif. codes:  0 '***' 0.001 '**' 0.01 '*' 0.05 '.' 0.1 ' ' 1

(Dispersion parameter for binomial family taken to be 1)

    Null deviance: 209.51  on 214  degrees of freedom
Residual deviance: 145.41  on 209  degrees of freedom
  (14 observations deleted due to missingness)
AIC: 157.41
```

```
Causal Mediation Analysis

Nonparametric Bootstrap Confidence Intervals with the BCa Method

                          Estimate 95% CI Lower 95% CI Upper p-value
ACME (control)            6.41e-03    -4.61e-04         0.06    0.32
ACME (treated)            2.59e-02    -4.16e-03         0.18    0.31
ADE (control)             5.26e-01     2.74e-01         0.78  <2e-16 ***
ADE (treated)             5.46e-01     3.26e-01         0.78  <2e-16 ***
Total Effect              5.52e-01     3.34e-01         0.78  <2e-16 ***
Prop. Mediated (control)  1.16e-02    -7.67e-05         0.18    0.32
Prop. Mediated (treated)  4.69e-02    -5.33e-03         0.53    0.31
ACME (average)            1.62e-02    -2.60e-03         0.11    0.31
ADE (average)             5.36e-01     3.03e-01         0.78  <2e-16 ***
Prop. Mediated (average)  2.93e-02    -3.11e-03         0.37    0.31
---
Signif. codes:  0 '***' 0.001 '**' 0.01 '*' 0.05 '.' 0.1 ' ' 1

Sample Size Used: 215
```

BACTEROIDES

```
glm(formula = Bacteroides ~ antibact_score_sum + anc + alc + 
    spo2_support_dichot, family = "poisson", data = df)

Deviance Residuals: 
    Min       1Q   Median       3Q      Max  
-1.8511  -0.8890  -0.7408  -0.6015   4.5668  

Coefficients:
                      Estimate Std. Error z value Pr(>|z|)    
(Intercept)         -0.9969892  0.2337780  -4.265    2e-05 ***
antibact_score_sum  -0.0020811  0.0007287  -2.856 0.004290 ** 
anc                  0.0690076  0.0180673   3.819 0.000134 ***
alc                  0.0588786  0.0662796   0.888 0.374360    
spo2_support_dichot  0.0150008  0.2545016   0.059 0.952999    
---
Signif. codes:  0 ‘***’ 0.001 ‘**’ 0.01 ‘*’ 0.05 ‘.’ 0.1 ‘ ’ 1

(Dispersion parameter for poisson family taken to be 1)

    Null deviance: 333.23  on 214  degrees of freedom
Residual deviance: 306.91  on 210  degrees of freedom
  (14 observations deleted due to missingness)
AIC: Inf
```

```
glm(formula = outcome_dichot_new ~ Bacteroides + antibact_score_sum + 
    anc + alc + spo2_support_dichot, family = binomial(link = "logit"), 
    data = df)

Deviance Residuals: 
    Min       1Q   Median       3Q      Max  
-1.8285  -0.5838  -0.2403  -0.1346   2.8396  

Coefficients:
                     Estimate Std. Error z value Pr(>|z|)    
(Intercept)         -4.429563   0.719477  -6.157 7.43e-10 ***
Bacteroides         -0.207033   0.281016  -0.737    0.461    
antibact_score_sum   0.005010   0.001211   4.137 3.52e-05 ***
anc                  0.044749   0.045772   0.978    0.328    
alc                 -0.222365   0.319802  -0.695    0.487    
spo2_support_dichot  2.222190   0.535951   4.146 3.38e-05 ***
---
Signif. codes:  0 ‘***’ 0.001 ‘**’ 0.01 ‘*’ 0.05 ‘.’ 0.1 ‘ ’ 1

(Dispersion parameter for binomial family taken to be 1)

    Null deviance: 209.51  on 214  degrees of freedom
Residual deviance: 145.59  on 209  degrees of freedom
  (14 observations deleted due to missingness)
AIC: 157.59
```

```
Causal Mediation Analysis 

Nonparametric Bootstrap Confidence Intervals with the BCa Method

                         Estimate 95% CI Lower 95% CI Upper p-value    
ACME (control)           0.005350    -0.003559         0.05    0.44    
ACME (treated)           0.018150    -0.000318         0.60    0.42    
ADE (control)            0.532940     0.240065         0.77  <2e-16 ***
ADE (treated)            0.545740     0.322808         0.81  <2e-16 ***
Total Effect             0.551090     0.323525         0.81  <2e-16 ***
Prop. Mediated (control) 0.009708    -0.004528         0.11    0.44    
Prop. Mediated (treated) 0.032934     0.000526         0.71    0.42    
ACME (average)           0.011750    -0.001612         0.30    0.43    
ADE (average)            0.539340     0.298423         0.79  <2e-16 ***
Prop. Mediated (average) 0.021321    -0.003158         0.38    0.43    
---
Signif. codes:  0 ‘***’ 0.001 ‘**’ 0.01 ‘*’ 0.05 ‘.’ 0.1 ‘ ’ 1

Sample Size Used: 215
```

CAPNOCYTOPHAGA

```
glm(formula = Capnocytophaga ~ antibact_score_sum + anc + alc + 
    spo2_support_dichot, family = "poisson", data = df)

Deviance Residuals: 
    Min       1Q   Median       3Q      Max  
-2.2798  -1.1262  -0.8748  -0.7064   3.7638  

Coefficients:
                      Estimate Std. Error z value Pr(>|z|)   
(Intercept)         -0.4945382  0.1872599  -2.641  0.00827 **
antibact_score_sum  -0.0010361  0.0005501  -1.883  0.05964 . 
anc                  0.0660307  0.0161078   4.099 4.14e-05 ***
alc                  0.0066351  0.0621959   0.107  0.91504   
spo2_support_dichot -0.5579014  0.2164932  -2.577  0.00997 **
---
Signif. codes:  0 '***' 0.001 '**' 0.01 '*' 0.05 '.' 0.1 ' ' 1

(Dispersion parameter for poisson family taken to be 1)

    Null deviance: 426.50  on 214  degrees of freedom
Residual deviance: 398.16  on 210  degrees of freedom
  (14 observations deleted due to missingness)
AIC: Inf
```

```
glm(formula = outcome_dichot_new ~ Capnocytophaga + antibact_score_sum + 
    anc + alc + spo2_support_dichot, family = binomial(link = "logit"), 
    data = df)

Deviance Residuals: 
    Min       1Q   Median       3Q      Max  
-1.8602  -0.5683  -0.2272  -0.0875   2.7976  

Coefficients:
                     Estimate Std. Error z value Pr(>|z|)    
(Intercept)         -4.434389   0.735969  -6.025 1.69e-09 ***
Capnocytophaga      -0.556936   0.282831  -1.969   0.0489 *  
antibact_score_sum   0.005214   0.001248   4.178 2.94e-05 ***
anc                  0.071832   0.046071   1.559   0.1190    
alc                 -0.272256   0.330806  -0.823   0.4105    
spo2_support_dichot  2.149100   0.540176   3.979 6.93e-05 ***
---
Signif. codes:  0 '***' 0.001 '**' 0.01 '*' 0.05 '.' 0.1 ' ' 1

(Dispersion parameter for binomial family taken to be 1)

    Null deviance: 209.51  on 214  degrees of freedom
Residual deviance: 141.24  on 209  degrees of freedom
  (14 observations deleted due to missingness)
AIC: 153.24
```

```
Causal Mediation Analysis 

Nonparametric Bootstrap Confidence Intervals with the BCa Method

                          Estimate 95% CI Lower 95% CI Upper p-value    
ACME (control)            0.009532    -0.001424         0.06    0.26    
ACME (treated)            0.044126     0.000711         0.43    0.21    
ADE (control)             0.522038     0.151629         0.76  <2e-16 ***
ADE (treated)             0.556633     0.283987         0.80  <2e-16 ***
Total Effect              0.566164     0.297982         0.81  <2e-16 ***
Prop. Mediated (control)  0.016835    -0.001273         0.18    0.26    
Prop. Mediated (treated)  0.077939     0.003287         0.72    0.21    
ACME (average)            0.026829     0.000522         0.23    0.22    
ADE (average)             0.539336     0.247962         0.79  <2e-16 ***
Prop. Mediated (average)  0.047387     0.001304         0.42    0.22    
---
Signif. codes:  0 '***' 0.001 '**' 0.01 '*' 0.05 '.' 0.1 ' ' 1

Sample Size Used: 215 
```

FUSOBACTERIUM

```
glm(formula = Fusobacterium ~ antibact_score_sum + anc + alc + 
    spo2_support_dichot, family = "poisson", data = df)

Deviance Residuals: 
    Min       1Q   Median       3Q      Max  
-1.6996  -1.1823  -0.9160  -0.7264   4.2873  

Coefficients:
                      Estimate Std. Error z value Pr(>|z|)    
(Intercept)         -0.1095536  0.1863008  -0.588    0.557    
antibact_score_sum  -0.0024941  0.0006088  -4.097 4.19e-05 ***
anc                  0.0194687  0.0189518   1.027    0.304    
alc                 -0.0118392  0.0680802  -0.174    0.862    
spo2_support_dichot -0.0417205  0.1996522  -0.209    0.834    
---
Signif. codes:  0 '***' 0.001 '**' 0.01 '*' 0.05 '.' 0.1 ' ' 1

(Dispersion parameter for poisson family taken to be 1)

    Null deviance: 448.54  on 214  degrees of freedom
Residual deviance: 424.67  on 210  degrees of freedom
  (14 observations deleted due to missingness)
AIC: Inf
```

```
glm(formula = outcome_dichot_new ~ Fusobacterium + antibact_score_sum + 
    anc + alc + spo2_support_dichot, family = binomial(link = "logit"), 
    data = df)

Deviance Residuals: 
     Min        1Q    Median        3Q       Max  
-1.60636  -0.56322  -0.22213  -0.08279   2.82949  

Coefficients:
                     Estimate Std. Error z value Pr(>|z|)    
(Intercept)         -4.458646   0.743773  -5.995 2.04e-09 ***
Fusobacterium       -0.561314   0.291693  -1.924   0.0543 .  
antibact_score_sum   0.005187   0.001249   4.153 3.29e-05 ***
anc                  0.054081   0.043307   1.249   0.2117    
alc                 -0.229060   0.317036  -0.723   0.4700    
spo2_support_dichot  2.264228   0.542984   4.170 3.05e-05 ***
---
Signif. codes:  0 '***' 0.001 '**' 0.01 '*' 0.05 '.' 0.1 ' ' 1

(Dispersion parameter for binomial family taken to be 1)

    Null deviance: 209.51  on 214  degrees of freedom
Residual deviance: 141.18  on 209  degrees of freedom
  (14 observations deleted due to missingness)
AIC: 153.18
```

```
Causal Mediation Analysis 

Nonparametric Bootstrap Confidence Intervals with the BCa Method

                         Estimate 95% CI Lower 95% CI Upper p-value    
ACME (control)            0.01598      0.00365         0.06   0.016 *  
ACME (treated)            0.07812      0.03336         0.53   0.012 *  
ADE (control)             0.50130      0.17346         0.72  <2e-16 ***
ADE (treated)             0.56344      0.35050         0.82  <2e-16 ***
Total Effect              0.57942      0.36648         0.84  <2e-16 ***
Prop. Mediated (control)  0.02757      0.00687         0.17   0.016 *  
Prop. Mediated (treated)  0.13483      0.05226         0.82   0.012 *  
ACME (average)            0.04705      0.01927         0.28   0.012 *  
ADE (average)             0.53237      0.31154         0.80  <2e-16 ***
Prop. Mediated (average)  0.08120      0.02930         0.47   0.012 *  
---
Signif. codes:  0 '***' 0.001 '**' 0.01 '*' 0.05 '.' 0.1 ' ' 1

Sample Size Used: 215 
```

GEMELLA

```
glm(formula = Gemella ~ antibact_score_sum + anc + alc + spo2_support_dichot,
    family = "poisson", data = df)

Deviance Residuals:
    Min       1Q   Median       3Q      Max
-2.0774  -1.0989  -0.7482  -0.5233   4.4668

Coefficients:
                      Estimate Std. Error z value Pr(>|z|)
(Intercept)          0.1996435  0.1932613   1.033    0.302
antibact_score_sum  -0.0042334  0.0007855  -5.389 7.08e-08 ***
anc                 -0.0377029  0.0289838  -1.301    0.193
alc                  0.0841806  0.0560363   1.502    0.133
spo2_support_dichot -0.3111068  0.2344684  -1.327    0.185
---
Signif. codes:  0 '***' 0.001 '**' 0.01 '*' 0.05 '.' 0.1 ' ' 1

(Dispersion parameter for poisson family taken to be 1)

    Null deviance: 433.93  on 214  degrees of freedom
Residual deviance: 377.11  on 210  degrees of freedom
  (14 observations deleted due to missingness)
AIC: Inf
```

```
glm(formula = outcome_dichot_new ~ Gemella + antibact_score_sum +
    anc + alc + spo2_support_dichot, family = binomial(link = "logit"),
    data = df)

Deviance Residuals:
    Min       1Q   Median       3Q      Max
-1.8262  -0.5903  -0.2300  -0.1207   2.8188

Coefficients:
                     Estimate Std. Error z value Pr(>|z|)
(Intercept)         -4.321462   0.724892  -5.962 2.50e-09 ***
Gemella             -0.323385   0.342480  -0.944    0.345
antibact_score_sum   0.004933   0.001224   4.031 5.55e-05 ***
anc                  0.034289   0.044189   0.776    0.438
alc                 -0.202657   0.315825  -0.642    0.521
spo2_support_dichot  2.165217   0.537226   4.030 5.57e-05 ***
---
Signif. codes:  0 '***' 0.001 '**' 0.01 '*' 0.05 '.' 0.1 ' ' 1

(Dispersion parameter for binomial family taken to be 1)

    Null deviance: 209.51  on 214  degrees of freedom
Residual deviance: 144.97  on 209  degrees of freedom
  (14 observations deleted due to missingness)
AIC: 156.97
```

```
Causal Mediation Analysis

Nonparametric Bootstrap Confidence Intervals with the BCa Method

                         Estimate 95% CI Lower 95% CI Upper p-value
ACME (control)            0.00920     -0.00111         0.06    0.27
ACME (treated)            0.05230     -0.00413         0.60    0.26
ADE (control)             0.50349      0.10324         0.75  <2e-16 ***
ADE (treated)             0.54659      0.32523         0.80  <2e-16 ***
Total Effect              0.55579      0.35229         0.80  <2e-16 ***
Prop. Mediated (control)  0.01656     -0.00130         0.16    0.27
Prop. Mediated (treated)  0.09410     -0.00988         0.84    0.26
ACME (average)            0.03075     -0.00322         0.31    0.26
ADE (average)             0.52504      0.24905         0.78  <2e-16 ***
Prop. Mediated (average)  0.05533     -0.00745         0.48    0.26
---
Signif. codes:  0 '***' 0.001 '**' 0.01 '*' 0.05 '.' 0.1 ' ' 1

Sample Size Used: 215
```

GRANULICATELLA

```
glm(formula = Granulicatella ~ antibact_score_sum + anc + alc + 
    spo2_support_dichot, family = "poisson", data = df)

Deviance Residuals: 
    Min       1Q   Median       3Q      Max  
-2.2395  -0.9856  -0.8069  -0.6752   4.7966  

Coefficients:
                      Estimate Std. Error z value Pr(>|z|)    
(Intercept)         -0.5013279  0.2114863  -2.370   0.0178 *  
antibact_score_sum  -0.0019075  0.0006334  -3.012   0.0026 ** 
anc                  0.0727945  0.0161354   4.511 6.44e-06 ***
alc                 -0.1539573  0.1028631  -1.497   0.1345    
spo2_support_dichot -0.3261099  0.2302744  -1.416   0.1567    
---
Signif. codes:  0 '***' 0.001 '**' 0.01 '*' 0.05 '.' 0.1 ' ' 1

(Dispersion parameter for poisson family taken to be 1)

    Null deviance: 383.83  on 214  degrees of freedom
Residual deviance: 352.53  on 210  degrees of freedom
  (14 observations deleted due to missingness)
AIC: Inf
```

```
glm(formula = outcome_dichot_new ~ Granulicatella + antibact_score_sum + 
    anc + alc + spo2_support_dichot, family = binomial(link = "logit"), 
    data = df)

Deviance Residuals: 
    Min       1Q   Median       3Q      Max  
-1.8319  -0.5858  -0.2394  -0.1457   2.8589  

Coefficients:
                     Estimate Std. Error z value Pr(>|z|)    
(Intercept)         -4.448779   0.730461  -6.090 1.13e-09 ***
Granulicatella      -0.018502   0.214080  -0.086    0.931    
antibact_score_sum   0.005070   0.001221   4.151 3.30e-05 ***
anc                  0.037456   0.046449   0.806    0.420    
alc                 -0.223045   0.321488  -0.694    0.488    
spo2_support_dichot  2.205175   0.538323   4.096 4.20e-05 ***
---
Signif. codes:  0 '***' 0.001 '**' 0.01 '*' 0.05 '.' 0.1 ' ' 1

(Dispersion parameter for binomial family taken to be 1)

    Null deviance: 209.51  on 214  degrees of freedom
Residual deviance: 146.17  on 209  degrees of freedom
  (14 observations deleted due to missingness)
AIC: 158.17
```

```
Causal Mediation Analysis 

Nonparametric Bootstrap Confidence Intervals with the BCa Method

                         Estimate 95% CI Lower 95% CI Upper p-value    
ACME (control)           0.000473    -0.012217         0.04    0.97    
ACME (treated)           0.001869    -0.018579         0.40    0.96    
ADE (control)            0.549144     0.236886         0.80  <2e-16 ***
ADE (treated)            0.550540     0.312316         0.82  <2e-16 ***
Total Effect             0.551013     0.325370         0.82  <2e-16 ***
Prop. Mediated (control) 0.000858    -0.020715         0.12    0.97    
Prop. Mediated (treated) 0.003392    -0.036057         0.71    0.96    
ACME (average)           0.001171    -0.015763         0.21    0.96    
ADE (average)            0.549842     0.288274         0.82  <2e-16 ***
Prop. Mediated (average) 0.002125    -0.031080         0.39    0.96    
---
Signif. codes:  0 '***' 0.001 '**' 0.01 '*' 0.05 '.' 0.1 ' ' 1

Sample Size Used: 215 
```

HAEMOPHILUS

```
glm(formula = Haemophilus ~ antibact_score_sum + anc + alc + 
    spo2_support_dichot, family = "poisson", data = df)

Deviance Residuals: 
    Min       1Q   Median       3Q      Max  
-2.6677  -1.3104  -0.9617  -0.5899   4.2170  

Coefficients:
                      Estimate Std. Error z value Pr(>|z|)    
(Intercept)          0.4407342  0.1506421   2.926  0.00344 ** 
antibact_score_sum  -0.0032805  0.0005658  -5.799 6.69e-09 ***
anc                  0.0088668  0.0179733   0.493  0.62178    
alc                  0.0652360  0.0427298   1.527  0.12683    
spo2_support_dichot -0.4128294  0.1820500  -2.268  0.02335 *  
---
Signif. codes:  0 '***' 0.001 '**' 0.01 '*' 0.05 '.' 0.1 ' ' 1

(Dispersion parameter for poisson family taken to be 1)

    Null deviance: 580.79  on 214  degrees of freedom
Residual deviance: 508.16  on 210  degrees of freedom
  (14 observations deleted due to missingness)
AIC: Inf
```

```
glm(formula = outcome_dichot_new ~ Haemophilus + antibact_score_sum + 
    anc + alc + spo2_support_dichot, family = binomial(link = "logit"), 
    data = df)

Deviance Residuals: 
    Min       1Q   Median       3Q      Max  
-1.8432  -0.5833  -0.2387  -0.1115   2.8209  

Coefficients:
                     Estimate Std. Error z value Pr(>|z|)    
(Intercept)         -4.318446   0.727358  -5.937 2.90e-09 ***
Haemophilus         -0.208724   0.214967  -0.971    0.332    
antibact_score_sum   0.004953   0.001216   4.075 4.61e-05 ***
anc                  0.035267   0.043209   0.816    0.414    
alc                 -0.223860   0.320041  -0.699    0.484    
spo2_support_dichot  2.185611   0.536409   4.075 4.61e-05 ***
---
Signif. codes:  0 '***' 0.001 '**' 0.01 '*' 0.05 '.' 0.1 ' ' 1

(Dispersion parameter for binomial family taken to be 1)

    Null deviance: 209.51  on 214  degrees of freedom
Residual deviance: 145.09  on 209  degrees of freedom
  (14 observations deleted due to missingness)
AIC: 157.09
```

```
Causal Mediation Analysis 

Nonparametric Bootstrap Confidence Intervals with the BCa Method

                          Estimate 95% CI Lower 95% CI Upper p-value    
ACME (control)            0.009577    -0.001227         0.05     0.2    
ACME (treated)            0.052905    -0.015619         0.33     0.2    
ADE (control)             0.504113     0.223917         0.75  <2e-16 ***
ADE (treated)             0.547441     0.316898         0.80  <2e-16 ***
Total Effect              0.557018     0.334484         0.80  <2e-16 ***
Prop. Mediated (control)  0.017194     0.000155         0.14     0.2    
Prop. Mediated (treated)  0.094979    -0.023128         0.69     0.2    
ACME (average)            0.031241    -0.008641         0.19     0.2    
ADE (average)             0.525777     0.280833         0.77  <2e-16 ***
Prop. Mediated (average)  0.056087    -0.015360         0.38     0.2    
---
Signif. codes:  0 '***' 0.001 '**' 0.01 '*' 0.05 '.' 0.1 ' ' 1

Sample Size Used: 215 
```

NEISSERIA

```
glm(formula = Neisseria ~ antibact_score_sum + anc + alc + spo2_support_dichot,
    family = "poisson", data = df)

Deviance Residuals:
    Min       1Q   Median       3Q      Max
-1.4796  -1.1674  -0.9811  -0.7735   4.5752

Coefficients:
                     Estimate Std. Error z value Pr(>|z|)
(Intercept)          0.117509   0.191944   0.612 0.540404
antibact_score_sum  -0.002211   0.000596  -3.710 0.000207 ***
anc                 -0.051065   0.027983  -1.825 0.068026 .
alc                  0.001468   0.074603   0.020 0.984300
spo2_support_dichot -0.086345   0.198686  -0.435 0.663865
---
Signif. codes:  0 '***' 0.001 '**' 0.01 '*' 0.05 '.' 0.1 ' ' 1

(Dispersion parameter for poisson family taken to be 1)

    Null deviance: 518.48  on 214  degrees of freedom
Residual deviance: 497.17  on 210  degrees of freedom
  (14 observations deleted due to missingness)
AIC: Inf
```

```
glm(formula = outcome_dichot_new ~ Neisseria + antibact_score_sum +
    anc + alc + spo2_support_dichot, family = binomial(link = "logit"),
    data = df)

Deviance Residuals:
    Min       1Q   Median       3Q      Max
-1.8363  -0.5919  -0.2321  -0.1459   2.8593

Coefficients:
                     Estimate Std. Error z value Pr(>|z|)
(Intercept)         -4.442151   0.726958  -6.111 9.93e-10 ***
Neisseria           -0.027780   0.163749  -0.170    0.865
antibact_score_sum   0.005066   0.001215   4.169 3.06e-05 ***
anc                  0.035854   0.044421   0.807    0.420
alc                 -0.221405   0.320622  -0.691    0.490
spo2_support_dichot  2.211013   0.534886   4.134 3.57e-05 ***
---
Signif. codes:  0 '***' 0.001 '**' 0.01 '*' 0.05 '.' 0.1 ' ' 1

(Dispersion parameter for binomial family taken to be 1)

    Null deviance: 209.51  on 214  degrees of freedom
Residual deviance: 146.15  on 209  degrees of freedom
  (14 observations deleted due to missingness)
AIC: 158.15
```

```
Causal Mediation Analysis

Nonparametric Bootstrap Confidence Intervals with the BCa Method

                         Estimate 95% CI Lower 95% CI Upper p-value
ACME (control)            0.00105     -0.00299         0.05     0.8
ACME (treated)            0.00419     -0.00830         0.44     0.8
ADE (control)             0.54710      0.27281         0.81  <2e-16 ***
ADE (treated)             0.55023      0.33580         0.81  <2e-16 ***
Total Effect              0.55128      0.34991         0.82  <2e-16 ***
Prop. Mediated (control)  0.00191     -0.00432         0.13     0.8
Prop. Mediated (treated)  0.00759     -0.01414         0.81     0.8
ACME (average)            0.00262     -0.00655         0.24     0.8
ADE (average)             0.54866      0.31270         0.81  <2e-16 ***
Prop. Mediated (average)  0.00475     -0.00959         0.47     0.8
---
Signif. codes:  0 '***' 0.001 '**' 0.01 '*' 0.05 '.' 0.1 ' ' 1

Sample Size Used: 215
```

PREVOTELLA

```
glm(formula = Prevotella ~ antibact_score_sum + anc + alc + spo2_support_dichot,
    family = "poisson", data = df)

Deviance Residuals:
    Min       1Q   Median       3Q      Max
-2.8740  -1.5173  -1.1815   0.7395   4.3353

Coefficients:
                      Estimate Std. Error z value Pr(>|z|)
(Intercept)          0.3276594  0.1349832   2.427   0.0152 *
antibact_score_sum  -0.0022833  0.0004224  -5.405 6.48e-08 ***
anc                  0.0530663  0.0110467   4.804 1.56e-06 ***
alc                 -0.0275769  0.0501775  -0.550   0.5826
spo2_support_dichot  0.0388659  0.1432468   0.271   0.7861
---
Signif. codes:  0 '***' 0.001 '**' 0.01 '*' 0.05 '.' 0.1 ' ' 1

(Dispersion parameter for poisson family taken to be 1)

    Null deviance: 625.06  on 214  degrees of freedom
Residual deviance: 566.42  on 210  degrees of freedom
  (14 observations deleted due to missingness)
AIC: Inf
```

```
glm(formula = outcome_dichot_new ~ Prevotella + antibact_score_sum +
    anc + alc + spo2_support_dichot, family = binomial(link = "logit"),
    data = df)

Deviance Residuals:
    Min       1Q   Median       3Q      Max
-1.7878  -0.5337  -0.2385  -0.1002   2.7080

Coefficients:
                     Estimate Std. Error z value Pr(>|z|)
(Intercept)         -4.244685   0.726376  -5.844 5.11e-09 ***
Prevotella          -0.405554   0.183988  -2.204 0.027507 *
antibact_score_sum   0.004624   0.001234   3.748 0.000178 ***
anc                  0.082603   0.050423   1.638 0.101382
alc                 -0.215016   0.324902  -0.662 0.508108
spo2_support_dichot  2.228336   0.539387   4.131 3.61e-05 ***
---
Signif. codes:  0 '***' 0.001 '**' 0.01 '*' 0.05 '.' 0.1 ' ' 1

(Dispersion parameter for binomial family taken to be 1)

    Null deviance: 209.51  on 214  degrees of freedom
Residual deviance: 139.75  on 209  degrees of freedom
  (14 observations deleted due to missingness)
AIC: 151.75
```

```
Causal Mediation Analysis

Nonparametric Bootstrap Confidence Intervals with the BCa Method

                         Estimate 95% CI Lower 95% CI Upper p-value
ACME (control)            0.02889      0.00944         0.09   0.004 **
ACME (treated)            0.09753      0.03418         0.30   0.004 **
ADE (control)             0.44282      0.19249         0.70  <2e-16 ***
ADE (treated)             0.51146      0.25595         0.77  <2e-16 ***
Total Effect              0.54035      0.30074         0.79  <2e-16 ***
Prop. Mediated (control)  0.05347      0.01824         0.23   0.004 **
Prop. Mediated (treated)  0.18049      0.06236         0.56   0.004 **
ACME (average)            0.06321      0.02012         0.18   0.004 **
ADE (average)             0.47714      0.21319         0.71  <2e-16 ***
Prop. Mediated (average)  0.11698      0.03748         0.39   0.004 **
---
Signif. codes:  0 '***' 0.001 '**' 0.01 '*' 0.05 '.' 0.1 ' ' 1

Sample Size Used: 215
```

PORPHYROMONAS

```
glm(formula = Porphyromonas ~ antibact_score_sum + anc + alc +
    spo2_support_dichot, family = "poisson", data = df)

Deviance Residuals:
    Min       1Q   Median       3Q      Max
-1.1918  -0.7037  -0.5856  -0.4472   4.5094

Coefficients:
                     Estimate Std. Error z value Pr(>|z|)
(Intercept)         -0.830984   0.330145  -2.517   0.0118 *
antibact_score_sum  -0.001573   0.000963  -1.633   0.1025
anc                 -0.168041   0.070614  -2.380   0.0173 *
alc                  0.191755   0.101201   1.895   0.0581 .
spo2_support_dichot -0.200243   0.340413  -0.588   0.5564
---
Signif. codes:  0 '***' 0.001 '**' 0.01 '*' 0.05 '.' 0.1 ' ' 1

(Dispersion parameter for poisson family taken to be 1)

    Null deviance: 224.69  on 214  degrees of freedom
Residual deviance: 210.79  on 210  degrees of freedom
  (14 observations deleted due to missingness)
AIC: Inf
```

```
glm(formula = outcome_dichot_new ~ Porphyromonas + antibact_score_sum +
    anc + alc + spo2_support_dichot, family = binomial(link = "logit"),
    data = df)

Deviance Residuals:
    Min       1Q   Median       3Q      Max
-1.8263  -0.5952  -0.2366  -0.1503   2.8419

Coefficients:
                     Estimate Std. Error z value Pr(>|z|)
(Intercept)         -4.397996   0.722500  -6.087 1.15e-09 ***
Porphyromonas       -0.268528   0.463616  -0.579    0.562
antibact_score_sum   0.004987   0.001215   4.105 4.04e-05 ***
anc                  0.034309   0.044166   0.777    0.437
alc                 -0.194549   0.319370  -0.609    0.542
spo2_support_dichot  2.202575   0.535191   4.115 3.86e-05 ***
---
Signif. codes:  0 '***' 0.001 '**' 0.01 '*' 0.05 '.' 0.1 ' ' 1

(Dispersion parameter for binomial family taken to be 1)

    Null deviance: 209.51  on 214  degrees of freedom
Residual deviance: 145.78  on 209  degrees of freedom
  (14 observations deleted due to missingness)
AIC: 157.78
```

```
Causal Mediation Analysis

Nonparametric Bootstrap Confidence Intervals with the BCa Method

                         Estimate 95% CI Lower 95% CI Upper p-value
ACME (control)            0.00159     -0.00136         0.03    0.58
ACME (treated)            0.00989     -0.00255         0.31    0.59
ADE (control)             0.53652      0.26525         0.74  <2e-16 ***
ADE (treated)             0.54482      0.30403         0.78  <2e-16 ***
Total Effect              0.54641      0.30676         0.78  <2e-16 ***
Prop. Mediated (control)  0.00291     -0.00171         0.07    0.58
Prop. Mediated (treated)  0.01810     -0.00573         0.49    0.59
ACME (average)            0.00574     -0.00166         0.16    0.60
ADE (average)             0.54067      0.30384         0.76  <2e-16 ***
Prop. Mediated (average)  0.01051     -0.00427         0.27    0.60
---
Signif. codes:  0 '***' 0.001 '**' 0.01 '*' 0.05 '.' 0.1 ' ' 1

Sample Size Used: 215
```

ROTHIA

```
glm(formula = Rothia ~ antibact_score_sum + anc + alc + spo2_support_dichot,
    family = "poisson", data = df)

Deviance Residuals:
    Min       1Q   Median       3Q      Max
-2.1427  -1.3353  -1.0337  -0.7414   4.5202

Coefficients:
                      Estimate Std. Error z value Pr(>|z|)
(Intercept)          0.4588536  0.1596564   2.874  0.00405 **
antibact_score_sum  -0.0026862  0.0005143  -5.223 1.76e-07 ***
anc                  0.0302731  0.0150244   2.015  0.04391 *
alc                 -0.2336615  0.0900961  -2.593  0.00950 **
spo2_support_dichot -0.2210217  0.1693337  -1.305  0.19181
---
Signif. codes:  0 '***' 0.001 '**' 0.01 '*' 0.05 '.' 0.1 ' ' 1

(Dispersion parameter for poisson family taken to be 1)

    Null deviance: 585.76  on 214  degrees of freedom
Residual deviance: 539.89  on 210  degrees of freedom
  (14 observations deleted due to missingness)
AIC: Inf
```

```
glm(formula = outcome_dichot_new ~ Rothia + antibact_score_sum +
    anc + alc + spo2_support_dichot, family = binomial(link = "logit"),
    data = df)

Deviance Residuals:
     Min        1Q    Median        3Q       Max
-1.58801  -0.56424  -0.24003  -0.08246   2.84513

Coefficients:
                     Estimate Std. Error z value Pr(>|z|)
(Intercept)         -4.257047   0.741856  -5.738 9.56e-09 ***
Rothia              -0.460994   0.227515  -2.026   0.0427 *
antibact_score_sum   0.004847   0.001246   3.890   0.0001 ***
anc                  0.057298   0.050866   1.126   0.2600
alc                 -0.297465   0.329224  -0.904   0.3662
spo2_support_dichot  2.229085   0.539631   4.131 3.62e-05 ***
---
Signif. codes:  0 '***' 0.001 '**' 0.01 '*' 0.05 '.' 0.1 ' ' 1

(Dispersion parameter for binomial family taken to be 1)

    Null deviance: 209.51  on 214  degrees of freedom
Residual deviance: 140.07  on 209  degrees of freedom
  (14 observations deleted due to missingness)
AIC: 152.07
```

```
Causal Mediation Analysis

Nonparametric Bootstrap Confidence Intervals with the BCa Method

                         Estimate 95% CI Lower 95% CI Upper p-value
ACME (control)            0.02146      0.00559         0.07   0.008 **
ACME (treated)            0.09565      0.04877         0.63   0.008 **
ADE (control)             0.45487      0.09482         0.67  <2e-16 ***
ADE (treated)             0.52906      0.30890         0.78  <2e-16 ***
Total Effect              0.55052      0.34340         0.79  <2e-16 ***
Prop. Mediated (control)  0.03898      0.01026         0.19   0.008 **
Prop. Mediated (treated)  0.17374      0.10074         0.89   0.008 **
ACME (average)            0.05855      0.03004         0.32   0.008 **
ADE (average)             0.49197      0.23755         0.74  <2e-16 ***
Prop. Mediated (average)  0.10636      0.05682         0.50   0.008 **
---
Signif. codes:  0 '***' 0.001 '**' 0.01 '*' 0.05 '.' 0.1 ' ' 1

Sample Size Used: 215
```

SCHAALIA

```
glm(formula = Schaalia ~ antibact_score_sum + anc + alc + spo2_support_dichot,
    family = "poisson", data = df)

Deviance Residuals:
    Min       1Q   Median       3Q      Max
-3.1735  -1.1796  -0.9622  -0.7361   4.4510

Coefficients:
                      Estimate Std. Error z value Pr(>|z|)
(Intercept)         -0.0283758  0.1677326  -0.169   0.8657
antibact_score_sum  -0.0020184  0.0005042  -4.004 6.24e-05 ***
anc                  0.0844621  0.0121752   6.937 4.00e-12 ***
alc                 -0.1883089  0.0861880  -2.185   0.0289 *
spo2_support_dichot -0.4241483  0.1851731  -2.291   0.0220 *
---
Signif. codes:  0 '***' 0.001 '**' 0.01 '*' 0.05 '.' 0.1 ' ' 1

(Dispersion parameter for poisson family taken to be 1)

    Null deviance: 535.65  on 214  degrees of freedom
Residual deviance: 469.06  on 210  degrees of freedom
  (14 observations deleted due to missingness)
AIC: Inf
```

```
glm(formula = outcome_dichot_new ~ Schaalia + antibact_score_sum +
    anc + alc + spo2_support_dichot, family = binomial(link = "logit"),
    data = df)

Deviance Residuals:
     Min        1Q    Median        3Q       Max
-1.75434  -0.58690  -0.23110  -0.07853   2.72582

Coefficients:
                     Estimate Std. Error z value Pr(>|z|)
(Intercept)         -4.375597   0.732830  -5.971 2.36e-09 ***
Schaalia            -0.582979   0.300232  -1.942   0.0522 .
antibact_score_sum   0.004804   0.001228   3.913 9.13e-05 ***
anc                  0.105422   0.057606   1.830   0.0672 .
alc                 -0.290555   0.322496  -0.901   0.3676
spo2_support_dichot  2.152549   0.539488   3.990 6.61e-05 ***
---
Signif. codes:  0 '***' 0.001 '**' 0.01 '*' 0.05 '.' 0.1 ' ' 1

(Dispersion parameter for binomial family taken to be 1)

    Null deviance: 209.51  on 214  degrees of freedom
Residual deviance: 140.50  on 209  degrees of freedom
  (14 observations deleted due to missingness)
AIC: 152.5
```

```
Causal Mediation Analysis

Nonparametric Bootstrap Confidence Intervals with the BCa Method

                         Estimate 95% CI Lower 95% CI Upper p-value
ACME (control)            0.02441      0.00385         0.07   0.020 *
ACME (treated)            0.08047      0.03823         0.48   0.008 **
ADE (control)             0.46909      0.12885         0.73  <2e-16 ***
ADE (treated)             0.52515      0.28364         0.81  <2e-16 ***
Total Effect              0.54956      0.32275         0.82  <2e-16 ***
Prop. Mediated (control)  0.04442      0.00833         0.22   0.020 *
Prop. Mediated (treated)  0.14642      0.07705         0.84   0.008 **
ACME (average)            0.05244      0.02112         0.25   0.008 **
ADE (average)             0.49712      0.23132         0.77  <2e-16 ***
Prop. Mediated (average)  0.09542      0.04264         0.48   0.008 **
---
Signif. codes:  0 '***' 0.001 '**' 0.01 '*' 0.05 '.' 0.1 ' ' 1

Sample Size Used: 215
```

STAPHYLOCOCCUS

```
glm(formula = Staphylococcus ~ antibact_score_sum + anc + alc + 
    spo2_support_dichot, family = "poisson", data = df)

Deviance Residuals: 
    Min       1Q   Median       3Q      Max  
-1.4087  -1.2284  -1.0335  -0.9658   4.7447  

Coefficients:
                      Estimate Std. Error z value Pr(>|z|)    
(Intercept)         -0.7104152  0.2010558  -3.533  0.00041 ***
antibact_score_sum   0.0003464  0.0004623   0.749  0.45368    
anc                 -0.0264192  0.0218691  -1.208  0.22702    
alc                  0.0665013  0.0714957   0.930  0.35230    
spo2_support_dichot  0.4228568  0.1844125   2.293  0.02185 *  
---
Signif. codes:  0 '***' 0.001 '**' 0.01 '*' 0.05 '.' 0.1 ' ' 1

(Dispersion parameter for poisson family taken to be 1)

    Null deviance: 513.93  on 214  degrees of freedom
Residual deviance: 505.76  on 210  degrees of freedom
  (14 observations deleted due to missingness)
AIC: Inf
```

```
glm(formula = outcome_dichot_new ~ Staphylococcus + antibact_score_sum + 
    anc + alc + spo2_support_dichot, family = binomial(link = "logit"), 
    data = df)

Deviance Residuals: 
    Min       1Q   Median       3Q      Max  
-1.7926  -0.5697  -0.2371  -0.1353   2.7311  

Coefficients:
                     Estimate Std. Error z value Pr(>|z|)    
(Intercept)         -4.602171   0.747523  -6.157 7.43e-10 ***
Staphylococcus       0.140217   0.119771   1.171    0.242    
antibact_score_sum   0.005152   0.001218   4.229 2.35e-05 ***
anc                  0.041232   0.045234   0.912    0.362    
alc                 -0.258338   0.328638  -0.786    0.432    
spo2_support_dichot  2.210127   0.539518   4.096 4.19e-05 ***
---
Signif. codes:  0 '***' 0.001 '**' 0.01 '*' 0.05 '.' 0.1 ' ' 1

(Dispersion parameter for binomial family taken to be 1)

    Null deviance: 209.51  on 214  degrees of freedom
Residual deviance: 144.86  on 209  degrees of freedom
  (14 observations deleted due to missingness)
AIC: 156.86
```

```
Causal Mediation Analysis 

Nonparametric Bootstrap Confidence Intervals with the BCa Method

                         Estimate 95% CI Lower 95% CI Upper p-value    
ACME (control)           0.000262    -0.003464         0.04    0.83    
ACME (treated)           0.004468    -0.008148         0.08    0.84    
ADE (control)            0.546906     0.322563         0.82  <2e-16 ***
ADE (treated)            0.551112     0.329101         0.82  <2e-16 ***
Total Effect             0.551374     0.330939         0.83  <2e-16 ***
Prop. Mediated (control) 0.000476    -0.005218         0.10    0.83    
Prop. Mediated (treated) 0.008103    -0.014243         0.16    0.84    
ACME (average)           0.002365    -0.005614         0.05    0.85    
ADE (average)            0.549009     0.325899         0.83  <2e-16 ***
Prop. Mediated (average) 0.004289    -0.010982         0.13    0.85    
---
Signif. codes:  0 '***' 0.001 '**' 0.01 '*' 0.05 '.' 0.1 ' ' 1

Sample Size Used: 215 
```

STREPTOCOCCUS

```
glm(formula = Streptococcus ~ antibact_score_sum + anc + alc +
    spo2_support_dichot, family = "poisson", data = df)

Deviance Residuals:
    Min       1Q   Median       3Q      Max
-2.2799  -1.5188  -1.0791  -0.6236   4.3667

Coefficients:
                      Estimate Std. Error z value Pr(>|z|)
(Intercept)          0.9518762  0.1342130   7.092 1.32e-12 ***
antibact_score_sum  -0.0033630  0.0004765  -7.058 1.68e-12 ***
anc                  0.0104208  0.0148066   0.704   0.4816
alc                 -0.1729957  0.0684986  -2.526   0.0116 *
spo2_support_dichot -0.3654100  0.1509802  -2.420   0.0155 *
---
Signif. codes:  0 '***' 0.001 '**' 0.01 '*' 0.05 '.' 0.1 ' ' 1

(Dispersion parameter for poisson family taken to be 1)

    Null deviance: 708.94  on 214  degrees of freedom
Residual deviance: 625.46  on 210  degrees of freedom
  (14 observations deleted due to missingness)
AIC: Inf
```

```
glm(formula = outcome_dichot_new ~ Streptococcus + antibact_score_sum +
    anc + alc + spo2_support_dichot, family = binomial(link = "logit"),
    data = df)

Deviance Residuals:
    Min       1Q   Median       3Q      Max
-1.8108  -0.5735  -0.2477  -0.1318   2.7858

Coefficients:
                     Estimate Std. Error z value Pr(>|z|)
(Intercept)         -4.213398   0.740389  -5.691 1.26e-08 ***
Streptococcus       -0.168466   0.150184  -1.122 0.261977
antibact_score_sum   0.004742   0.001235   3.840 0.000123 ***
anc                  0.040471   0.044807   0.903 0.366413
alc                 -0.249762   0.322972  -0.773 0.439331
spo2_support_dichot  2.162336   0.536887   4.028 5.64e-05 ***
---
Signif. codes:  0 '***' 0.001 '**' 0.01 '*' 0.05 '.' 0.1 ' ' 1

(Dispersion parameter for binomial family taken to be 1)

    Null deviance: 209.51  on 214  degrees of freedom
Residual deviance: 144.71  on 209  degrees of freedom
  (14 observations deleted due to missingness)
AIC: 156.71
```

```
Causal Mediation Analysis

Nonparametric Bootstrap Confidence Intervals with the BCa Method

                          Estimate 95% CI Lower 95% CI Upper p-value
ACME (control)            0.012526    -0.003403         0.06    0.18
ACME (treated)            0.061211     0.006553         0.57    0.18
ADE (control)             0.479864     0.087508         0.72  <2e-16 ***
ADE (treated)             0.528549     0.283070         0.74  <2e-16 ***
Total Effect              0.541075     0.306181         0.76  <2e-16 ***
Prop. Mediated (control)  0.023151    -0.004035         0.14    0.18
Prop. Mediated (treated)  0.113129     0.000145         0.89    0.18
ACME (average)            0.036869     0.002864         0.30    0.18
ADE (average)             0.504206     0.250527         0.73  <2e-16 ***
Prop. Mediated (average)  0.068140    -0.001808         0.51    0.18
---
Signif. codes:  0 '***' 0.001 '**' 0.01 '*' 0.05 '.' 0.1 ' ' 1

Sample Size Used: 215
```

VEILLONELLA

```
glm(formula = Veillonella ~ antibact_score_sum + anc + alc + 
    spo2_support_dichot, family = "poisson", data = df)

Deviance Residuals: 
    Min       1Q   Median       3Q      Max  
-2.8036  -1.3835  -1.1153  -0.7109   3.8065  

Coefficients:
                      Estimate Std. Error z value Pr(>|z|)    
(Intercept)          0.2138515  0.1479231   1.446    0.148    
antibact_score_sum  -0.0022175  0.0004618  -4.802 1.57e-06 ***
anc                  0.0581403  0.0120355   4.831 1.36e-06 ***
alc                 -0.0806702  0.0620630  -1.300    0.194    
spo2_support_dichot -0.1357852  0.1592567  -0.853    0.394    
---
Signif. codes:  0 '***' 0.001 '**' 0.01 '*' 0.05 '.' 0.1 ' ' 1

(Dispersion parameter for poisson family taken to be 1)

    Null deviance: 586.15  on 214  degrees of freedom
Residual deviance: 534.05  on 210  degrees of freedom
  (14 observations deleted due to missingness)
AIC: Inf
```

```
glm(formula = outcome_dichot_new ~ Veillonella + antibact_score_sum + 
    anc + alc + spo2_support_dichot, family = binomial(link = "logit"), 
    data = df)

Deviance Residuals: 
     Min        1Q    Median        3Q       Max  
-1.81605  -0.56422  -0.22332  -0.08469   2.75029  

Coefficients:
                     Estimate Std. Error z value Pr(>|z|)    
(Intercept)         -4.321639   0.727396  -5.941 2.83e-09 ***
Veillonella         -0.448778   0.221544  -2.026   0.0428 *  
antibact_score_sum   0.004801   0.001214   3.955 7.64e-05 ***
anc                  0.078573   0.051210   1.534   0.1250    
alc                 -0.256463   0.316191  -0.811   0.4173    
spo2_support_dichot  2.239154   0.539582   4.150 3.33e-05 ***
---
Signif. codes:  0 '***' 0.001 '**' 0.01 '*' 0.05 '.' 0.1 ' ' 1

(Dispersion parameter for binomial family taken to be 1)

    Null deviance: 209.51  on 214  degrees of freedom
Residual deviance: 140.49  on 209  degrees of freedom
  (14 observations deleted due to missingness)
AIC: 152.49
```

```
Causal Mediation Analysis 

Nonparametric Bootstrap Confidence Intervals with the BCa Method

                         Estimate 95% CI Lower 95% CI Upper p-value    
ACME (control)            0.02437      0.00577         0.09   0.008 ** 
ACME (treated)            0.09227      0.03006         0.37   0.004 ** 
ADE (control)             0.45694      0.17884         0.73  <2e-16 ***
ADE (treated)             0.52484      0.26989         0.78  <2e-16 ***
Total Effect              0.54921      0.31695         0.79  <2e-16 ***
Prop. Mediated (control)  0.04438      0.01246         0.29   0.008 ** 
Prop. Mediated (treated)  0.16800      0.06040         0.83   0.004 ** 
ACME (average)            0.05832      0.01922         0.22   0.004 ** 
ADE (average)             0.49089      0.24401         0.76  <2e-16 ***
Prop. Mediated (average)  0.10619      0.03799         0.48   0.004 ** 
---
Signif. codes:  0 '***' 0.001 '**' 0.01 '*' 0.05 '.' 0.1 ' ' 1

Sample Size Used: 215 
```

Univariate Causal Mediation Analysis for % of Association between Anti-Anaerobic Exposure & In-Hospital Mortality that is Mediated by Anti-Anaerobic Exposure-Induced Changes in BAL Microbes

ACTINOMYCES

```
glm(formula = Actinomyces ~ anaerobe_sum, family = "poisson",
    data = df)

Deviance Residuals:
    Min       1Q   Median       3Q      Max
-1.2964  -1.2964  -1.0810  -0.7985   4.7441

Coefficients:
             Estimate Std. Error z value Pr(>|z|)
(Intercept)  -0.17402    0.09915  -1.755   0.0793 .
anaerobe_sum -0.12113    0.02970  -4.079 4.52e-05 ***
---
Signif. codes:  0 '***' 0.001 '**' 0.01 '*' 0.05 '.' 0.1 ' ' 1

(Dispersion parameter for poisson family taken to be 1)

    Null deviance: 537.16  on 228  degrees of freedom
Residual deviance: 517.09  on 227  degrees of freedom
AIC: Inf
```

```
glm(formula = outcome_dichot ~ Actinomyces + anaerobe_sum, family = binomial(link = "logit"),
    data = df)

Deviance Residuals:
    Min       1Q   Median       3Q      Max
-1.1207  -0.6790  -0.5917  -0.3764   2.3032

Coefficients:
             Estimate Std. Error z value Pr(>|z|)
(Intercept)  -1.65389    0.24978  -6.621 3.56e-11 ***
Actinomyces  -0.24049    0.16032  -1.500   0.1336
anaerobe_sum  0.10127    0.04411   2.296   0.0217 *
---
Signif. codes:  0 '***' 0.001 '**' 0.01 '*' 0.05 '.' 0.1 ' ' 1

(Dispersion parameter for binomial family taken to be 1)

    Null deviance: 226.95  on 228  degrees of freedom
Residual deviance: 217.94  on 226  degrees of freedom
AIC: 223.94
```

```
Causal Mediation Analysis

Nonparametric Bootstrap Confidence Intervals with the BCa Method

                          Estimate 95% CI Lower 95% CI Upper p-value
ACME (control)            2.39e-02     1.23e-03         0.08   0.088 .
ACME (treated)            1.27e-07     3.52e-06         0.54   0.080 .
ADE (control)             8.63e-01    -1.92e-01         0.86   0.044 *
ADE (treated)             8.39e-01    -2.71e-01         0.85   0.044 *
Total Effect              8.63e-01    -1.91e-01         0.86   0.048 *
Prop. Mediated (control)  2.76e-02    -5.00e-01         0.07   0.120
Prop. Mediated (treated)  1.47e-07     1.07e-03         8.13   0.112
ACME (average)            1.19e-02     2.79e-03         0.26   0.088 .
ADE (average)             8.51e-01    -2.30e-01         0.86   0.044 *
Prop. Mediated (average)  1.38e-02     1.83e-02         4.54   0.120
---
Signif. codes:  0 '***' 0.001 '**' 0.01 '*' 0.05 '.' 0.1 ' ' 1

Sample Size Used: 229


Simulations: 500
```

BACTEROIDES

```
glm(formula = Bacteroides ~ anaerobe_sum, family = "poisson",
    data = df)

Deviance Residuals:
    Min       1Q   Median       3Q      Max
-1.1368  -1.1368  -0.6834  -0.2926   4.2456

Coefficients:
             Estimate Std. Error z value Pr(>|z|)
(Intercept)  -0.43674    0.11908  -3.668 0.000245 ***
anaerobe_sum -0.33927    0.06595  -5.144 2.68e-07 ***
---
Signif. codes:  0 '***' 0.001 '**' 0.01 '*' 0.05 '.' 0.1 ' ' 1

(Dispersion parameter for poisson family taken to be 1)

    Null deviance: 365.77  on 228  degrees of freedom
Residual deviance: 315.78  on 227  degrees of freedom
AIC: Inf
```

```
glm(formula = outcome_dichot ~ Bacteroides + anaerobe_sum, family = binomial(link = "logit"),
    data = df)

Deviance Residuals:
    Min       1Q   Median       3Q      Max
-1.0936  -0.6601  -0.5750  -0.4695   2.2404

Coefficients:
             Estimate Std. Error z value Pr(>|z|)
(Intercept)   -1.7161     0.2541  -6.755 1.43e-11 ***
Bacteroides   -0.1519     0.2082  -0.730   0.4655
anaerobe_sum   0.1011     0.0445   2.271   0.0232 *
---
Signif. codes:  0 '***' 0.001 '**' 0.01 '*' 0.05 '.' 0.1 ' ' 1

(Dispersion parameter for binomial family taken to be 1)

    Null deviance: 226.95  on 228  degrees of freedom
Residual deviance: 220.20  on 226  degrees of freedom
AIC: 226.2
```

```
Causal Mediation Analysis

Nonparametric Bootstrap Confidence Intervals with the BCa Method

                         Estimate 95% CI Lower 95% CI Upper p-value
ACME (control)           1.16e-02    -1.03e-02         0.08   0.320
ACME (treated)           5.90e-08     9.95e-05         0.34   0.320
ADE (control)            8.59e-01    -1.83e-01         0.88   0.028 *
ADE (treated)            8.48e-01    -2.13e-01         0.87   0.028 *
Total Effect             8.59e-01    -1.80e-01         0.89   0.028 *
Prop. Mediated (control) 1.35e-02    -1.58e-02         0.09   0.332
Prop. Mediated (treated) 6.87e-08     1.60e-04         0.50   0.332
ACME (average)           5.81e-03    -3.15e-03         0.09   0.320
ADE (average)            8.53e-01    -1.97e-01         0.88   0.028 *
Prop. Mediated (average) 6.76e-03    -5.09e-03         0.12   0.332
---
Signif. codes:  0 '***' 0.001 '**' 0.01 '*' 0.05 '.' 0.1 ' ' 1

Sample Size Used: 229


Simulations:  500
```

CAPNOCYTOPHAGA

```
glm(formula = Capnocytophaga ~ anaerobe_sum, family = "poisson",
    data = df)

Deviance Residuals:
    Min       1Q   Median       3Q      Max
-1.3199  -1.3199  -0.9663  -0.5919   3.9727

Coefficients:
             Estimate Std. Error z value Pr(>|z|)
(Intercept)  -0.13802    0.09900  -1.394    0.163
anaerobe_sum -0.17821    0.03502  -5.089  3.6e-07 ***
---
Signif. codes:  0 '***' 0.001 '**' 0.01 '*' 0.05 '.' 0.1 ' ' 1

(Dispersion parameter for poisson family taken to be 1)

    Null deviance: 482.31  on 228  degrees of freedom
Residual deviance: 447.42  on 227  degrees of freedom
AIC: Inf
```

```
glm(formula = outcome_dichot ~ Capnocytophaga + anaerobe_sum,
    family = binomial(link = "logit"), data = df)

Deviance Residuals:
    Min       1Q   Median       3Q      Max
-1.0986  -0.7006  -0.6197  -0.2641   3.0389

Coefficients:
               Estimate Std. Error z value Pr(>|z|)
(Intercept)    -1.55255    0.24974  -6.217 5.08e-10 ***
Capnocytophaga -0.52148    0.25141  -2.074   0.0381 *
anaerobe_sum    0.09096    0.04443   2.047   0.0406 *
---
Signif. codes:  0 '***' 0.001 '**' 0.01 '*' 0.05 '.' 0.1 ' ' 1

(Dispersion parameter for binomial family taken to be 1)

    Null deviance: 226.95  on 228  degrees of freedom
Residual deviance: 213.72  on 226  degrees of freedom
AIC: 219.72
```

```
Causal Mediation Analysis

Nonparametric Bootstrap Confidence Intervals with the BCa Method

                          Estimate 95% CI Lower 95% CI Upper p-value
ACME (control)            4.51e-02     1.22e-02         0.15   0.024 *
ACME (treated)            1.62e-06     1.29e-07         0.63   0.020 *
ADE (control)             8.70e-01    -2.16e-01         0.88   0.068 .
ADE (treated)             8.25e-01    -2.89e-01         0.82   0.068 .
Total Effect              8.70e-01    -2.14e-01         0.88   0.060 .
Prop. Mediated (control)  5.18e-02    -3.77e-01         0.17   0.076 .
Prop. Mediated (treated)  1.87e-06     6.71e-08         6.17   0.072 .
ACME (average)            2.26e-02     1.38e-02         0.38   0.024 *
ADE (average)             8.48e-01    -2.50e-01         0.85   0.068 .
Prop. Mediated (average)  2.59e-02     1.70e-02         5.55   0.076 .
---
Signif. codes:  0 '***' 0.001 '**' 0.01 '*' 0.05 '.' 0.1 ' ' 1

Sample Size Used: 229


Simulations:  500
```

FUSOBACTERIUM

```
glm(formula = Fusobacterium ~ anaerobe_sum, family = "poisson",
    data = df)

Deviance Residuals:
    Min       1Q   Median       3Q      Max
-1.3103  -1.3103  -1.0112  -0.6566   4.4773

Coefficients:
             Estimate Std. Error z value Pr(>|z|)
(Intercept)  -0.15260    0.09958  -1.532    0.125
anaerobe_sum -0.17275    0.03467  -4.983 6.27e-07 ***
---
Signif. codes:  0 '***' 0.001 '**' 0.01 '*' 0.05 '.' 0.1 ' ' 1

(Dispersion parameter for poisson family taken to be 1)

    Null deviance: 486.88  on 228  degrees of freedom
Residual deviance: 453.80  on 227  degrees of freedom
AIC: Inf
```

```
glm(formula = outcome_dichot ~ Fusobacterium + anaerobe_sum,
    family = binomial(link = "logit"), data = df)

Deviance Residuals:
    Min       1Q   Median       3Q      Max
-1.0937  -0.6958  -0.6151  -0.2782   2.7054

Coefficients:
              Estimate Std. Error z value Pr(>|z|)
(Intercept)   -1.56885    0.24947  -6.289  3.2e-10 ***
Fusobacterium -0.45287    0.23214  -1.951   0.0511 .
anaerobe_sum   0.09124    0.04413   2.067   0.0387 *
---
Signif. codes:  0 '***' 0.001 '**' 0.01 '*' 0.05 '.' 0.1 ' ' 1

(Dispersion parameter for binomial family taken to be 1)

    Null deviance: 226.95  on 228  degrees of freedom
Residual deviance: 214.88  on 226  degrees of freedom
AIC: 220.88
```

```
Causal Mediation Analysis

Nonparametric Bootstrap Confidence Intervals with the BCa Method

                          Estimate 95% CI Lower 95% CI Upper p-value
ACME (control)            4.08e-02     1.91e-02         0.11   0.004 **
ACME (treated)            1.49e-06     4.00e-06         0.55   0.004 **
ADE (control)             8.68e-01    -1.57e-01         0.88   0.032 *
ADE (treated)             8.28e-01    -2.35e-01         0.86   0.032 *
Total Effect              8.68e-01    -1.49e-01         0.90   0.028 *
Prop. Mediated (control)  4.70e-02     5.38e-03         0.12   0.032 *
Prop. Mediated (treated)  1.71e-06     5.67e-06         3.12   0.032 *
ACME (average)            2.04e-02     1.57e-02         0.32   0.004 **
ADE (average)             8.48e-01    -2.00e-01         0.87   0.032 *
Prop. Mediated (average)  2.35e-02     1.92e-02         0.42   0.032 *
---
Signif. codes:  0 '***' 0.001 '**' 0.01 '*' 0.05 '.' 0.1 ' ' 1

Sample Size Used: 229


Simulations:  500
```

GEMELLA

```
glm(formula = Gemella ~ anaerobe_sum, family = "poisson", data = df)

Deviance Residuals:
    Min       1Q   Median       3Q      Max
-1.2726  -1.2726  -0.9267  -0.5462   4.4804

Coefficients:
             Estimate Std. Error z value Pr(>|z|)
(Intercept)  -0.21109    0.10356  -2.038   0.0415 *
anaerobe_sum -0.21146    0.04032  -5.245 1.56e-07 ***
---
Signif. codes:  0 '***' 0.001 '**' 0.01 '*' 0.05 '.' 0.1 ' ' 1

(Dispersion parameter for poisson family taken to be 1)

    Null deviance: 494.61  on 228  degrees of freedom
Residual deviance: 454.94  on 227  degrees of freedom
AIC: Inf
```

```
glm(formula = outcome_dichot ~ Gemella + anaerobe_sum, family = binomial(link = "logit"),
    data = df)

Deviance Residuals:
    Min       1Q   Median       3Q      Max
-1.0867  -0.6916  -0.6117  -0.1909   2.2625

Coefficients:
             Estimate Std. Error z value Pr(>|z|)
(Intercept)  -1.58134    0.24588  -6.431 1.27e-10 ***
Gemella      -0.59762    0.32442  -1.842   0.0655 .
anaerobe_sum  0.09094    0.04409   2.063   0.0392 *
---
Signif. codes:  0 '***' 0.001 '**' 0.01 '*' 0.05 '.' 0.1 ' ' 1

(Dispersion parameter for binomial family taken to be 1)

    Null deviance: 226.95  on 228  degrees of freedom
Residual deviance: 213.52  on 226  degrees of freedom
AIC: 219.52
```

```
Causal Mediation Analysis

Nonparametric Bootstrap Confidence Intervals with the BCa Method

                          Estimate 95% CI Lower 95% CI Upper p-value
ACME (control)            4.91e-02     2.12e-02         0.15  <2e-16 ***
ACME (treated)            2.06e-06     4.99e-08         0.62  <2e-16 ***
ADE (control)             8.78e-01    -1.82e-01         0.89   0.064 .
ADE (treated)             8.29e-01    -2.64e-01         0.82   0.064 .
Total Effect              8.79e-01    -1.84e-01         0.89   0.060 .
Prop. Mediated (control)  5.59e-02    -6.72e-01         0.13   0.060 .
Prop. Mediated (treated)  2.35e-06     2.75e-08         1.82   0.060 .
ACME (average)            2.46e-02     1.58e-02         0.37  <2e-16 ***
ADE (average)             8.54e-01    -2.21e-01         0.86   0.064 .
Prop. Mediated (average)  2.80e-02     1.68e-02         2.35   0.060 .
---
Signif. codes:  0 '***' 0.001 '**' 0.01 '*' 0.05 '.' 0.1 ' ' 1

Sample Size Used: 229


Simulations:  500
```

GRANULICATELLA

```
glm(formula = Granulicatella ~ anaerobe_sum, family = "poisson",
    data = df)

Deviance Residuals:
    Min       1Q   Median       3Q      Max
-1.2439  -1.2439  -0.8376  -0.4333   3.9143

Coefficients:
             Estimate Std. Error z value Pr(>|z|)
(Intercept)  -0.25669    0.10722  -2.394   0.0167 *
anaerobe_sum -0.26364    0.04838  -5.449 5.05e-08 ***
---
Signif. codes:  0 ‘***’ 0.001 ‘**’ 0.01 ‘*’ 0.05 ‘.’ 0.1 ‘ ’ 1

(Dispersion parameter for poisson family taken to be 1)

    Null deviance: 431.23  on 228  degrees of freedom
Residual deviance: 383.42  on 227  degrees of freedom
AIC: Inf
```

```
glm(formula = outcome_dichot ~ Granulicatella + anaerobe_sum,
    family = binomial(link = "logit"), data = df)

Deviance Residuals:
    Min       1Q   Median       3Q      Max
-1.0952  -0.6615  -0.5763  -0.4814   2.2060

Coefficients:
               Estimate Std. Error z value Pr(>|z|)
(Intercept)    -1.71129    0.25635  -6.676 2.46e-11 ***
Granulicatella -0.12706    0.17387  -0.731   0.4649
anaerobe_sum    0.10099    0.04449   2.270   0.0232 *
---
Signif. codes:  0 ‘***’ 0.001 ‘**’ 0.01 ‘*’ 0.05 ‘.’ 0.1 ‘ ’ 1

(Dispersion parameter for binomial family taken to be 1)

    Null deviance: 226.95  on 228  degrees of freedom
Residual deviance: 220.20  on 226  degrees of freedom
AIC: 226.2
```

```
Causal Mediation Analysis

Nonparametric Bootstrap Confidence Intervals with the BCa Method

                          Estimate 95% CI Lower 95% CI Upper p-value
ACME (control)            1.24e-02    -1.23e-02         0.07   0.440
ACME (treated)            6.08e-08     1.12e-04         0.28   0.432
ADE (control)             8.59e-01    -2.62e-01         0.86   0.044 *
ADE (treated)             8.47e-01    -3.23e-01         0.86   0.044 *
Total Effect              8.59e-01    -2.60e-01         0.86   0.044 *
Prop. Mediated (control)  1.44e-02    -2.27e-02         0.07   0.484
Prop. Mediated (treated)  7.07e-08     1.31e-03         0.79   0.476
ACME (average)            6.18e-03    -5.16e-03         0.05   0.440
ADE (average)             8.53e-01    -2.93e-01         0.86   0.044 *
Prop. Mediated (average)  7.20e-03    -6.65e-03         0.30   0.484
---
Signif. codes:  0 ‘***’ 0.001 ‘**’ 0.01 ‘*’ 0.05 ‘.’ 0.1 ‘ ’ 1

Sample Size Used: 229


Simulations:  500
```

HAEMOPHILUS

```
glm(formula = Haemophilus ~ antibact_score_sum, family = "poisson",
    data = df)

Deviance Residuals:
    Min       1Q   Median       3Q      Max
 -7.540   -4.861   -2.370   -1.321  101.805

Coefficients:
                     Estimate Std. Error z value Pr(>|z|)
(Intercept)         3.3473128  0.0293948  113.87   <2e-16 ***
antibact_score_sum -0.0092048  0.0002605  -35.34   <2e-16 ***
---
Signif. codes:  0 '***' 0.001 '**' 0.01 '*' 0.05 '.' 0.1 ' ' 1

(Dispersion parameter for poisson family taken to be 1)

    Null deviance: 17523  on 228  degrees of freedom
Residual deviance: 15263  on 227  degrees of freedom
AIC: Inf
```

```
glm(formula = outcome_dichot ~ Haemophilus + anaerobe_sum, family = binomial(link = "logit"),
    data = df)

Deviance Residuals:
    Min       1Q   Median       3Q      Max
-1.1054  -0.7054  -0.6241  -0.2671   2.3114

Coefficients:
             Estimate Std. Error z value Pr(>|z|)
(Intercept)  -1.53711    0.25049  -6.136 8.44e-10 ***
Haemophilus  -0.35609    0.16802  -2.119   0.0341 *
anaerobe_sum  0.09103    0.04410   2.064   0.0390 *
---
Signif. codes:  0 '***' 0.001 '**' 0.01 '*' 0.05 '.' 0.1 ' ' 1

(Dispersion parameter for binomial family taken to be 1)

    Null deviance: 226.95  on 228  degrees of freedom
Residual deviance: 214.01  on 226  degrees of freedom
AIC: 220.01
```

```
Causal Mediation Analysis

Nonparametric Bootstrap Confidence Intervals with the BCa Method

                          Estimate 95% CI Lower 95% CI Upper p-value
ACME (control)            4.95e-02     2.26e-02         0.13  <2e-16 ***
ACME (treated)            1.53e-06     1.39e-04         0.60  <2e-16 ***
ADE (control)             8.73e-01    -2.06e-01         0.87   0.068 .
ADE (treated)             8.23e-01    -2.81e-01         0.83   0.068 .
Total Effect              8.73e-01    -2.04e-01         0.87   0.060 .
Prop. Mediated (control)  5.68e-02    -7.48e+00         0.07   0.060 .
Prop. Mediated (treated)  1.75e-06     1.65e-04        10.45   0.060 .
ACME (average)            2.48e-02     1.95e-02         0.37  <2e-16 ***
ADE (average)             8.48e-01    -2.44e-01         0.85   0.068 .
Prop. Mediated (average)  2.84e-02     9.50e-03         0.36   0.060 .
---
Signif. codes:  0 '***' 0.001 '**' 0.01 '*' 0.05 '.' 0.1 ' ' 1

Sample Size Used: 229


Simulations: 500
```

NEISSERIA

```
glm(formula = Neisseria ~ anaerobe_sum, family = "poisson", data = df)

Deviance Residuals:
    Min       1Q   Median       3Q      Max
-1.2613  -1.2613  -1.1129  -0.9033   4.5048

Coefficients:
             Estimate Std. Error z value Pr(>|z|)
(Intercept)  -0.22882    0.10063  -2.274  0.02298 *
anaerobe_sum -0.08347    0.02700  -3.092  0.00199 **
---
Signif. codes:  0 '***' 0.001 '**' 0.01 '*' 0.05 '.' 0.1 ' ' 1

(Dispersion parameter for poisson family taken to be 1)

    Null deviance: 598.53  on 228  degrees of freedom
Residual deviance: 587.74  on 227  degrees of freedom
AIC: Inf
```

```
glm(formula = outcome_dichot ~ Neisseria + anaerobe_sum, family = binomial(link = "logit"),
    data = df)

Deviance Residuals:
    Min       1Q   Median       3Q      Max
-1.1248  -0.6688  -0.5797  -0.4149   2.0348

Coefficients:
             Estimate Std. Error z value Pr(>|z|)
(Intercept)  -1.69856    0.24685  -6.881 5.95e-12 ***
Neisseria    -0.15342    0.13133  -1.168   0.2427
anaerobe_sum  0.10490    0.04364   2.403   0.0162 *
---
Signif. codes:  0 '***' 0.001 '**' 0.01 '*' 0.05 '.' 0.1 ' ' 1

(Dispersion parameter for binomial family taken to be 1)

    Null deviance: 226.95  on 228  degrees of freedom
Residual deviance: 219.17  on 226  degrees of freedom
AIC: 225.17
```

```
Causal Mediation Analysis

Nonparametric Bootstrap Confidence Intervals with the BCa Method

                          Estimate 95% CI Lower 95% CI Upper p-value
ACME (control)            1.56e-02    -8.61e-04         0.13   0.172
ACME (treated)            4.25e-08     3.12e-06         0.65   0.172
ADE (control)             8.61e-01    -1.51e-01         0.87   0.016 *
ADE (treated)             8.45e-01    -2.06e-01         0.86   0.016 *
Total Effect              8.61e-01    -1.48e-01         0.87   0.028 *
Prop. Mediated (control)  1.82e-02     5.36e-03         1.00   0.176
Prop. Mediated (treated)  4.93e-08     1.24e-02         8.51   0.176
ACME (average)            7.82e-03     1.36e-03         0.43   0.172
ADE (average)             8.53e-01    -1.74e-01         0.87   0.016 *
Prop. Mediated (average)  9.08e-03     2.01e-02         4.75   0.176
---
Signif. codes:  0 '***' 0.001 '**' 0.01 '*' 0.05 '.' 0.1 ' ' 1

Sample Size Used: 229
```

PREVOTELLA

```
glm(formula = Prevotella ~ anaerobe_sum, family = "poisson",
    data = df)

Deviance Residuals:
    Min       1Q   Median       3Q      Max
-1.8275  -1.6637  -1.0402   0.8553   5.0445

Coefficients:
             Estimate Std. Error z value Pr(>|z|)
(Intercept)   0.51278    0.07168   7.153 8.47e-13 ***
anaerobe_sum -0.18783    0.02607  -7.205 5.82e-13 ***
---
Signif. codes:  0 '***' 0.001 '**' 0.01 '*' 0.05 '.' 0.1 ' ' 1

(Dispersion parameter for poisson family taken to be 1)

    Null deviance: 681.80  on 228  degrees of freedom
Residual deviance: 610.51  on 227  degrees of freedom
AIC: Inf
```

```
glm(formula = outcome_dichot ~ Prevotella + anaerobe_sum, family = binomial(link = "logit"),
    data = df)

Deviance Residuals:
    Min       1Q   Median       3Q      Max
-1.0695  -0.7062  -0.6592  -0.2805   2.7243

Coefficients:
             Estimate Std. Error z value Pr(>|z|)
(Intercept)  -1.41590    0.26182  -5.408 6.38e-08 ***
Prevotella   -0.36737    0.14728  -2.494   0.0126 *
anaerobe_sum  0.07711    0.04528   1.703   0.0886 .
---
Signif. codes:  0 '***' 0.001 '**' 0.01 '*' 0.05 '.' 0.1 ' ' 1

(Dispersion parameter for binomial family taken to be 1)

    Null deviance: 226.95  on 228  degrees of freedom
Residual deviance: 211.93  on 226  degrees of freedom
AIC: 217.93
```

```
Causal Mediation Analysis

Nonparametric Bootstrap Confidence Intervals with the BCa Method

                          Estimate 95% CI Lower 95% CI Upper p-value
ACME (control)            7.17e-02     3.10e-02         0.14  <2e-16 ***
ACME (treated)            2.20e-05     1.90e-05         0.61  <2e-16 ***
ADE (control)             8.76e-01    -2.38e-01         0.88    0.08 .
ADE (treated)             8.05e-01    -3.36e-01         0.81    0.08 .
Total Effect              8.76e-01    -2.40e-01         0.87    0.06 .
Prop. Mediated (control)  8.18e-02     1.02e-01        33.55    0.06 .
Prop. Mediated (treated)  2.51e-05     3.81e-04        23.28    0.06 .
ACME (average)            3.59e-02     2.36e-02         0.35  <2e-16 ***
ADE (average)             8.41e-01    -2.88e-01         0.84    0.08 .
Prop. Mediated (average)  4.09e-02     5.47e-02        29.26    0.06 .
---
Signif. codes:  0 '***' 0.001 '**' 0.01 '*' 0.05 '.' 0.1 ' ' 1

Sample Size Used: 229


Simulations: 500
```

PORPHYROMONAS

```
glm(formula = Porphyromonas ~ anaerobe_sum, family = "poisson",
    data = df)

Deviance Residuals:
    Min       1Q   Median       3Q      Max
-0.6957  -0.6957  -0.6593  -0.5610   4.6813

Coefficients:
             Estimate Std. Error z value Pr(>|z|)
(Intercept)  -1.41874    0.18043  -7.863 3.75e-15 ***
anaerobe_sum -0.05383    0.04442  -1.212    0.226
---
Signif. codes:  0 '***' 0.001 '**' 0.01 '*' 0.05 '.' 0.1 ' ' 1

(Dispersion parameter for poisson family taken to be 1)

    Null deviance: 246.85  on 228  degrees of freedom
Residual deviance: 245.27  on 227  degrees of freedom
AIC: Inf
```

```
glm(formula = outcome_dichot ~ Porphyromonas + anaerobe_sum,
    family = binomial(link = "logit"), data = df)

Deviance Residuals:
    Min       1Q   Median       3Q      Max
-1.1336  -0.6686  -0.5780  -0.3214   2.4991

Coefficients:
              Estimate Std. Error z value Pr(>|z|)
(Intercept)   -1.70472    0.24139  -7.062 1.64e-12 ***
Porphyromonas -0.63617    0.45207  -1.407   0.1594
anaerobe_sum   0.10672    0.04366   2.444   0.0145 *
---
Signif. codes:  0 '***' 0.001 '**' 0.01 '*' 0.05 '.' 0.1 ' ' 1

(Dispersion parameter for binomial family taken to be 1)

    Null deviance: 226.95  on 228  degrees of freedom
Residual deviance: 217.46  on 226  degrees of freedom
AIC: 223.46
```

```
Causal Mediation Analysis

Nonparametric Bootstrap Confidence Intervals with the BCa Method

                         Estimate 95% CI Lower 95% CI Upper p-value
ACME (control)           9.86e-03    -2.00e-01         0.01   0.412
ACME (treated)           3.02e-08    -1.00e+00         0.00   0.408
ADE (control)            8.56e-01     7.80e-02         0.90   0.008 **
ADE (treated)            8.46e-01    -2.01e-01         0.84   0.008 **
Total Effect             8.56e-01    -2.02e-01         0.85   0.172
Prop. Mediated (control) 1.15e-02     3.51e-02         2.77   0.256
Prop. Mediated (treated) 3.53e-08     1.13e-02        16.30   0.252
ACME (average)           4.93e-03    -5.83e-01         0.00   0.416
ADE (average)            8.51e-01    -1.93e-01         0.86   0.008 **
Prop. Mediated (average) 5.76e-03     2.36e-02         9.54   0.260
---
Signif. codes:  0 '***' 0.001 '**' 0.01 '*' 0.05 '.' 0.1 ' ' 1

Sample Size Used: 229


Simulations:  500
```

ROTHIA

```
glm(formula = Rothia ~ anaerobe_sum, family = "poisson", data = df)

Deviance Residuals:
   Min      1Q  Median      3Q     Max
-1.484  -1.484  -1.181  -0.887   4.473

Coefficients:
             Estimate Std. Error z value Pr(>|z|)
(Intercept)   0.09682    0.08641   1.120    0.263
anaerobe_sum -0.11443    0.02538  -4.509 6.51e-06 ***
---
Signif. codes:  0 '***' 0.001 '**' 0.01 '*' 0.05 '.' 0.1 ' ' 1

(Dispersion parameter for poisson family taken to be 1)

    Null deviance: 642.73  on 228  degrees of freedom
Residual deviance: 618.51  on 227  degrees of freedom
AIC: Inf
```

```
glm(formula = outcome_dichot ~ Rothia + anaerobe_sum, family = binomial(link = "logit"),
    data = df)

Deviance Residuals:
    Min       1Q   Median       3Q      Max
-1.1127  -0.7120  -0.6303  -0.2082   2.3787

Coefficients:
             Estimate Std. Error z value Pr(>|z|)
(Intercept)  -1.51512    0.24768  -6.117 9.52e-10 ***
Rothia       -0.48088    0.20830  -2.309   0.0210 *
anaerobe_sum  0.09073    0.04409   2.058   0.0396 *
---
Signif. codes:  0 '***' 0.001 '**' 0.01 '*' 0.05 '.' 0.1 ' ' 1

(Dispersion parameter for binomial family taken to be 1)

    Null deviance: 226.95  on 228  degrees of freedom
Residual deviance: 210.80  on 226  degrees of freedom
AIC: 216.8
```

```
Causal Mediation Analysis

Nonparametric Bootstrap Confidence Intervals with the BCa Method

                          Estimate 95% CI Lower 95% CI Upper p-value
ACME (control)            6.04e-02     2.96e-02         0.14  <2e-16 ***
ACME (treated)            2.30e-06     1.34e-06         0.59  <2e-16 ***
ADE (control)             8.80e-01    -1.74e-01         0.88   0.036 *
ADE (treated)             8.20e-01    -2.44e-01         0.83   0.036 *
Total Effect              8.80e-01    -1.72e-01         0.89   0.036 *
Prop. Mediated (control)  6.86e-02    -5.43e-01         0.14   0.036 *
Prop. Mediated (treated)  2.61e-06     3.38e-06         0.84   0.036 *
ACME (average)            3.02e-02     2.22e-02         0.36  <2e-16 ***
ADE (average)             8.50e-01    -2.03e-01         0.86   0.036 *
Prop. Mediated (average)  3.43e-02     2.30e-02         0.74   0.036 *
---
Signif. codes:  0 '***' 0.001 '**' 0.01 '*' 0.05 '.' 0.1 ' ' 1

Sample Size Used: 229


Simulations: 500
```

SCHAALIA

```
glm(formula = Schaalia ~ anaerobe_sum, family = "poisson", data = df)

Deviance Residuals:
    Min       1Q   Median       3Q      Max
-1.4842  -1.4842  -1.0390  -0.6653   4.9206

Coefficients:
             Estimate Std. Error z value Pr(>|z|)
(Intercept)   0.09654    0.08805   1.096    0.273
anaerobe_sum -0.17830    0.03115  -5.724 1.04e-08 ***
---
Signif. codes:  0 '***' 0.001 '**' 0.01 '*' 0.05 '.' 0.1 ' ' 1

(Dispersion parameter for poisson family taken to be 1)

    Null deviance: 591.53  on 228  degrees of freedom
Residual deviance: 547.39  on 227  degrees of freedom
AIC: Inf
```

```
glm(formula = outcome_dichot ~ Schaalia + anaerobe_sum, family = binomial(link = "logit"),
    data = df)

Deviance Residuals:
    Min       1Q   Median       3Q      Max
-1.0849  -0.7030  -0.6249  -0.2656   2.7343

Coefficients:
             Estimate Std. Error z value Pr(>|z|)
(Intercept)  -1.53435    0.25099  -6.113 9.77e-10 ***
Schaalia     -0.41768    0.19852  -2.104   0.0354 *
anaerobe_sum  0.08752    0.04438   1.972   0.0486 *
---
Signif. codes:  0 '***' 0.001 '**' 0.01 '*' 0.05 '.' 0.1 ' ' 1

(Dispersion parameter for binomial family taken to be 1)

    Null deviance: 226.95  on 228  degrees of freedom
Residual deviance: 213.63  on 226  degrees of freedom
AIC: 219.63
```

```
Causal Mediation Analysis

Nonparametric Bootstrap Confidence Intervals with the BCa Method

                          Estimate 95% CI Lower 95% CI Upper p-value
ACME (control)            5.39e-02     2.39e-02         0.16   0.008 **
ACME (treated)            3.17e-06     3.18e-06         0.73   0.004 **
ADE (control)             8.77e-01    -1.76e-01         0.87   0.064 .
ADE (treated)             8.23e-01    -2.68e-01         0.82   0.064 .
Total Effect              8.77e-01    -1.76e-01         0.88   0.060 .
Prop. Mediated (control)  6.15e-02     2.76e-02         0.25   0.068 .
Prop. Mediated (treated)  3.62e-06     5.13e-06         7.90   0.064 .
ACME (average)            2.70e-02     2.16e-02         0.45   0.008 **
ADE (average)             8.50e-01    -2.13e-01         0.85   0.064 .
Prop. Mediated (average)  3.08e-02     2.54e-02         2.99   0.068 .
---
Signif. codes:  0 '***' 0.001 '**' 0.01 '*' 0.05 '.' 0.1 ' ' 1

Sample Size Used: 229


Simulations: 500
```

STAPHYLOCOCCUS

```
glm(formula = Staphylococcus ~ antibact_score_sum, family = "poisson",
    data = df)

Deviance Residuals:
   Min      1Q  Median      3Q     Max
-1.167  -1.143  -1.100  -1.059  22.777

Coefficients:
                     Estimate Std. Error z value Pr(>|z|)
(Intercept)        -0.3845185  0.1341187  -2.867  0.00414 **
antibact_score_sum -0.0004437  0.0004558  -0.974  0.33029
---
Signif. codes:  0 '***' 0.001 '**' 0.01 '*' 0.05 '.' 0.1 ' ' 1

(Dispersion parameter for poisson family taken to be 1)

    Null deviance: 1108.1  on 228  degrees of freedom
Residual deviance: 1107.1  on 227  degrees of freedom
AIC: Inf
```

```
glm(formula = outcome_dichot ~ Staphylococcus + anaerobe_sum,
    family = binomial(link = "logit"), data = df)

Deviance Residuals:
    Min       1Q   Median       3Q      Max
-1.0982  -0.6913  -0.5380  -0.5380   2.0022

Coefficients:
               Estimate Std. Error z value Pr(>|z|)
(Intercept)    -1.85957    0.25396  -7.322 2.44e-13 ***
Staphylococcus  0.09746    0.10157   0.960   0.3373
anaerobe_sum    0.10999    0.04359   2.523   0.0116 *
---
Signif. codes:  0 '***' 0.001 '**' 0.01 '*' 0.05 '.' 0.1 ' ' 1

(Dispersion parameter for binomial family taken to be 1)

    Null deviance: 226.95  on 228  degrees of freedom
Residual deviance: 219.91  on 226  degrees of freedom
AIC: 225.91
```

```
Causal Mediation Analysis

Nonparametric Bootstrap Confidence Intervals with the BCa Method

                          Estimate 95% CI Lower 95% CI Upper p-value
ACME (control)           -5.03e-03    -6.36e-03         0.93   0.872
ACME (treated)           -5.41e-09    -1.00e+00         0.00   0.860
ADE (control)             8.58e-01    -2.10e-01         0.88   0.024 *
ADE (treated)             8.63e-01    -2.89e-01         0.87   0.024 *
Total Effect              8.58e-01    -2.20e-01         0.86   0.052 .
Prop. Mediated (control) -5.87e-03    -6.87e-03         1.00   0.900
Prop. Mediated (treated) -6.30e-09     3.65e-01        11.09   0.888
ACME (average)           -2.52e-03    -3.20e-03         0.79   0.872
ADE (average)             8.60e-01    -2.50e-01         0.87   0.024 *
Prop. Mediated (average) -2.94e-03    -3.50e-03         6.02   0.900
---
Signif. codes:  0 '***' 0.001 '**' 0.01 '*' 0.05 '.' 0.1 ' ' 1

Sample Size Used: 229


Simulations: 500
```

STREPTOCOCCUS

```
glm(formula = Streptococcus ~ anaerobe_sum, family = "poisson",
    data = df)

Deviance Residuals:
    Min       1Q   Median       3Q      Max
-1.7980  -1.7980  -1.2691  -0.7526   4.8964

Coefficients:
             Estimate Std. Error z value Pr(>|z|)
(Intercept)   0.48025    0.07260   6.615 3.71e-11 ***
anaerobe_sum -0.17418    0.02538  -6.863 6.74e-12 ***
---
Signif. codes:  0 '***' 0.001 '**' 0.01 '*' 0.05 '.' 0.1 ' ' 1

(Dispersion parameter for poisson family taken to be 1)

    Null deviance: 779.89  on 228  degrees of freedom
Residual deviance: 716.96  on 227  degrees of freedom
AIC: Inf
```

```
glm(formula = outcome_dichot ~ Streptococcus + anaerobe_sum,
    family = binomial(link = "logit"), data = df)

Deviance Residuals:
    Min       1Q   Median       3Q      Max
-1.0704  -0.7151  -0.6413  -0.2759   2.4520

Coefficients:
              Estimate Std. Error z value Pr(>|z|)
(Intercept)   -1.47713    0.25384  -5.819 5.91e-09 ***
Streptococcus -0.32164    0.13904  -2.313   0.0207 *
anaerobe_sum   0.08134    0.04431   1.836   0.0664 .
---
Signif. codes:  0 '***' 0.001 '**' 0.01 '*' 0.05 '.' 0.1 ' ' 1

(Dispersion parameter for binomial family taken to be 1)

    Null deviance: 226.95  on 228  degrees of freedom
Residual deviance: 212.69  on 226  degrees of freedom
AIC: 218.69
```

```
Causal Mediation Analysis

Nonparametric Bootstrap Confidence Intervals with the BCa Method

                          Estimate 95% CI Lower 95% CI Upper p-value
ACME (control)            6.06e-02     2.96e-02         0.16  <2e-16 ***
ACME (treated)            8.53e-06     6.43e-05         0.73  <2e-16 ***
ADE (control)             8.75e-01    -2.12e-01         0.87   0.056 .
ADE (treated)             8.14e-01    -2.85e-01         0.82   0.056 .
Total Effect              8.75e-01    -2.12e-01         0.87   0.048 *
Prop. Mediated (control)  6.92e-02     3.10e-02         0.35   0.048 *
Prop. Mediated (treated)  9.75e-06     1.03e-04         5.31   0.048 *
ACME (average)            3.03e-02     2.62e-02         0.44  <2e-16 ***
ADE (average)             8.44e-01    -2.47e-01         0.85   0.056 .
Prop. Mediated (average)  3.46e-02     3.67e-02         5.41   0.048 *
---
Signif. codes:  0 '***' 0.001 '**' 0.01 '*' 0.05 '.' 0.1 ' ' 1

Sample Size Used: 229


Simulations: 500
```

VEILLONELLA

```
glm(formula = Veillonella ~ anaerobe_sum, family = "poisson",
    data = df)

Deviance Residuals:
    Min       1Q   Median       3Q      Max
-1.6965  -1.5480  -1.0730  -0.5156   4.9087

Coefficients:
             Estimate Std. Error z value Pr(>|z|)
(Intercept)   0.36394    0.07713   4.719 2.38e-06 ***
anaerobe_sum -0.18322    0.02768  -6.620 3.59e-11 ***
---
Signif. codes:  0 '***' 0.001 '**' 0.01 '*' 0.05 '.' 0.1 ' ' 1

(Dispersion parameter for poisson family taken to be 1)

    Null deviance: 651.53  on 228  degrees of freedom
Residual deviance: 591.91  on 227  degrees of freedom
AIC: Inf
```

```
glm(formula = outcome_dichot ~ Veillonella + anaerobe_sum, family = binomial(link = "logit"),
    data = df)

Deviance Residuals:
    Min       1Q   Median       3Q      Max
-1.0822  -0.7169  -0.6412  -0.2788   2.5157

Coefficients:
             Estimate Std. Error z value Pr(>|z|)
(Intercept)  -1.47740    0.25810  -5.724 1.04e-08 ***
Veillonella  -0.36506    0.15813  -2.309   0.0210 *
anaerobe_sum  0.08328    0.04507   1.848   0.0646 .
---
Signif. codes:  0 '***' 0.001 '**' 0.01 '*' 0.05 '.' 0.1 ' ' 1

(Dispersion parameter for binomial family taken to be 1)

    Null deviance: 226.95  on 228  degrees of freedom
Residual deviance: 213.05  on 226  degrees of freedom
AIC: 219.05
```

```
Causal Mediation Analysis

Nonparametric Bootstrap Confidence Intervals with the BCa Method

                          Estimate 95% CI Lower 95% CI Upper p-value
ACME (control)            6.30e-02    2.61e-02         0.15  <2e-16 ***
ACME (treated)            6.65e-06    1.10e-05         0.76  <2e-16 ***
ADE (control)             8.77e-01   -2.31e-01         0.88    0.15
ADE (treated)             8.14e-01   -3.15e-01         0.81    0.15
Total Effect              8.77e-01   -2.31e-01         0.87    0.13
Prop. Mediated (control)  7.19e-02   -1.28e+03      -471.61    0.13
Prop. Mediated (treated)  7.58e-06   -6.69e+02      -245.52    0.13
ACME (average)            3.15e-02    2.28e-02         0.42  <2e-16 ***
ADE (average)             8.46e-01   -2.73e-01         0.85    0.15
Prop. Mediated (average)  3.59e-02   -9.76e+02      -358.57    0.13
---
Signif. codes:  0 '***' 0.001 '**' 0.01 '*' 0.05 '.' 0.1 ' ' 1

Sample Size Used: 229


Simulations: 500
```
