# Supplementary material for: Pathobiological signatures of dysbiotic lung injury in pediatric patients undergoing stem cell transplantation
Source: Nat Med. 2024 May 23;30(7):1982–93. doi: 10.1038/s41591-024-02999-4 (PMC11271406; doi:10.1038/s41591-024-02999-4)
Supplement: Supplementary file 1 [file 41591_2024_2999_MOESM2_ESM.pdf]

nature portfolio

Corresponding author(s): Zinter, Matt S.

Last updated by author(s): April 8, 2024

Reporting Summary

Nature Portfolio wishes to improve the reproducibility of the work that we publish. This form provides structure for consistency and transparency in reporting. For further information on Nature Portfolio policies, see our Editorial Policies and the Editorial Policy Checklist.

Statistics

For all statistical analyses, confirm that the following items are present in the figure legend, table legend, main text, or Methods section.

n/a	Confirmed	
☐	☒	The exact sample size (*n*) for each experimental group/condition, given as a discrete number and unit of measurement
☐	☒	A statement on whether measurements were taken from distinct samples or whether the same sample was measured repeatedly
☐	☒	The statistical test(s) used AND whether they are one- or two-sided<br>*Only common tests should be described solely by name; describe more complex techniques in the Methods section.*
☐	☒	A description of all covariates tested
☐	☒	A description of any assumptions or corrections, such as tests of normality and adjustment for multiple comparisons
☐	☒	A full description of the statistical parameters including central tendency (e.g. means) or other basic estimates (e.g. regression coefficient) AND variation (e.g. standard deviation) or associated estimates of uncertainty (e.g. confidence intervals)
☐	☒	For null hypothesis testing, the test statistic (e.g. *F*, *t*, *r*) with confidence intervals, effect sizes, degrees of freedom and *P* value noted<br>*Give P values as exact values whenever suitable.*
☒	☐	For Bayesian analysis, information on the choice of priors and Markov chain Monte Carlo settings
☒	☐	For hierarchical and complex designs, identification of the appropriate level for tests and full reporting of outcomes
☒	☐	Estimates of effect sizes (e.g. Cohen's *d*, Pearson's *r*), indicating how they were calculated

Our web collection on statistics for biologists contains articles on many of the points above.

Software and code

Policy information about availability of computer code

Data collection	Clinical metadata were abstracted from hospital records by trained research assistants under IRB approval and input into a secure REDCap platform housed on UCSF servers. No software were used for this purpose.
Data analysis	Sequencing files were analyzed using the open-source CZID pipeline v7.1 (https://github.com/chanzuckerberg/czid-web) to generate taxonomic counts and human transcript counts and using a custom pipeline of FMAP v0.15, diamond v0.9.24, and the UniRef90 database to generate KEGG alignment counts. Resultant data were all processed in the R statistical platform using publicly available packages including edgeR v3.38.4, DESeq2 v1.38.3, vegan v2.6-4, umap v0.2.10.0, survival v3.5-7 ggplot2 v3.4.4, pheatmap v1.0.12, mediation v4.5.0, and randomforestSRC v3.2.3. CIBERSORTx was run locally through a Docker installation on UCSF Linux servers. Imrep was run locally on UCSF Linux servers. Code is available on GitHub: https://github.com/zinterm/pedBMT_BALseq

For manuscripts utilizing custom algorithms or software that are central to the research but not yet described in published literature, software must be made available to editors and reviewers. We strongly encourage code deposition in a community repository (e.g. GitHub). See the Nature Portfolio guidelines for submitting code & software for further information.

Data

Policy information about availability of data

All manuscripts must include a data availability statement. This statement should provide the following information, where applicable:

- Accession codes, unique identifiers, or web links for publicly available datasets
- A description of any restrictions on data availability
- For clinical datasets or third party data, please ensure that the statement adheres to our policy

Raw sequencing files and instructions to request download are available under controlled access on NIH dbGaP: https://www.ncbi.nlm.nih.gov/projects/gap/cgi-bin/study.cgi?study_id=phs001684.v3.p1. Individual-level data are available indefinitely. Processed data files are available on GitHub: https://github.com/zinterm/pedBMT_BALseq

Research involving human participants, their data, or biological material

Policy information about studies with human participants or human data. See also policy information about sex, gender (identity/presentation), and sexual orientation and race, ethnicity and racism.

Reporting on sex and gender	Patient sex was collected and determined by medical records according to biological sex assigned at birth. Patient sex was reported in Table 1 for all study participants and is listed in the metadata accompanying the sequencing files that have been deposited in NIH's dbGaP with controlled-access permission for sharing individual level data. 133 of 229 patients were male (58%); the slight over-representation of males in the study cohort is common in studies of pediatric stem cell transplant cohorts due to the increased prevalence of X-linked disorders requiring transplantation. Patient sex was considered in analyses and reported when associated with biological differences or differences in clinical outcomes (e.g.: line 208, females were over-represented in 2 BAL clusters associated with worse clinical outcomes; e.g. line 217, sex was included as a confounding covariate in a Cox regression model for in-hospital mortality).
Reporting on race, ethnicity, or other socially relevant groupings	Race and ethnicity were collected and determined by medical records (which are populated by patient/family self-reporting). Categorization of race and ethnicity were determined by NIH criteria at the time of submission. Race was categorized as White/Caucasian, Black/African-American, Asian/Pacific Islander, Native American, Other/Multiple, or Unknown. Ethnicity was separately reported as Latino/Hispanic yes/no and was collected as a separate variable. Race and ethnicity are reported in Table 1 for all study participants and is listed in the metadata accompanying the sequencing files deposited in dbGaP. No biological differences or differences in clinical outcomes were identified in association with race or ethnicity.
Population characteristics	We describe patient age, sex, race, and ethnicity, underlying disease (reason for stem cell transplantation), characteristics of the transplant process (allograft source, donor, HLA match, conditioning chemotherapy regimen, and days between transplant to study enrollment), and immune function at the time of enrollment (as measured by blood lymphocyte and neutrophil counts).
Recruitment	Patients and/or their parents/surrogates were approached for study consent by members of the study team at each participating children's hospital. Study teams consisted of at least one physician specializing in either stem cell transplantation, intensive care, or infectious disease, as well as at least one research coordinator trained in study protocols. For non-English speaking patients, language translation services were used according to local resources, including translated paper copies of consent forms and in-person, phone, or video interpretor services. At the University of California, consent forms were translated in Spanish. Selection may have been biased by limited availability of research staff for evening, weekend, and holiday enrollments, as well as for non-English or Spanish speaking patients.
Ethics oversight	Patients or their guardians were approached prospectively for consent under local IRB approval at each site (UCSF IRB #14-13546, #16-18908; Utrecht #05/143 and #11/063) in accordance with the Declaration of Helsinki and permission was obtained to collect leftover BAL fluid. at the participating sites. After approval at UCSF, study protocols were approved at each participating site as listed in Supplementary Data File 1.1. Use of reliance agreements on a single IRB was not available as a tool for consolidating IRB processes at the time.

Note that full information on the approval of the study protocol must also be provided in the manuscript.

Field-specific reporting

Please select the one below that is the best fit for your research. If you are not sure, read the appropriate sections before making your selection.

☒ Life sciences ☐ Behavioural & social sciences ☐ Ecological, evolutionary & environmental sciences

For a reference copy of the document with all sections, see nature.com/documents/nr-reporting-summary-flat.pdf

Life sciences study design

All studies must disclose on these points even when the disclosure is negative.

Sample size	The target study sample size was determined based on 2 methods.<br><br>First, in order to create a multivariable model with the outcome of in-hospital mortality and seven important potential predictors (age,

gender, HCT indication, conditioning regimen, transplant type, GVHD grade, and level of immunocompromise), we estimated an effect size f^2=0.1 with 7 degrees of freedom and β=0.2. We chose a Bonferroni-corrected α=0.007 without correlation to produce an overall α=0.05. This model would require n=219 to detect significance in all seven predictors (G*Power 3.1.9.2).

Second, in order to compare metagenomics and conventional hospital-based tests for infection, we assumed that traditional microbiology tests would have a pathogen yield of 25% (based on the literature), that NGS would identify pathogens in 40% of patients, and that 5% of our cohort would have positive findings on traditional clinical microbiology tests but negative findings on NGS. The latter may be due to sample degradation or other factors. This results in 25% discordant pairs and produces an effect size odds ratio of 4, meaning that NGS would have 4 times the odds of identifying a new microbe compared to missing a detected microbe. Assuming α=0.05 and β=0.2, McNemar's Test requires a minimum of 80 patients to detect a statistically significant difference between these tests (G*Power 3.1.9.2), with 95% confidence interval of the effect size being 1.3-16.4 (Stata 13.1). We determined that a lower limit of 2 for the confidence interval would be important when comparing an experimental diagnostic to an established and widely present method, as the proposed test would need to be considerably better to merit replacing the status quo. A sample size of at least n=200 would be required for an effect size odds ratio 4.0 with 95% CI 2.0-9.0 (STATA 13.1).

Therefore, the goal accrual was 250 HCT patients. We anticipated that site enrollment would vary by center volume, case mix, and practice variation in both obtaining BAL and timing of endotracheal intubation if indicated. We anticipated that centers with 0-20, 21-40, 41-60, 61-80, and 80+ transplants/year would generate approximately 1, 2, 4, 6, or 8 enrollments/year, respectively. Assuming an even mix of center size, we anticipated needing approximately 25 centers per year to meet our annual accrual goal.

Data exclusions

Per line 548 of the Methods section, "Since the input RNA mass of the water controls was determined to be about (5 pg presumably reflecting 5 pg of sequenceable contamination), we discarded samples whose total input mass was below 10 pg, as we were unable to reliably differentiate between contamination and true constituents." This amounted to 4 enrolled patients In addition, we excluded 19 patients who were enrolled prior to BAL, but BAL was either not performed, there was not adequate sample for research, or samples were destroyed or thawed prior to receipt at UCSF.

Replication

A geographically distinct validation cohort of pediatric stem cell transplant recipients from the University Medical Center in Utrecht, the Netherlands was identified and used to successfully replicate the associations between microbiome, gene expression, and mortality.

Randomization

Not applicable for an observational, non-interventional study.

Blinding

Creation of the BAL clusters was performed using unsupervised methods that are agnostic to patient outcomes.

Reporting for specific materials, systems and methods

We require information from authors about some types of materials, experimental systems and methods used in many studies. Here, indicate whether each material, system or method listed is relevant to your study. If you are not sure if a list item applies to your research, read the appropriate section before selecting a response.

Materials & experimental systems

n/a	Involved in the study
☒	☐ Antibodies
☒	☐ Eukaryotic cell lines
☒	☐ Palaeontology and archaeology
☒	☐ Animals and other organisms
☐	☒ Clinical data
☒	☐ Dual use research of concern
☒	☐ Plants

Methods

n/a	Involved in the study
☒	☐ ChIP-seq
☒	☐ Flow cytometry
☒	☐ MRI-based neuroimaging

Clinical data

Policy information about clinical studies
All manuscripts should comply with the ICMJE guidelines for publication of clinical research and a completed CONSORT checklist must be included with all submissions.

Clinical trial registration

n/a (not clinical trial)

Study protocol

All methods are included in the manuscript. The exact study protocol and REDCap database design with variable definitions is available upon request.

Data collection

Patients were enrolled from 2014-2022 and data were collected at 32 children's hospitals in the United States, Canada, and Australia (Supplemental Data, eTable 1). In addition, patients were enrolled from 2005-2016 and data were collected at 1 children's hospital (University Medical Center) in Utrecht, the Netherlands.

Outcomes

The primary outcome for the primary multi-center cohort was in-hospital mortality, defined as death prior to discharge alive. For the secondary validation cohort from the Netherlands, the outcome was non-relapse mortality at 1 year post BAL. There was no loss-to-followup for any patients.

Plants

Seed stocks	n/a
Novel plant genotypes	n/a
Authentication	n/a
